# The contribution of the genomes of a termite and a locust to our understanding of insect neuropeptides and neurohormones

**DOI:** 10.3389/fphys.2014.00454

**Published:** 2014-11-19

**Authors:** Jan A. Veenstra

**Affiliations:** INCIA UMR 5287 CNRS, Université de BordeauxPessac, France

**Keywords:** vasopressin, neuroparsin, neuropeptide, calcitonin, receptor

## Abstract

The genomes of the migratory locust *Locusta migratoria* and the termite *Zootermopsis nevadensis* were mined for the presence of genes encoding neuropeptides, neurohormones, and their G-protein coupled receptors (GPCRs). Both species have retained a larger number of neuropeptide and neuropeptide GPCRs than the better known holometabolous insect species, while other genes that in holometabolous species appear to have a single transcript produce two different precursors in the locust, the termite or both. Thus, the recently discovered CNMa neuropeptide gene has two transcripts predicted to produce two structurally different CNMa peptides in the termite, while the locust produces two different myosuppressin peptides in the same fashion. Both these species also have a calcitonin gene, which is different from the gene encoding the calcitonin-like insect diuretic hormone. This gene produces two types of calcitonins, calcitonins A and B. It is also present in Lepidoptera and Coleoptera and some Diptera, but absent from mosquitoes and *Drosophila*. However, in holometabolous insect species, only the B transcript is produced. Their putative receptors were also identified. In contrast, *Locusta* has a highly unusual gene that codes for a salivation stimulatory peptide. The *Locusta* genes for neuroparsin and vasopressin are particularly interesting. The neuroparsin gene produces five different transcripts, of which only one codes for the neurohormone identified from the corpora cardiaca. The other four transcripts code for neuroparsin-like proteins, which lack four amino acid residues, and that for that reason we called neoneuroparsins. The number of transcripts for the neoneuroparsins is about 200 times larger than the number of neuroparsin transcripts. The first exon and the putative promoter of the vasopressin genes, of which there are about seven copies in the genome, is very well-conserved, but the remainder of these genes is not. The relevance of these findings is discussed.

## Introduction

Neuropeptides and neurohormones are important regulators of physiological processes that may act at the periphery or within the central nervous system. They are evolutionarily very old and consequently orthologs are present in both deuterostomes and protostomes (Mirabeau and Joly, 2013). Their structures are often insufficiently conserved to establish homology. However, most neuropeptides act through G-protein coupled receptors (GPCRs) and due to the co-evolution between the receptors and their ligands (Park et al., 2002), evolutionary relationships between different peptides can be confirmed using the sequences of the transmembrane regions of the GPCRs they activate (Mirabeau and Joly, 2013). Whereas the structures and the immediate functions, i.e., activation of specific GPCRs, are conserved, their physiological effects may be quite different. For example, the neuropeptide SIFamide is expressed in *Drosophila* exclusively in four brain neurons that modulate sexual behavior (Terhzaz et al., 2007), while in ticks the same peptide acts on the salivary glands and the hindgut (Šimo et al., 2009, 2013; Šimo and Park, 2014). It is this what makes the study of neuropeptides so interesting, as it allows one to see how the endocrine and nervous systems and pathways may evolve over time, thereby ultimately contributing to a better understanding as to the origin of our own nervous and endocrine systems.

After the initial genome sequence of *Drosophila* (Adams et al., 2000) a significant number of arthropod genomes have followed suit, most of them from holometabolous insect species. The three hemimetabolous insect species with a completely sequenced genome are the louse (Kirkness et al., 2010), the pea aphid (Richards et al., 2010) and the as yet unpublished genome of the kissing bug *Rhodnius prolixus*. An impressively complete neuropeptide transcriptome of the brown plant hopper *Nilaparvata lugens* has also recently been published (Tanaka et al., 2014). All four of those species are very specialized feeders and therefore not necessarily representative of this group. The termite *Zootermopsis nevadensis* and the migratory locust *Locusta migratoria* both belong to more basal insect groups and as their genomes have recently been sequenced (Terrapon et al., 2014; Wang et al., 2014), it seems valuable to look at the neuropeptides they encode. A preliminary list of neuropeptide genes present in the *Zootermopsis* has already been published (Terrapon et al., 2014), but the actual number of such genes is much larger and here we also identify the neuropeptide GPCRs of this species. *Locusta* is probably the insect species from which the largest number of neuropeptides has been biochemically identified, by the Leuven group (Schoofs et al., 1990a,b,c,d,e, 1991a,b, 1992a,b, 1993a,b, 1994; Paemen et al., 1991a,b; Veelaert et al., 1995; Tawfik et al., 1999; Clynen et al., 2001, 2003a,b,c, 2006; Clynen and Schoofs, 2009) and by many others who identified specific neuropeptides, such as the various adipokinetic hormones (Stone et al., 1976; Siegert et al., 1985; Oudejans et al., 1991; Bogerd et al., 1995; Siegert, 1999), the vasopressin-like peptide (Proux et al., 1987), neuroparsins (Girardie et al., 1989), the cortiocotropin-releasing factor (CRF)-like diuretic hormone (Kay et al., 1991; Lehmberg et al., 1991), ovary maturating parsin (Girardie et al., 1991), FMRFamide (Lange et al., 1994; Hill and Orchard, 2007) and periviscerokinin (Predel and Gäde, 2002). Ion transport peptide was identified from a different migratory locust, *Schistocerca gregaria* (Meredith et al., 1996). Obviously, the locust genome is of particular interest as far as neuropeptides are concerned.

In the present paper, we describe the genes coding several neuropeptides from these two insect species, and the GPCRs of these neuropetides in the termite. We also discuss the discovery of novel neuropeptide genes, which have not been identified previously, i.e., those encoding SMYamide, calcitonin, tryptopyrokinins, and a salivation stimulating peptide, as well as two locust neuropeptide genes that are of particular interest, the neuroparsin and vasopressin genes.

## Materials and methods

The *Zootermopsis* genome was downloaded from http://www.termitegenome.org and was analyzed by local BLAST (Altschul et al., 1997) on a desktop PC. The *Locusta* genome was searched using the NCBI web interface and interesting contigs were downloaded for a more detailed analysis. Preliminary prediction of gene models or parts thereof was based on homology with known insect neuropeptides and their receptors in combination with likely intron splice sites using Artemis (Rutherford et al., 2000) as described previously (Veenstra et al., 2012). All short sequence read archives (SRAs, both genome and transcriptome data) for these two species that were available at NCBI were downloaded and the SRA toolkit (http://www.ncbi.nlm.nih.gov/Traces/sra/?view=software) was used to extract the data in fasta form, which was then used to build searchable BLAST databases using BLAST+ (Camacho et al., 2009). The initially predicted mRNA sequence was used to search all the transcriptome SRAs for that species. Positive reads were collected and used as input for the Trinity program (Haas et al., 2013) in order to produce transcripts that were used to improve and correct the initial predictions using Artemis. This was an effective method to determine N- and/or C-terminal regions of proteins that could not be predicted by homology alone and in cases where there were gaps either within the contigs or within the scaffolds. The Trinity-Artemis cycle was repeated as long as the coding regions of the predicted mRNA sequences were incomplete and the predicted mRNA's increased in length. No attempt was made to accurately determine the 3′- and 5′- ends of mRNAs; read-throughs from neighboring genes easily give false positives. For some neuropeptides that were previously isolated and identified from *Locusta* no DNA sequences were found in the genome assembly. In those cases we looked in the genomic SRAs in order to find individual reads that might contain sequences potentially coding these peptides but that had not made it into the assembled genome. We then used these sequences to search the genomic SRAs for similar sequences and attempted to assemble those reads into mini-contigs using both edena (Hernandez et al., 2008) and velvet (Zerbino and Birney, 2008), but this was not very successful.

For comparative purposes, a few other insect genomes both published and unpublished were prospected for specific neuropeptide precursors or GPCRs. Preliminary assemblies of the genomes of *Blattella germanica, Ladona fulva, Ephemera danica, and Lutzomyia longipalpis* were downloaded from https://www.hgsc.bcm.edu/arthropods/ and those of *Phlebotomus papatasi* and *Diaphorina citri* from https://www.vectorbase.org/ and http://www.psyllid.org/, respectively. Previously published annotated neuropeptide GPCRs from *Drosophila, Apis, Tribolium* (Hauser et al., 2008), *Bombyx* (Yamanaka et al., 2008), a spider mite (Veenstra et al., 2012), and a plant hopper (Tanaka et al., 2014) were added to the curated *Zootermopsis* GPCRs for the construction of a phylogenetic tree. A few deorphanized protostomian neuropeptide GPCRs were appended to this ensemble. Phylogenetic trees were made after using MUSCLE (Edgar, 2004) and/or Clustal Omega (Sievers et al., 2011) for obtaining sequence alignments on the desktop that were manually inspected and corrected using Seaview (Gouy et al., 2010). Seaview was also used for selecting the conserved protein regions used for making the trees. Both PhyML (Guindon and Gascuel, 2003) and FastTree 2 (Price et al., 2010) were used for making trees. As differences between the two were minimal, only the latter was used for the larger ones. Convertase cleavage site predictions were guided by rules previously described (Veenstra, 2000) and signal peptides were analyzed with Signal P 4.0 (Petersen et al., 2011), but the signal peptide of the *Locusta* ion transport peptides were predicted by the hidden Markov model of Signal P 3.0; the neural network models of both Signal P 3.0 and 4.0 suggest that these peptides do not have a signal peptide. The signal anchor of the termite allatostatin CC was predicted by Signal P 3.0 (Bendtsen et al., 2004) and confirmed by the forecast of a transmembrane region by a hidden Markov model (Krogh et al., 2001).

## Results

The data for *Zootermopsis* allowed us to predict complete precursor sequences for virtually all known insect neuropeptides, including such recent additions as the CCHamides, ACP, RYamide, trissin, natalisin, and CNMa (Roller et al., 2008; Hansen et al., 2010; Hauser et al., 2010; Ida et al., 2011a,b, 2012; Jiang et al., 2013; Jung et al., 2014). In a number of cases, exons were lacking either completely or partially from the genome assemblies, but all these gaps could be filled and/or corrected with transcriptome data and in a few cases those corrections were confirmed with the genomic reads. The predicted transcripts for the neuropeptide GPCRs seem similarly very complete. The *Locusta* genome is much bigger, giving rise to much bigger introns, and a couple of genes appear to be represented by different alleles. Furthermore, the *Locusta* transcriptome data is much more limited and for some genes non-existent. Thus, the pyrokinin, and vasopresssin genes are known to be expressed exclusively or predominantly in the suboesophageal ganglion (Rémy and Girardie, 1980; Clynen and Schoofs, 2009) and we expect tryptopyrokinins to be expressed also mainly in this ganglion. However, none of these genes is represented in the trancriptome SRAs, suggesting the absence of this ganglion from the trancriptome data. The difficulty to assemble repetitive sequences using a short read sequencing technology also affected the assembly of the *Locusta* genome more than the *Zootermopsis* genome, particularly in the case of the vasopressin genes, present in various copies, as well as some genes that code highly similar or identical multiple copies of the same peptide, such as the calcitonin gene.

The major reason for the less complete *Locusta* sequences is the lack of sufficient transcriptome data; the number of reads was often insufficient for Trinity to build transcripts. The use of individual transcriptome reads and publicly available expressed sequence tags (ESTs) from *Locusta* (Kang et al., 2004; Clynen et al., 2006; Ma et al., 2006; Chen et al., 2010) allowed us to fill some gaps, but many remain. It is for this reason that we did not attempt to predict the neuropeptide GPCRs in this species (in a few cases we used partially constructed GPCRs for phylogenetic tree analysis). Although some genes are thus incompletely present in the *Locusta* genome assembly, most of the neuropeptides previously identified from the migratory locust were found in the genome sequences, if not in the assembly itself, at least in the genomic SRAs. Thus, in spite of the fragmentary nature of the *Locusta* genome (1,397,492 contigs vs. 64,771 for *Zootermposis*) and the limited amount of transcriptome data, we were able to deduce the complete sequences of a impotant number of *Locusta* neuropeptide precursors, while for others at least a significant part of their precursor was found.

In a few cases the sequences predicted by the *Locusta* genome are slightly different from those previously reported. As most these differences are easily explained by experimental errors such as contamination of first amino acid residues in Edman degradation and/or the same or very similar molecular masses in mass spectrometry, it seems likely that the genomic sequences are the correct ones. The genomic sequences also allowed us to confirm the sequence of locustamyoinhibin (Schoofs et al., 1994) as allatostatin C.

We found a total of 59 and 63 different transcripts for neuropeptides and neurohormones in the genomes of *Zootermopsis* and *Locusta*, respectively. In the locust the actual number is probably even higher as there are likely to be several transcripts encoding the vasopressin-like peptide, but these could not be reliably identified. As several of these transcripts are predicted to produce more than one peptide the number of neuropeptides produced by these insects is much larger. The predicted precursors of these genes are illustrated in the Supplementary Figures 1, 2, and their sequences are listed in Supplementary Tables 1, 3.

More important than the actual number of different peptides produced is the question as to how many different messages they can transmit. It is plausible that the number of different neuropeptide receptors will give a more accurate answer to that question. In the *Zootermopsis* genome a total of 65 putative neuropeptide GPCRs were identified. Although a large number of them can be deorphanized *in silico* by their close structural similarity to deorphanized insect neuropeptide GPCRs, others are orphans, which may not be neuropeptide receptors. On the other hand some neuropeptides are known to act on receptors that are not GPCRs, such as insulin, eclosion hormone and at least one of the peptides encoded by the NPLP1 gene in *Drosophila* (Chang et al., 2009; Overend et al., 2011; Vogel et al., 2013). The predicted *Zootermopsis* neuropeptide and neurohormone GPCRs are presented in Supplementary Table 2.

On phylogenetic trees (Supplementary Figures 3, 4), most of the GPCRs cluster with related GPCRs from other species and in many cases the receptor can be deorphanized *in silico* due to the fact that for one or more of the same group the ligand has been determined experimentally. It is interesting to see that some receptors are only present in *Zootermopsis, Nilaparvata*, and/or *Tetranychus*, suggesting that the particular receptor and perhaps its ligand may have been lost in holometabolous insect species. In other cases, there may be two receptors for the same neuropeptide. For example, in the case of *Zootermopsis* GPCR A51 and *Nilaparvata* GPCRs A38 and A39 it is tempting to speculate that they might be alternative NPF receptors, while *Zootermopsis* GPCR A11 and *Nilaparvata* GPCR A5 might be SIFamide (or SMYamide?) receptors.

### Accessory gland myotropin II

**Figure 1:**
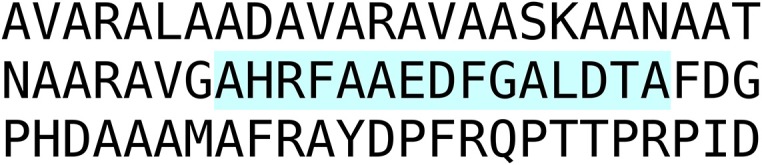
***Locusta* accessory gland myotropin**. Deduced amino acid sequence from EST GO240796.1 and genome assembly. Both the genome assembly and the EST have a few more amino acids at the N-terminus, but they differ, probably due to the insertion or deletion of a nucleotide. The sequence shown here is identical between the genome assembly and the EST. It clearly shows that the isolated active peptide (Paemen et al., 1991b), shown in blue, is not flanked by classical neuropeptide convertase cleavage sites.

An unusual peptide identified in Leuven concerns the accessory gland myotropin II, which was identified from male accessory glands in *Locusta* (Paemen et al., 1991b). The sequence of this peptide is encoded on an EST (Accession # GO240796). The same sequence can also be deduced from genomic and transcriptomic data and shows a precursor that does not look like a neuropeptide precursor as it is missing typical neuropeptide convertase cleavage sites (Figure 1).

### Adipokinetic hormone

Adipokinetc hormone (AKH) was identified from migratory locusts as a hormone from the corpora cardiaca increasing hemolymph lipid during flight (Beenakkers, 1969; Mayer and Candy, 1969). It is made in the glandular cells of the corpora cardiaca and released during flight. After the identification of the decapeptide AKH I (Stone et al., 1976), the octapeptides AKH II and AKH III were identified (Siegert et al., 1985; Oudejans et al., 1991). The closely related peptide ACP (AKH/corazonin-related peptide) was initially also considered to be an AKH (Siegert, 1999), but has now been shown to be a different peptide with its own receptor (Hansen et al., 2010). Unlike AKH it is not expressed in the glandular cells of the corpora cardiaca but by neurons in the nervous system (Hansen et al., 2010; Patel et al., 2014). In the *Locusta* genome the genes encoding AKH I, II, and III as well as ACP were identified. Interestingly, a fourth AKH gene was encountered, which we called AKH IV. Based on the number of transcriptome reads, AKH IV may be even less expressed than AKH III (3 vs. 6 reads), which is much less abundantly expressed than AKHs I and II (586 and 21 reads, respectively). In the *Zootermopsis* genome only a single AKH gene was found, as in many other insect species.

### Allatostatin CC

Allatostatin CC has strong sequence similarity to allatostatin C and based on this was expected to activate the same receptors (Veenstra, 2009), as now has been confirmed for the beetle *Tribolium castaneum* (Audsley et al., 2013). Allatostatin CC does not appear to be a typical neuropeptide and it was suggested that it might function as a paracrine secretion, not only secreted by neurons but also by other cells (Veenstra, 2009). The expression of this peptide by hemocytes (Accession # FP353238.1, FP357967.1, FP358784.1, FP359038.1, FP352222.1, FP352474.1) supports the hypothesis that the peptide is indeed made by non-neuroendocrine cells. In the *Drosophila* species as well as tsetse flies allatostatin CC lacks a signal peptide and instead has a signal anchor, reinforcing the notion that it is released locally and not systemically. In the termite the gene similarly encodes a signal anchor, but in the locust it is predicted to have a classical signal peptide. Therefore, it appears to be a juxtacrine secretion also in termites. A peptide secreted as a paracrine or juxtacrine rather than a endocrine presumably would not need as high an affinity for its receptor as a hormone. This is indeed what was found for *Tribolium* (Audsley et al., 2013).

### Calcitonin and calcitonin-like diuretic hormone

Anything that stimulates the excretion of water in insects could potentially be used as the basis for designing a novel pesticide and putative insect diuretic hormones have therefore received a lot of attention. One of the identified diuretic hormones has been called the calcitonin-like diuretic hormone but is also known by the abbreviation DH31. This peptide was first identified from the cockroach *Diploptera punctata* (Furuya et al., 2000) and genes coding such a peptide are present in all insect genomes. The *Drosophila* and *Rhodnius* homologs have been shown to activate B-type GPCR's that are related to the vertebrate calcitonin receptors (Johnson et al., 2005; Zandawala et al., 2013). This hormone is generally considered to be the insect calcitonin ortholog. However, the genomes of both *Zootermopsis* and *Locusta* contain another gene that encodes peptides showing even stronger similarity to calcitonin, as they contain a predicted disulfide bridge.

**Figure 2:**
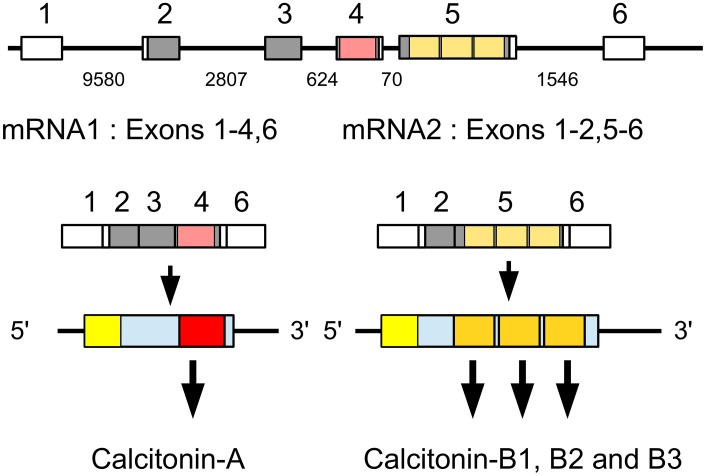
**Schematic representation of alternative splicing of the *Zootermopsis* calcitonin gene**. On top the six exons indicated by boxes; only exons 2–5 are coding exons; non-codings exons are white. Numbers between the exons indicate the number of nucleotides that separate them. mRNA1 consists of all exons except the fifth and leads to a precursor from which a single calcitionin A is produced. mRNA2 lacks the third and the fourth exons and this will lead the prodcution of the calcitonin B precursor, from which three calcitonin B's are predicted. Location of various calcitonins within the precursor, the mRNAs and the respective exons has been indicated by red and orange and the signal peptide in yellow.

**Figure 3:**
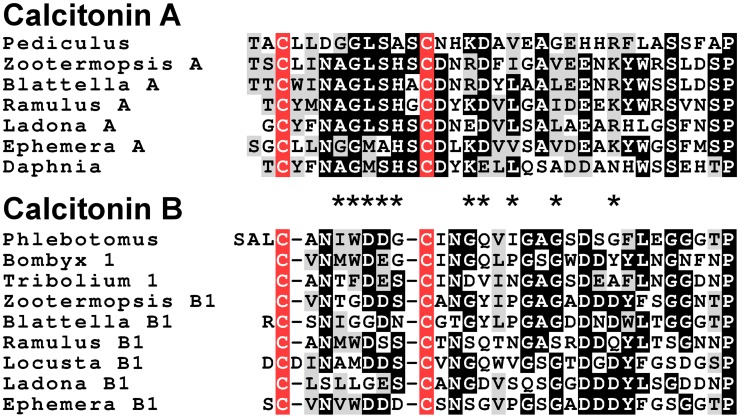
**Alignment of the insect calcitonins A and B**. Note that the overall structures of these peptides are similar, as they have a disulfide bridge at the N-terminus as well as a C-terminal Pro-amide. Nevertheless, several amino acid residues, indicated by asteriks, in homologous positions are signficantly different.

Analysis of several other arthropod genomes, ESTs and transcriptome shotgun assemblies (TSAs) show this gene to be very generally present in arthropods (Supplementary Figure 5). Comparing the different encoded arthropod calcitonins reveals that they can be separated into two distinct classes, which we have called calcitonin-A and calcitonin-B (Supplementary Figure 6). In the termite this gene produces two different transcripts (Figure 2). The first one, the A-transcript, codes for a single calcitonin-A, while the B-transcript encodes three calcitonin-B peptides. It must be noted that even though both types of calcitonin have a predicted N-terminal disulfide bridge and a C-terminal Pro-amide sequence, the remainder of the sequences show striking differences (Figure 3). The as yet unpublished genomes of the dragonfly *Ladona fulva*, the German cockroach *Blattella germanica* and the may fly *Ephemera danica* have very similar genes, although the number of copies of calcitonin-B on the second transcript is variable. Both types of peptides are also produced in phasmids as TSAs from various species attest.

The *Locusta* calcitonin gene has a gap in its sequence and no calcitonin-A peptide was found in the genome assembly, however in the genomic sequence reads a sequence was found that encodes a calcitonin-A-like peptide, i.e., CYIGGRMGGCDYQDLKQAQGEDQHLNSIDSPGKR.

Although it is notably absent from *Drosophila* and other flies as well as mosquitoes, aphids, *Rhodnius*, and Hymenoptera, it is present in the sand flies *Phlebotomus papatasi* and *Lutzomyia longipalpis* as well as beetles and Lepidoptera. Many of the predicted arthropod calcitonin precursors contain several copies of this neuropeptide, while the *Tribolium* genome has even two different genes (Supplementary Figure 5). Transcriptome data show calcitonin B to be expressed in the insect midgut.

**Figure 4:**
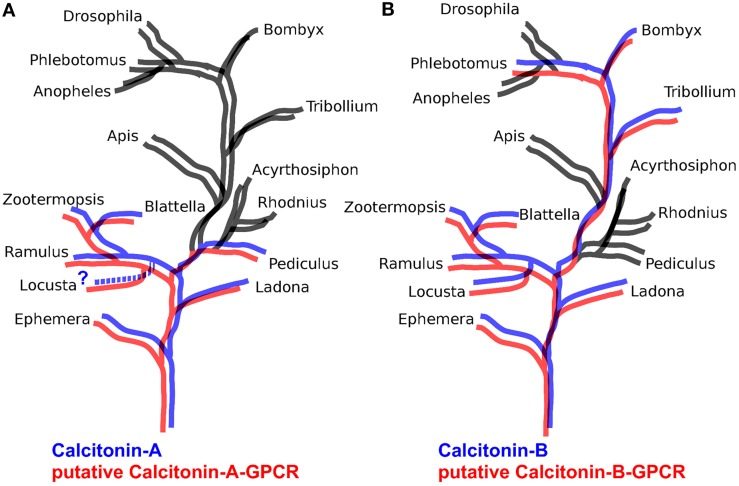
**Schematic phylogenetic trees of insects illustrating the presence or absence of Calcitonin A and B in blue and their putative receptors in red**. When a peptide or a receptor is absent, branches are black. Note that in **(A)** calcitonin A is present in the same species as its putative receptor while in **(B)** the same holds for calcitonin B.

The B-type GPCRs can be expected to include the receptor(s) for the arthropod calcitonins described here. Of the B-type GPCRs that have not been deorphanized in insects, there is one that appears to be the best candidate. This group of receptors is characterized by the B3 receptor from *Bombyx* (Yamanaka et al., 2008) and the receptor identified as 72 from *Tribolium* (Hauser et al., 2008) and corresponds to cluster A of Cardoso et al. (2014). We thus looked for homologs of this receptor in other arthropod genomes and found such receptors only in those species that also have a calcitonin gene. Given our interest in the *Locusta* receptors, we included two partial homologs from this species. The results suggests that in basal insect groups there are two calcitonin receptors, like in *Locusta, Zootermopsis*, and the stick insect *Ramulus artemis* (Supplementary Figure 4). Interestingly, phylogenetic trees for the calcitonin-A peptides correspond completely with one of these receptors, while tree for the calcitonin-B peptides corresponds perfectly with the other (Figure 4).

### Capa peptides, pyrokinins, periviscerokinins, and tryptopyrokinins

The nomenclature of these peptides is not always very clear, in large part because sequences of these peptides are rather similar and peptides have often been named according to the tissue they have been identified from or the gene on which they are encoded. Analysis of their receptors in *Drosophila* has made it clear that pharmacologically one can distinguish three different types of peptides and this has been confirmed in other species (Iversen et al., 2002; Rosenkilde et al., 2003; Cazzamali et al., 2005; Homma et al., 2006; Paluzzi et al., 2010; Paluzzi and O'Donnell, 2012). The pyrokinins and tryptopyrokinins have C-terminal consensus sequences of FXPRLamide and MWFGPRLamide, respectively, while that of the periviscerokinins is FPRVamide.

The tryptopyrokinins seem to be a relatively recent evolutionary innovation, as they have so far not been found in non-insect arthropods. The close association of their receptors with those for pyrokinins on evolutionary trees for GPCRs suggests the same evolutionary innovation (Paluzzi and O'Donnell, 2012). In a few holometabolous insect species, tryptopyrokinins are known to be expressed specifically by neuroendocrine cells in the labial neuromere of the suboesophageal ganglion. At least in the case of *Drosophila* this is achieved by differential processing of the capa precursor (Wegener et al., 2006) and it has previously been suggested that such a differential processing might also occur in *Bombyx* (Veenstra, 2000).

**Figure 5:**
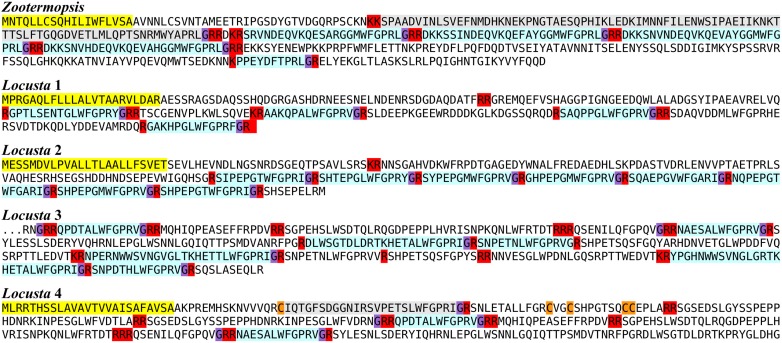
**Tryptopyrokinins**. The predicted *Zootermopsis* tryptopyrokinin precursor is expected to yield at least four tryptopyrokinins and one pyrokinin. Of the four predicted *Locusta* tryptopyrokinin precursors only one contains a pyrokinin.

Insects generally have two genes coding for these peptides, a capa gene coding for periviscerokinins and often a tryptopyrokinin, and a pyrokinin gene that encodes pyrokinins and often a tryptopyrokinin. Both *Zootermopsis* and *Locusta* have a third type of gene that codes for tryptopyrokinins and in the case of the termite a single pyrokinin. Interestingly, the locust has four such genes (Figure 5).

The *Locusta* pyrokinin gene was one of the most difficult genes to analyze. Some of the genomic reads suggest that there may be two alleles represented in the genomic reads while transcriptome reads from the suboeosophageal ganglion, where this gene is normally predominantly expressed, are lacking from the expression SRAs. Most, but not all, of the pyrokinins previously identified from this species (Schoofs et al., 1990e, 1991a, 1992b, 1993b; Clynen et al., 2003a,b) were found in individual reads of the *Locusta* genome.

The periviscerokinin transcript is probably also incomplete, the part found encodes only two peptides, periviscerokinin, and TSSLFPHPRLamide, both previously identified (Predel and Gäde, 2002; Clynen et al., 2003a,b). From the distribution of these peptides and that of DGAETPGAAASLWFGPRVamide and GLLAFPRVamide (Clynen and Schoofs, 2009), one would expect to find the latter two also to be expressed by the periviscerokinin gene, but neither genomic nor transcriptomic reads encoding these peptides were found.

### CNMa

**Figure 6:**
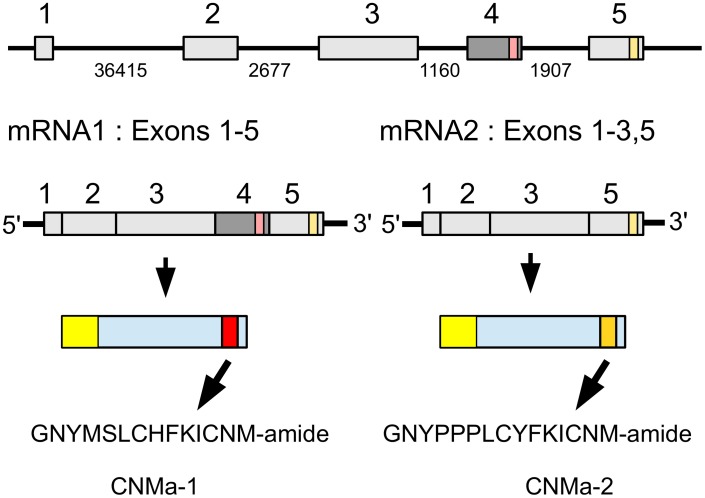
**Schematic representation of alternative splicing of the *Zootermopsis* CNMa gene and the different CNMa precursors it produces**. On top the five coding exons indicated by boxes, numbers between the exons indicate the number of nucleotides that separate them. mRNA1 consists of all five coding exons and leads to a precursor from which CNMamide 1 is produced. mRNA2 lacks the fourth coding exon and this will lead to the translation of the fifth coding exon and the production of CNMamide 2. Location of two CNMamides within the precursor, the mRNAs and the respective exons has been indicated by red and orange, respectively.

CNMa was very recently identified as a novel insect neuropeptide (Jung et al., 2014). The *Zootermopsis* CNMa gene produces by alternative splicing two types of mRNA that differ by the inclusion or exclusion of the third coding exon (Figure 6). When the third coding exon is included in the mature mRNA it should lead to the production of GNYMSLCHFKICNMamide. In that case the fourth coding exon will not be translated, since the third exon contains an in-frame stop codon near its end. When the third coding is excised from the mature mRNA, the fourth coding exon is predicted to produce the alternative CNMamide, i.e., GNPPPLCYFKICNM-amide. The number of transcriptome reads specific for the first transcript is much smaller (75) than that for the second (931) and it thus seems plausible that the second peptide is produced in larger amounts than the first. As in several Hymenoptera and *Nilaparvata* (Jung et al., 2014; Tanaka et al., 2014) the termite genome also contains two CNMamide receptor homologs that on phylogenetic trees cluster tightly with the de-orphanized CNMamide receptors and hence are most likely the *Zootermopsis* receptors for these neuropeptides (Supplementary Figure 3). Only a single CNMa peptide was found encoded by the orthologous *Locusta* gene.

### CRF-like diuretic hormone and ovary maturating parsin (OMP)

The insect CRF-like neuropeptides are other putative diuretic hormones and both the *Locusta* peptide and its *Zootermopsis* ortholog have been isolated and sequenced (Kay et al., 1991; Lehmberg et al., 1991; Baldwin et al., 2001). Ovary maturating parsins have been found exclusively in locusts, both *Locusta* and *Schistocerca* (Girardie et al., 1991, 1998). This hormone has been reported to stimulate oocyte growth and induce vitellogenin synthesis (Girardie et al., 1992, 1998; Girardie and Girardie, 1996). These two peptides are produced by the same neuroendocrine cells in the brain (Tamarelle et al., 2000). Recently the cDNA for *Schistocerca* CRF-like diuretic hormone was found to encode also OMP (Badisco et al., 2011; Van Wielendaele et al., 2012) and it is thus not surprising that the *Locusta* transcript similarly encodes both hormones. The diuretic hormone precursor from *Zootermopsis* does not contain a sequence that is recognizable as a homolog of ovary maturating parsin, as is the case with precursors of the CRF-like peptide in all other insect species (Van Wielendaele et al., 2012). RNAi knockdown of the common precursor of these hormones in *Schistocerca* had the opposite effect of what one would expect based on the reported biological activity of OMP. Inhibition of the production of OMP and the CRF-like diuretic hormone accelerated oocyte growth, while injection of the diuretic hormone inhibited it (Van Wielendaele et al., 2012). As far as the CRF-like peptide goes, these results are consistent, but they seem to contradict previous reports on OMP. It might indicate that ovary maturating parsin does not act like a neuropeptide through a GPCR, but that it has other, indirect effects at the doses injected. More work will be needed to resolve this question.

### Eclosion hormone

As in some other species, both the locust and the termite have two genes encoding an eclosion hormone. It seemed interesting to know whether this gene duplication is a relatively recent or a more ancient event. We used the BLAST website at NCBI to find DNA sequences that might code for such hormones and made a phylogenetic tree of the sequences. Results suggests that in some cases, such as *Locusta*, the butterfly *Danaus plexippus* and mosquitoes the duplication is relatively recent, but in the case of the termite, *Tribolium*, *Nilaparvata*, and the psyllid *Diaphorina citri* it looks like the second eclosion hormone gene is quite old (Supplementary Figure 7).

### Elevenin

**Figure 7:**
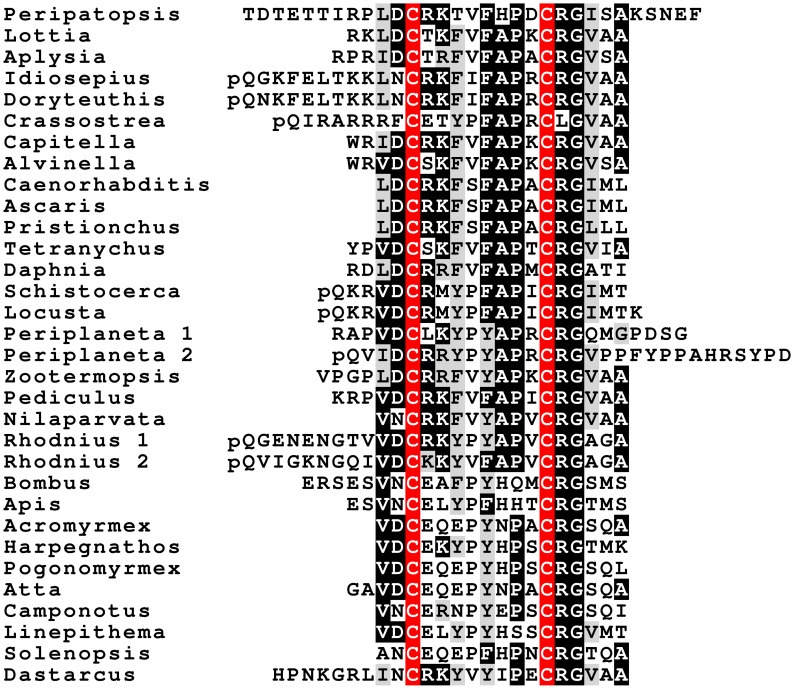
**Deduced amino acid sequences of protostomian elevenins**. Note that the peptide is not only present in insects, but also in velvet worms (*Peripatopsis*), mollusks (*Lottia, Aplysia, Idiosepius, Doryteuthis, Crassostrea*), polychaetes (*Capitella, Alvinella*), nematodes (*Caenorhabditis, Ascaris, Pristionchus*), crustaceans (*Daphnia*), and arachnids (*Tetranychus*). Also note that the sequences of the peptide in holometabolous insect species is less conserved.

Elevenin is a neuropeptide encoded on a mRNA initially identified from the L11 neuron in *Aplysia californica* (Taussig et al., 1984). Although the actual peptide has never been identified, similar neuropeptide precursors are common in mollusks and the peptide has been called elevenin (Veenstra, 2010a). Such neuropeptide precursors have also been deduced from the genome of *Caenorhabditis elegans* (Yamada et al., 2010) and a spidermite (Veenstra et al., 2012) as well as the trancriptome of the plant hopper *Nilaparvata* (Tanaka et al., 2014). Both the *Locusta* and *Zootermopsis* genomes contain an elevenin gene, which turns out to be present in quite a few species (Figure 7). Interestingly, a very large number of *Periplaneta* elevenin ESTs come from the testis of this species (Chen et al., 2013). As there is an intron in the middle of the sequence coding elevenin it is difficult to find it in genomes, particularly in those of holometabola where primary sequences appear more variable. Nevertheless, as the structure of the elevenin gene is well-conserved (Supplementary Figure 8), there is no doubt that the predicted peptides are authentic elevenin orthologs. Although we did find a single Coleopteran sequence from *Dastarcus helophoroides*, we were unable to find it in the *Tribolium* genome.

### FMRFamide and myosuppressin

**Figure 8:**
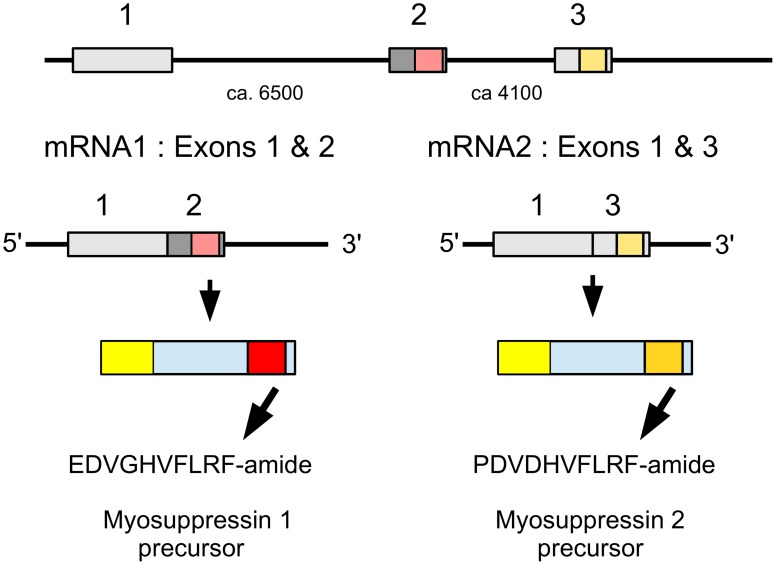
**Schematic representation of alternative splicing of the *Locusta* myosuppressin gene and the different myosuppressins it produces**. On top the three coding exons indicated by boxes, numbers between the exons indicate the approximate number of nucleotides that separate them. mRNA1 consists of coding exons 1 and 2 and leads to a precursor from which EDVGHVFLRFamide is produced. mRNA2 is made up of coding exons 1 and 3 and its ensuing precursor will yield PDVDHVFLRFamide. Location of two myosuppressins within the precursors, the mRNAs and the respective exons has been indicated by red and orange, respectively.

The termite myosuppressin gene is similar to that of other insect species, but in the locust the RNA produced from this gene is alternatively spliced into two mRNAs that each produce a different myosuppresin (Figure 8). Most of the mRNA (62 vs. 8 transcriptome reads) codes for PDVDHVFLRFamide, the peptide initially identified from *Schistocerca* (Robb et al., 1989) and later also from *Locusta* (Schoofs et al., 1993a; Peeff et al., 1994). The other mRNA codes for EDVGHVFLRFamide, a peptide almost identical to ADVGHVFLRFamide which was identified as a second myosuppressin from *Locusta* (Peeff et al., 1994). Both species also have a gene encoding FMRFamides. In the termite, it encodes six copies ending in NFIRFamide and four copies in NFVRFamide plus a number of other pepides. The *Locusta* FMRFamide transcript could not be reconstructed completely, but the part that was found codes for one FIRFamide and four FLRFamides, including GSERNFLRFamide. The latter peptide has a molecular mass very similar to LWENLRFamide, the sequence proposed for a peptide isolated from the gut of *Locusta* (Hill and Orchard, 2007) as well as GQERNFLFRamide, identified from the ventral nerve cord (Lange et al., 1994). Six transcriptome reads show that this sequence is part of a gene that is expressed.

### Insulin- and relaxin-like peptides

A single insulin gene was found in the migratory locust and six such genes were identified in the termite. One of the termite genes codes a peptide homologous to the *Drosophila* insulin-like peptide (dilp) 7. This peptide is significantly different from other insect insulin-like peptides and is predicted to bind a GPCR activated by relaxin-like hormones, rather than the classical insulin tyrosine kinase receptor (Veenstra et al., 2012). We have therefore called it relaxin-like, rather than insulin-like. It has two different transcripts that are predicted to yield very similar peptides.

### Neuroparsin

**Figure 9:**
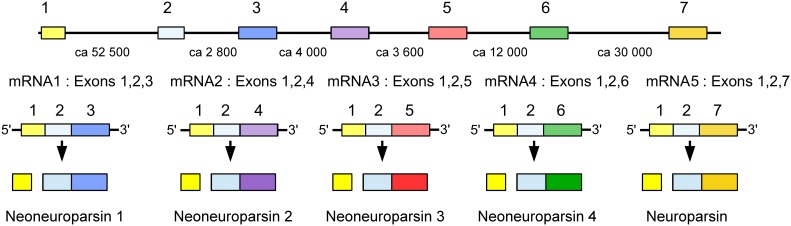
**Schematic representation of alternative splicing of the *Locusta* neuroparsin gene and the different transcripts originating from it**. On top the seven coding exons indicated by boxes, numbers between the exons indicate the approximate number of nucleotides that separate them. The five different mRNA's produced from this gene all share the first two coding exons and then add their specific exon to it. The first four transcripts encode a neoneuroparsin and the fifth transcript leads to the production of neuroparsin.

The *Zootermopsis* genome contains a neuroparsin gene with three coding exons that lead to the production of a single mRNA. The *Locusta* neuroparsin gene produces five different mRNAs (Figure 9). Transcript 5 is the one that encodes neuroparsin as it has been isolated from the corpora cardiaca (Girardie et al., 1989; Lagueux et al., 1992). The presence of five transcripts is similar to the situation in *Schistocerca gregaria* where four different neuroparsin mRNAs have been identified (Janssen et al., 2001; Claeys et al., 2003). The *Locusta* neuroparsin 5 transcript and the *Schistocerca* neuroparsin 1 transcript code for the neuroparsins that were isolated from the corpora cardiaca.

**Figure 10:**
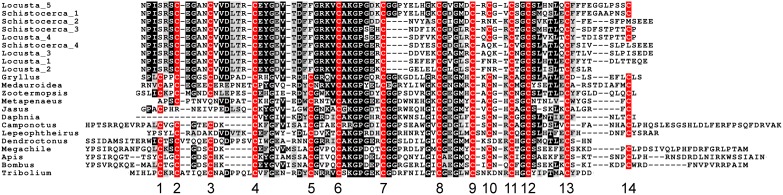
**Sequence alignment of neuroparsin and the neoneuroparsins with cysteine residues in red**. The *Locusta* transcript 5 and *Schistocerca* transcript 1 correspond to the isolated and identified neuroparsins, the other *Locusta* and *Schistocerca* transcripts are neoneuroparsins. Notice that although the spacing between the cysteine residues is more variable near the N- and C-terminal regions of these proteins, it is very constant in the core of the protein between cysteine residues 4 and 13. However, in the neoneuroparsin four residues are lacking between cysteines 7 and 8, suggesting that the three dimensional structures of these molecules may be different from that of the neuroparsins.

It is of interest to note that the proteins predicted from the *Locusta* neuroparsin transcripts 1–4 are lacking a four amino acid sequence in the middle of the molecule (Figure 10) and this is also the case for the other neuroparsin transcripts identified from *Schistocerca* (Janssen et al., 2001; Claeys et al., 2003). Although it is clear from earlier work that the neuroparsin primary sequences are very variable, notably in holometabolous insect species (Veenstra, 2010b), the neuroparsin core appears much better conserved (Figure 10). It is for this reason that we prefer to give the proteins predicted from these novel transcripts a different name and call them neoneuroparsins to distinguish them from the hormones identified from the corpora cardiaca.

Analysis of the expression of the various *Locusta* neuroparsin transcripts reveals that transcripts 1, 2, and 3 have much higher expression levels than those of 4 and 5. In all the *Locusta* transcriptome SRAs combined, there are 520,499,475 reads, of which 115,938 correspond to exons 1 and/or 2 (the exons that are common to all neuroparsin transcripts), 127,664 correspond to exons 3, 4 or 5, 116 to exon 6 and 203 to exon 7. In the SRA specific for the nervous system of *Locusta* (SRR167712) there are 28,946,371 individual reads, with 11,936 for exons 1 and/or 2, 12,637 for exons 3, 4 or 5, 33 for exon 6 and 66 for exon 7. These numbers show that a *very large majority* of neuroparsin transcripts is coding for one of the predicted neoneuroparsins 1, 2 or 3, whereas only a tiny minority codes for neuroparsin as identified from the corpora cardiaca.

### Neuropeptide-like precursors

**Figure 11:**
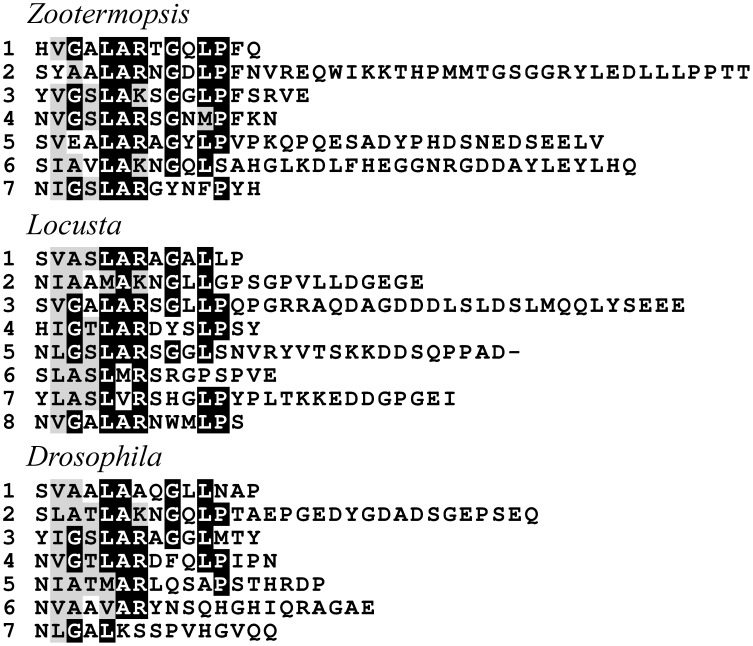
**Sequence alignment of some of the predicted mature peptides from the NPLP1 precursors of *Zootermopsis, Locusta*, and *Drosophila***. Note that although the sequences are highly variable, a consensus sequence can observed.

Using mass spectrometry four proteins were identified as precursors for peptides in the *Drosophila* CNS (Baggerman et al., 2002). Only one of them, neuropeptide-like precursor 1 (NPLP1), has typical neuropeptide convertase cleavage sites. NPLP1 orthologs were found in both the termite and the locust. In *Drosophila* the structure of these peptides seems very variable between the different peptides. However, when the different paracopies from *Zootermopsis* are aligned, a conserved N-terminal consensus sequence becomes visible (Figure 11). Similar sequences are recognizable in the precursors of *Locusta* and *Drosophila* NPLP1. *Drosophla* NPLP1-VQQ, the only NPLP1 derived peptide known to have biological activity (Overend et al., 2011), conforms only partially to this consensus sequence (Figure 11), so it is plausible that some of these peptides have a different active site.

Although obviously any peptide present in the brain can be called a neuropeptide, it is perhaps more likely that the other three precursors do not produce neuropeptides that activate GPCRs. It is for this reason that NPLP2, NPLP3, and NPLP4 are not considered here. A somewhat similar case can be made for the ITGQGNRIF precursor identified in a similar fashion from the honeybee brain (Hummon et al., 2006). In that case the observed peptide is indeed produced by cleavage at a typical convertase cleavage site and hence may well be produced by peptidergic neurons, but the part of the precursor that is best conserved appears to be a large protein of unknown function. Orthologs of the honey bee ITGQGNRIF precursor are present in both genomes (Supplementary Figure 9), as are orthologs of the honeybee NVPIYQEPRF precursor (Supplementary Figure 10). Interestingly, the *Blattella* homolog of the latter contains a sequence that is almost identical to baratin, a neuropeptide isolated from the brain of the cockroach *Leucophaea maderae* (Nässel et al., 2000). Immunoreactivity to baratin and its *Bombyx* ortholog has been described from neurons and neuroendocrine cells in *Leucophaea* and *Bombyx* (Nässel et al., 2000; Mitsumasu et al., 2009).

### NPF and sNPF

The two species each have two genes coding for an NPF-like peptide. As suggested by a phylogenetic tree based on the predicted NPF sequences, this duplication of the NPF gene has an ancient origin and appears to predate the separation of the Chelicerates from the Mandibulata (Supplementary Figure 11), as was previously suggested (Nuss et al., 2010). Transcriptome data show that the Carabid beetle *Pogonus chalceus* also has these two NPF genes, although we could only find the NPF 1 gene in *Tribolium*. The termite NPF 1 gene is similar to the *Bombyx* NPF1 and the *Daphnia* NPF gene in that it has a second transcript (NPF1b), where an optional exon gets added between the two others (Roller et al., 2008; Dircksen et al., 2011). This optional exon appears to be generally present in insects, because it was found in the NPF 1 genes and/or transcripts from cockroaches, phasmids, beetles, and Lepidoptera. The *Locusta* NPF 1 gene of *Locusta* also has this optional exon (Supplementary Figure 12). The termite has a third NPF gene, which may have become a pseudo gene. Although it has clear sequence similarity to NPF, including the typical intron site in the sequence coding the C-terminal and there are trancriptome reads corresponding to this sequence, it looks like the latter do not form a viable mRNA. A peptide very similar to the predicted structure of *Zootermopsis* NPF 1a has been identified from another termite species and it also has an NPF 1b transcript. Expression data show both transcripts of the NPF 1 gene in the central nervous system and the fore- and mid-gut (Nuss et al., 2010). Recent work showing that *Schistocerca* NPF is important in the regulation of feeding and male reproduction (Van Wielendaele et al., 2013a,b,c) concerns the ortholog of *Locusta* NPF1a.

Both the termite and the locust have one gene coding for sNPF, which in the locust codes for two paracopies and in the termite for one. The *Locusta* transcript is very similar to the one recently described from *Schistocerca* (Dillen et al., 2014). Although the first identified sNPF from insects was isolated from cockroach midgut (Veenstra and Lambrou, 1995), the sNPF gene is not expressed in the midgut of *Schistocerca* (Dillen et al., 2014), and thus likely neither in *Locusta*. This suggests that sNPF isolated from the cockroach midgut was present in the visceral nervous system, like in *Drosophila* (Veenstra et al., 2008).

### Orcokinins and orcomyotropins

Orcokinins were initially isolated from *Orconectes limosus*, a crayfish (Stangier et al., 1992) and similar peptides have also been identified form cockroaches and locusts (Pascual et al., 2004; Hofer et al., 2005). Orcokinin genes are generally present in insects. A more detailed analysis of this gene in *Rhodnius prolixus* showed its mRNA to be alternatively spliced (Sterkel et al., 2012). The newly discovered mRNA was called the B-trancript and the peptides produced from it the B-orcokinins; similar transcripts are generally present in insects and may be specific for the midgut, at least in *Rhodnius* and *Drosophila* (Sterkel et al., 2012; Veenstra and Ida, 2014). Orcokinin-B is an evolutionary old peptide that can be found in the precursors predicted from TSA from species as diverse as *Glomeris postulata* (Myriapoda, Accession # GAKW01029956), *Calanus finmarchinus* (Maxillopoda, Accession # GAXK01131805) or *Speleonectes cf. tulumensis* (Remipedia, Accession # JL137063.1).

**Figure 12:**
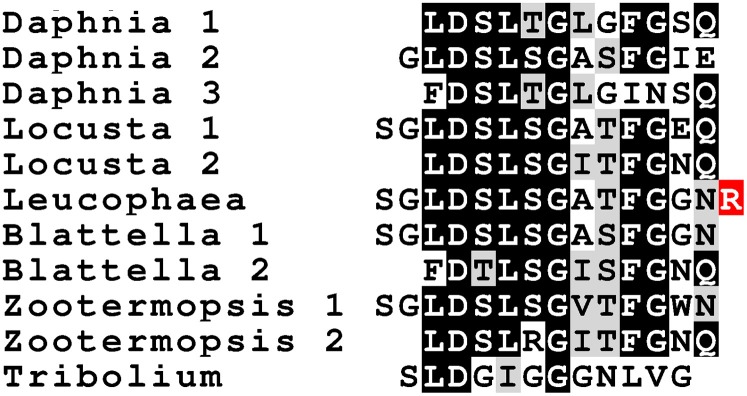
**Sequence of a number of insect orcomyotropins**. Sequences of *Daphnia* and insect orcomyotropins. Notice the strong sequence similarity of the *Leucophaea* peptide identified with a tachykinin immuno assay (Muren and Nässel, 1997) with the predicted *Blattella* orcomyotropin 1. The Arg residue in red can be expected to be removed by a carboxypeptidase.

The orcomyotropins were similarly discovered from *Orconectes limosus* (Dircksen et al., 2000). Sequencing of the genome of *Daphnia pulex* revealed that the orcokinins and the orcomyotropins are encoded by the same gene and this led to the discovery that orcomyotropin-like peptides are also encoded by at least some insect orcokinin genes (Dircksen et al., 2011). This was also found in *Locusta* and *Zootermopsis*, which have both an orcokinin gene encoding both types of orcokinins well as orcomyotropin-like peptides. Interestingly, a peptide isolated using a tachykinin antiserum from the cockroach *Leucophaea maderae* is the only insect orcomyotropin physically identified from insects (Muren and Nässel, 1997); as it has a C-terminal Arg residue it may represent an incompletely processed peptide (Figure 12). Some of the peptides predicted from orcokinin genes, such as the *Locusta* peptides NLDGLGGGHLLRQT and SGLDSLSGATFGEQ predicted from the orcokinin A transcript or SLDGIGGGNLVG from *Tribolium*, share sequence similarity with both the orcomyotropins and the orcokinin B's.

In both the locust and the termite, two different orcokinin transcripts were found, with the B-transcripts encoding large numbers of orcokinin B's and the A-transcripts encoding smaller numbers of orcomyotropins and orcokinin A's.

### Salivary gland salivation stimulating peptide

**Figure 13:**
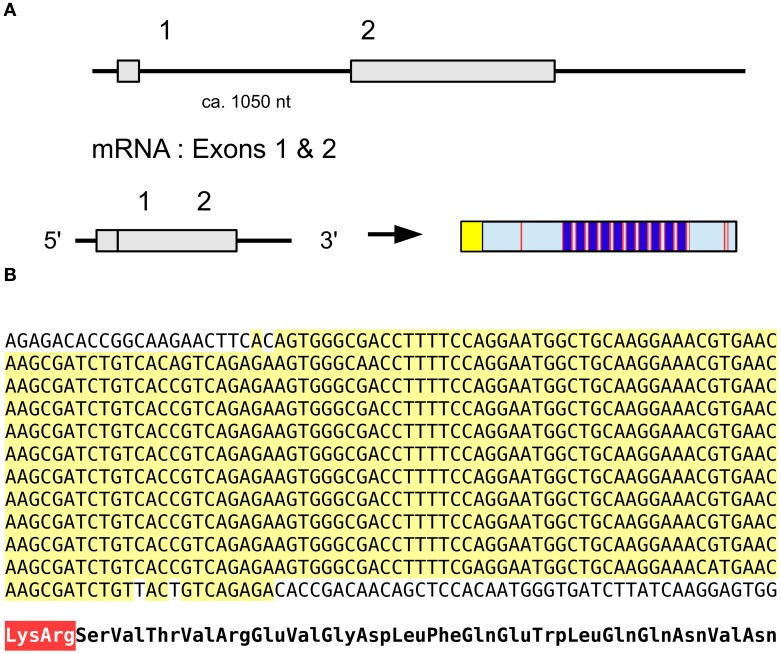
**Salivary gland salivation stimulating peptide. (A)** Organization of the gene, the mRNA consists of two coding exons leading to prepropeptide of which the N-terminus is a typical signal peptide (yellow) followed by the propeptide that contains about 12 copies of the mature peptide (dark blue) separated by convertase cleavage sites (red); the exact number could be smaller or larger as explained in the text. **(B)** Nucleotide sequence of the part of the precursor coding for multiple copies of the active peptide. Note that there is not a single nucleotide substitution in the sequences coding for the various copies, suggesting a recent origin for the amplification. At the bottom the translation of the repeat sequence in amino acids, the Lys-Arg convertase cleavage site is in red.

The salivary gland salivation stimulating peptide (SGSSP) was identified from the salivary glands of *Locusta* by its ability to stimulate the production of cAMP in the same tissue and its sequence reported to be EVGDLFKEWLQGNMN (Veelaert et al., 1995). Its likely precursor contains a number of identical copies of almost the same peptide (Figure 13); the 7th residue in the Edman degradation may have been incorrectly assigned as Lys while it should have been Gln, while in the majority of copies encoded by the precursor the penultimate amino acid residue is Val rather than Met. The predicted precursor shown here is what we believe the best consensus sequence based on the transcriptome and genome data (Figure 13). This is the type of repetitive DNA sequence that is hard if not impossible to resolve with the short paired-end sequences as used for the *Locusta* genome. It is therefore not impossible that a duplication present in the genome assembly that we removed, is indeed real and/or that the actual number of copies of this peptide encoded on this precursor is either larger or smaller. It is worth noting that not only the deduced amino acid sequences for the various paracopies are identical, but so are the DNA sequences encoding them. This suggests a very recent origin for the multiplication of the number of paracopies encoded by this gene. This is an unusual peptide as homologs of this peptide have never been found in other species and even with the large amounts of genomic and transcriptomic data available today we were unable to find a homolog in other species.

### SIFamide and SMYamide

**Figure 14:**
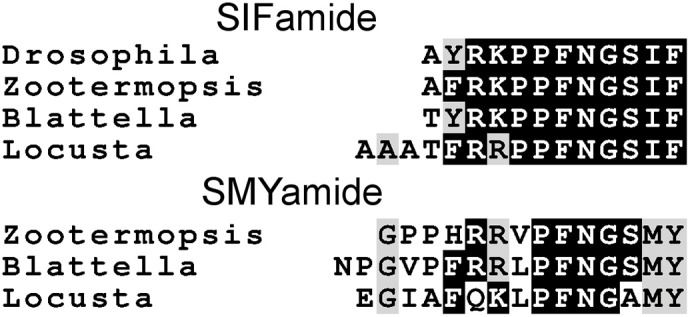
**Comparison of the SIFamide and SMYamide sequences**. All peptide have C-terminal amides. Note that although the SIFamide sequence is well-conserved between *Drosophila* and *Locusta* the C-terminus of SMYamide, although similar to SIFamide, is significantly different.

SIFamide is a peptide initially identified from flies by using a bioassay on *Locusta* (Janssen et al., 1996) and it is now known to be present in many different arthropods. In both the termite and the locust there is a second gene encoding a SIFamide-like peptide, which we have called SMYamide to stress its similarity to SIFamide, even though the predicted C-terminal in *Locusta* is actually AMYamide (Figure 14). An SMYamide gene is also present in the preliminary assembly of the cockroach *Blattella germanica*, but was not found in several other arthropod genomes that are currently assembled. An independent duplication of the SIFamide gene was previously reported for the silkworm, where the gene has been called IMFamide (Roller et al., 2008). A comparison of the genomic sequences shows that the duplication in Lepidoptera is independent from that in cockroaches, locusts, and termites. The expression of the SMYamide gene appears to about 10 percent of that of the SIFamide genes (2 vs. 17 transcriptome reads in *Locusta* and 447 vs. 5210 in *Zootermopsis*).

### Sulfakinin

Although a *Locusta* sulfakinin has been identified (Schoofs et al., 1990d) the part of the gene coding this neuropeptide is not in the current genome assembly. However, a single transcriptome read was found to encode it and part of the sulfakinin gene could then be reconstructed using the genomic reads. It shows that the gene is similar to other insect sulfakinin genes in encoding two different sulfakinins, but the second copy does not adhere to the C-terminal consensus sequence, as the Met residue has been replaced by a Phe residue.

### Vasopressin-like peptides

**Figure 15:**
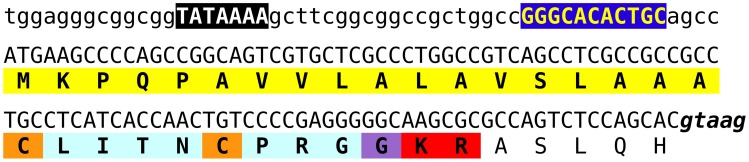
**Nucleotide sequence of the putative promoter and first coding exon of various *Locusta* vasopressin genes**. The TATA box and a near perfect copy of the first motif of the *Drosophila* core promoter (Ohler, 2006) are emphasized, while the putative intron donor splice site is in bold italics. The conceptually translated protein is indicated below the DNA sequence and the predicted signal peptide is highlighted in yellow, the convertase cleavage site in red, the glycine residue transformed into the C-terminal amide in purple, the vasopressin-like sequence in light blue and the two cysteine residues that will form the disulfide bridge in orange.

In the *Zootermopsis* genome a single vasopressin gene was encountered, but the *Locusta* genome seems to have 6–8 such genes. This estimate is based on the number of genomic reads that encode the CLINTCPRGGKR sequence present in various *Locusta* genomic SRAs. Interestingly the first exon of the *Locusta* gene as well as the putative promoter sequence upstream of it, including a TATA box and a near perfect match of motif 1 of the *Drosophila* core promotor described by Ohler (2006), are very well-conserved (Figure 15). Three sequences in the genome assembly are completely identical to this consensus sequence. Of the other eight highly similar sequences in the assembly we detected one that has a single silent nucleotide substitution, another that lacks the last G in the GTAAG splice donor site, and a third one that has several nucleotide substitutions, some of which are predicted to lead to a different, but just as functional, signal peptide. In the latter sequence the amino acid immediately after the Lys-Arg convertase cleavage site is predicted to be a Asp residue rather than the Ala in the other sequences. Finally, there are four incomplete sequences and one that misses a piece in the DNA coding the prepropeptide and cannot produce a vasopressin-like peptide. Whereas the first exon and the DNA sequence immediately preceding it, are very well-conserved between the different genes, this is not the case for the other exons or the intron following the first coding exon. Although it is possible to identify some putative second exons, we were unable to identify the third. Those exons seem to be much less conserved than the first one. It is not clear whether the current genome assembly of the various vasopressin genes is correct, as the short length of the reads makes it difficult to assemble repetitive sequences or multiple copies of a similar gene.

## Discussion

Both the locust and the termite have a virtually complete set of insect neuropeptide genes, the only ones that seem to be lacking are a dilp 7 homolog in *Locusta* and dilp 8 homologs in both species. Nevertheless, as explained below we think the dilp 8 homologs may actually be present but escaped detection. However, this does not necessarily mean that these two species have all insect neuropeptides. For example, in *Nilaparvata* a GPCR was found that is clearly related to the vertebrate TRH receptors (Tanaka et al., 2014), but such a GPCR was not found in the *Zootermopis* genome, and hence it may be that the termite does not have a gene coding its ligand, although it is also possible that this particular GPCR is encoded by the small part of the genomic DNA that is lacking from the genome assembly. These two species not only have what seems to be an almost complete set of neuropeptide genes, but they also appear to use these genes more intensively (two different CNMa transcripts in termites, two myosuppressin transcripts in the locust) and have some extra neuropeptide genes, the tryptopyrokinin and SMYamide genes. Furthermore, on the phylogenetic GPCR trees, the termite has more often than other species two receptors on each leaf. Some of these genes merit special discussion.

### Calcitonin

The presence of calcitonin genes in insects has not been previously reported. The calcitonin-like insect diuretic hormone that activates a calcitonin-like GPCR was generally assumed to be the insect calcitonin ortholog. We here show that insects not only have an authentic calcitonin gene, but also that in the more basal insect groups it produces two different types of calcitonin-like peptides. We were also able to identify candidate receptors for the different calcitonins. Although definitive proof that these GPCRs function as calcitonin receptors is lacking, the evidence is compelling. Like calcitonin B, several of the its putative receptors are expressed in the midgut, as shown both by ESTs and TSAs data as well as the GPCR expression data from the silk worm (Yamanaka et al., 2008). This peptide thus seems to be an important midgut peptide and it is plausible that calcitonin A precursors, which yield only a single peptide per prepropeptide, is made predominantly in the central nervous system.

Processing of the insect calcitonin-B precursors seems different from that of other insect neuropeptide precursors, including the calcitonin-A precursor. Although this peptide has so far only been identified *in silico*, given its well-conserved structure and that of its precursors, it must be assumed that these precursors are cleaved by proteases at Lys-Arg base pairs. Common neuropeptide convertases, however, don't usually cleave next to the cysteine residue of a disulfide bridge (Devi, 1991; Rholam et al., 1995; Veenstra, 2000). As suggested by the structures of the precursors of calcitonin-like diuretic hormones that, like calcitonins, have a C-terminal Pro-amide, common neuropeptide convertases also seem to have difficulty cleaving immediately behind the dipeptide Pro-Gly [all the arthropod precursors of the calcitonin-like diuretic hormone have several dibasic amino acid residues following the Pro-Gly in the precursor, just as found here for the *Locusta* and *Zootermposis* DH31 precursors (Supplementary Figures 1, 2)]. Yet, in the insect calcitonin B precursors the common convertase cleavage site is very close to a Cys residue and the Pro-Gly is followed by a single Lys-Arg pair. Indeed, in some species (*Tribolium, Bombyx*) the cleavage seems to happen at the sequence Pro-Gly-Lys-Arg-Cys, i.e., a convertase site that is both immediately after a Pro-Gly sequence *and* next to a Cys residue. This implies processing by a different convertase.

### Insulin- and relaxin-like peptides

Insects have often multiple insulin genes. In *Drosophila* there are eight, in the silkworm almost fifty (Mizoguchi and Okamoto, 2013). The migratory locust appears to be an exception with only one insulin gene found, but the termite has six such genes. The structural variability of the peptides predicted from these genes is large, yet many species have only a single classical insulin tyrosine kinase receptor. It is thus obvious that several of these peptides must act on the same receptor. Curiously, while in the locust only a single insulin gene was identified, three tyrosine kinase receptors were reported from its genome (Wang et al., 2014).

The *Drosophila* genes CG34411 and CG31096 code for GPCRs predicted to have relaxin-like ligands. Neither of these receptors is activated by *drosophila* dilp 2 (Van Hiel et al., 2014). Dilp 7 has a structure that is much better preserved than the other insulin-like peptides and has unambiguous homologs in mollusks and even some deuterostomes, while genes encoding such a peptide are only present in those genomes that also have a homolog of the *Drosophila* gene CG34411. This suggests that dilp 7 is the ligand for this receptor (Veenstra et al., 2012) and it thus seems appropriate to call it a relaxin-like peptide. The ligand for GC31096 may well be the recently identified dilp 8 (Colombani et al., 2012; Garelli et al., 2012), as it not only has a relaxin-like structure, but is also expressed in the ovary (Chintapalli et al., 2007). As the structure of dilp 8 is poorly conserved it is very difficult to identify in insect genomes. Nevertheless, as termites have its putative receptor (Supplementary Figure 3), we think it is plausible it also has a dilp 8 homolog. We did not find a dilp 7 homolog in the *Locusta* genome, nor evidence for its putative receptor, but there are *Locusta* genomic contigs of a GPCR gene that is an ortholog of *Drosophila* GC31096 and we thus think the migratory locust may well have a dilp 8 ortholog.

The dilp 7 and 8 homologs should perhaps be called relaxin-like peptides, as this reflects better their relationship to the vertebrate hormones. The other insulin-like peptides on the other hand are functionally similar to the insulin-like growth factors (Mizoguchi and Okamoto, 2013). Interestingly, in many species, including the migratory locust, such peptides are also expressed in the fat body (Lagueux et al., 1990; Okamoto et al., 2009a,b), where, like the vertebrate ILGFs there is no convertase to remove the C-peptide. Although the absence of removal of the C-peptide has only been confirmed biochemically in the silk worm (Okamoto et al., 2009a), it seems likely that the same is true for other insect species (Mizoguchi and Okamoto, 2013). In those species that have multiple genes encoding insulin-like peptides, only one of them is expressed in the fat body. Although in *Drosophila* the insulin-like peptide expressed in the fat body is not expressed in endocrine cells, other genes, like dilp 3 and dilp 5 are expressed in both peripheral tissues and endocrine cells (Brogiolo et al., 2001; Chintapalli et al., 2007; Veenstra et al., 2008). It has been shown that an increase in the expression of dilp 3 by midgut muscle is associated with an increase in gut size (O'Brien et al., 2011), suggesting that independently of whether the insulin-like peptide is produced by endocrine cells in the brain or peripheral cells it activates the same receptor, even as its structure is likely different from that of the insulin-like peptides made by neuroendocrine cells.

### Neuroparsin and neoneuroparsins

The data on the expression of the *Locusta* neuroparsin gene are truly intriguing. The number of neuroparsin transcripts seems to be similar to the number of transcripts of other neuropeptides. For example, there are 79 transcriptome reads for the periviscerokinin gene, a gene that appears well-expressed in the ventral nerve cord (Predel and Gäde, 2002) and 154 reads for the myosuppressin gene. Although 154 is a higher number than 66 neuroparsin specific reads, this is due to the much longer nucleotide sequence used to probe the SRAs (279 nucleotides for the myosuppresin gene, 368 for the periviscerokinin gene but only 144 for exon 7 of the neuroparsin gene). Thus, it is the number of neoneuroparsin transcripts that is extremely high, while the number of neuroparsin transcripts seems normal. This means that it is unlikely that the neoneuroparsins are produced by classical neuroendocrine cells in the nervous system, of which the total numbers are perhaps 10–20 times those of the neuroparsin cells. This implies that neoneuroparsins are expressed by a large number of neurons, perhaps all.

Transcripts from the neuroparsin gene are also produced by the fatbody as well as ovary and testes, both in *Locusta* and *Schistocerca* (Lagueux et al., 1992; Claeys et al., 2003, 2005, 2006b). From the *Locusta* data it is impossible to ascertain whether or not the fat body produces only neoneuroparsins, or whether it also makes neuroparsin, but the data from *Schistocerca* suggest that although neuroparsin is expressed in the fat body, neoneuroparsin expression levels are about 500-fold higher (Claeys et al., 2005). As maturation of these proteins does not depend on specific processing enzymes typically present in neuroendocrine cells, they are expected to be secreted efficiently through the constitutive pathway. The question is what are these neoneuroparsins doing and why are they made in such large quantities? The four amino acid sequence (Gly-Gly-Pro-Tyr) that is lacking in the neoneuroparsins (Figure 10) can be expected to be important for correct protein folding as both Gly and Pro significantly affect the secondary structure of proteins. This suggests that the structure of the neoneuroparsins is different from that of neuroparsin and this difference may affect their binding to the receptor. It is difficult to understand why the neoneuroparsins would bind better to this receptor than neuroparsin. On the other hand, why would evolution favor the appearance of molecules that bind less efficiently to the receptor if the insect then needs to make these molecules in large quantities? After all, transcriptome estimates suggest that as many as 1 out of 2500 proteins made by the locust could be a neoneuroparsin. Could it be that the neoneuroparsins do indeed bind to the receptor but are unable to stimulate it and hence act essentially as neuroparsin antagonists? For as unusual as such an explanation seems, it could explain why they are made in such large amounts. In *Locusta* neuroparsin has an anti-juvenile hormone effect (Girardie et al., 1987), while in *Schistocerca* juvenile hormone and ecdysone increase transcripts for the neoneuroparsins, but not for neuroparsin (Claeys et al., 2006a), thus suggesting that the structural difference may be associated with a functional difference between the neoneuroparsins and neuroparsin. Unfortunately, a neuroparsin receptor has not yet been identified and hence the relative binding and stimulating activities of neuroparsin and the neoneuroparsins cannot be determined. The function of neuroparsin remains obscure, but it could be an important differentiation hormone (Veenstra, 2010b), an idea consistent with their effects on neurons in culture (Vanhems et al., 1990). If this were true, it might well have an important role in the differentiation between gregarious and solitary forms as also suggested by their expression in *Schistocerca* (Claeys et al., 2006a). Although the metabolic cost of producing so many neoneuroparsins seems high, the value of polymorphism for species survival is clear.

### Tryptopyrokinins and salivation

If the existence of specific tryptopyrokinin genes was not unexpected, the question still arises as to why should the locust need four such genes? In *Drosophila* and *Bombyx*, the tryptopyrokinin receptor is strongly expressed in the salivary gland (Chintapalli et al., 2007; Yamanaka et al., 2008). Although this is not the case in *Rhodnius* (Paluzzi and O'Donnell, 2012), it seems nevertheless possible that in locusts tryptopyrokinins are important regulators of salivation. Apart from four tryptopyrokinin genes the locust also has evolved what looks like an entirely new neuropeptide gene (see below) encoding mulitple copies of a neuropeptide that increases salivation by means of the stimulation of intracellular cAMP (Veelaert et al., 1995). The tryptopyrokinins usually are released as hormones into the hemolymph, but the salivation peptide is present in the salivary glands and can thus be expected to be released directly in the gland. It thus appears that the locust may have five neuropeptide genes controlling salivation in addition to salivation control by aminergic motoneurons in the suboesophageal ganglion and perhaps still other neuropeptides (see e.g., Ali et al., 1993). Even if our suggestion that the tryptopyrokinins may regulate salivation in the migratory locust were to be incorrect, the salivation peptide still raises the question as to why this locust needs to reinforce control of its salivary glands by what appears to be a novel neuropeptide. We believe the answer may be that this is not an ordinary but a migratory locust. Once millions of locusts descend on a field to feed, they all have great interest in starting to feed fast and furiously; for if not, there will be nothing left. The physiological changes from flying to feeding full speed are dramatic, but if the insect does not manage to make the transition fast, it will starve. Perhaps this explains why this species has four tryptopyrokinin genes and still evolved an entirely new salivation neuropeptide.

### Vasopressin

It is unfortunate that we were unable to determine the sequence(s) of the last part of the precursor of the vasopressin-like peptide. It is known from humans that numerous mutations of the neurophysin part of the vasopressin precursor will lead to autosomal dominant familial neurohypophyseal diabetes insipidus, as due to the incorrect folding of the vasopressin precursor in the endoplasmatic reticulum the cells producing vasopressin die (Christensen et al., 2004). Obviously, human life span is much larger than that of a locust, and if neurons only die after a year, the locust would be dead anyway, but it still raises interesting questions with regard to the synthesis of this neuropeptide. When the vasopressin-like peptide was isolated from *Locusta*, it was followed by both RIA and a bioassay that measures an increase in amaranth secretion by the Malphigian tubules (Proux et al., 1987). However, when the identified peptide was synthesized in its monomeric form, i.e., in the same form as vasopressin, it had no biological activity. It was only active, when it was synthesized as an anti-parallel dimer. This was very surprising as all vasopressin-related peptides are known as monomers and a dimer had never been found. Proteins and peptides containing disulfide bridges need a mechanism to avoid the formation of inappropriate disulfide bridges like those occurring in vasopressin dimers. It seems a reasonable hypothesis, that neurophysins may help the proper folding of the vasopressin precursor and once they are no longer made correctly, “errors” in the form of dimers may occur. As will be discussed below, the cells making the vasopressin-like peptide in *Locusta* need to make it in large quantities. One way to speed up production is to condense the gene; the short distance between the TATA box and the transcription start site of the various vasopressin genes (Figure 15) suggests that this has occurred. Another way would be to get rid of the neurophysin part of the precursor, which might then facilitate the formation of dimers. It is for this reason that it will be very interesting to see the complete sequence(s) of this precursor.

The function of this peptide in insects is poorly understood. Although it was initially proposed that the peptide had a diuretic function (Proux et al., 1987), it turned out that a vasopressin-like peptide from *Locusta* in any conformation (monomer, parallel dimer or anti-parallel dimer) failed to stimulate fluid secretion by the Malphigian tubules (Coast et al., 1993). The current hypothesis is that the peptide may act indirectly through the release of a brain diuretic hormone (Aikins et al., 2008). This peptide has received less attention than other insect neuropeptides because it is absent from *Drosophila*. Consequently, we still know very little about its physiology. In the pond snail the orthologous peptide acts on the *vas deferens* (Van Kesteren et al., 1995), while in *Schistocerca* there is sexual dimorphism of the axons in the terminal ganglion (Tyrer et al., 1993). Others have shown that ovariectomy of female crickets leads to a decrease in neurosecretory material in these neurons that is associated with changes at cellular level suggesting an increase in peptide production (Dürnberger et al., 1978). Together these data may indicate a reproductive function for this peptide.

### Amplification of neuropeptide genes

Some neuropeptide genes are amplified, while others are not. In the locust, the insulin gene is not amplified, whereas it is amplified in many other insect species. On the other hand, the *Locusta* vasopressin gene is amplified, while in other species it is not. Thus, it seems an appropriate occasion to discuss this phenomenon. It is interesting to note that often the same genes are amplified in different species. In insects, these are usually the AKH and insulin genes. The latter shows a high degree of amplification. There are six genes in *Drosophila*, many more in the silk worm (e.g., Mizoguchi and Okamoto, 2013) and five in the termite genome. However, whereas in *Bombyx* and *Drosophila* numerous insulin genes are expressed by a limited number of brain neuroendocrine cells, only a single insulin gene is expressed in the fat body of both species. We think this is a clue as to why the insulin gene is amplified. Whereas the fat body consists of many cells and a small amount of insulin made by each of these cells will suffice to attain a physiologically relevant hemolymph concentration of this peptide, this is a much more difficult task to achieve by a limited number of neuroendocrine cells in the brain. It seems that two copies of the insulin gene (one on each chromosome) is simply insufficient to keep up with the large amounts of mRNA needed to produce the quantities of hormone that need to be secreted. When it is a matter of a small neuropeptide, the number of paracopies on the gene can be amplified. For example, leucokinin appears to be a diuretic peptide that in *Drosophila* is produced from a gene encoding a single copy (Terhzaz et al., 1999), while mosquitoes that after a blood meal increase diuresis, make three copies from the same gene (Veenstra et al., 1997). It is obviously more difficult to produce multiple copies of a larger neuropeptide, probably even more so when it contains several disulfide bridges that need to be formed correctly on entry of its precursor into the endoplasmatic reticulum. The hypothesis that it is a matter of quantity that needs to be produced is strongly reinforced by the observation that the *Locusta* vasopressin gene is amplified. In other insect genomes only a single copy of this gene has been found, as is the case for the termite as well as several other insects for which preliminary genome assemblies are available. So why is vasopressin so different in *Locusta*? In *Locusta* the neurons producing this peptide seem to have acquired an endocrine function with the peptide being released as a hormone from neurohemal terminals located on the thoracic peripheral nerves. In other locusts, such as *Schistocerca gregaria*, these cells are not only much smaller but they neither have these neuroendocrine release sites (Tyrer et al., 1993). Interestingly, in some crickets these neurons appear to have independently acquired a neuroendocrine function; in those animals the neurohemal release site is located on the ventral side of the brain (Weinbörmair et al., 1975). It is thus clear that in *Locusta* the amount of peptide that needs to be made to achieve physiological effects is much larger than in *Schistocerca*, because in *Locusta* the peptide is released as a hormone into the hemolymph whereas in *Schistocerca* the peptide is released only within the central nervous system. Thus, the correlation between quantity of peptide released and number of genes holds very well and it is hard to escape the notion that this is the reason why this particular gene got amplified. We would expect that crickets too may well have more than one vasopressin gene, whereas *Schistocerca* should need only one. Another solution to the problem would have been to increase the number of neurons producing this peptide, but it is probably much more difficult for a spontaneous mutation to alter the developmental program of neurogenesis in such a precise fashion than to duplicate a gene. Indeed, the number of endocrine cells in the corpora cardiaca producing AKH seems much more variable, from hundreds in locusts and cockroaches to about 20 in *Drosophila*.

### SIFamide and SMYamide

Both the locust and termite have a second gene that codes for a SIFamide related peptide. The same phenomenon has been described previously for *Bombyx mori* (Roller et al., 2008) and may well be generally occurring in Lepidoptera as such a gene is also present in the genomes of several other Lepidoptera. The SIFamide neurons are very large and their axons innervate many regions of the neuropile in insect species as diverse as *Drosophila* and *Schistocerca* (Terhzaz et al., 2007; Gellerer et al., 2014). Thus, these neurons likely produce and release large quantities of neuropeptides. However, as they release their peptide exclusively within the nervous system, a single gene could be sufficient, as is the case for example for the *Schistocerca* vasopressin neurons. Furthermore, whereas the primary sequence of the active peptide predicted from the various *Locusta* vasopressin genes is perfectly conserved, that is not the case for the SIFamide offshoot. Since the C-terminal of SIFamide has been very well-conserved during evolution, this suggests that neither SMYamide nor IMFamide has the same affinity for the SIFamide receptor as SIFamide itself. Intriguingly, both *Locusta* and *Zootermopsis* not only have a GPCR that is likely the SIFamide receptor, but also a second GPCR that seems to be closely related to this putative SIFamide receptor (*Zootermopsis* A11 in Supplementary Figure 3), raising the question whether that receptor might be more specific for the predicted SMYamides. However, this second receptor also has a homolog in *Nilaparvata* (Tanaka et al., 2014; see Supplementary Figure 3, Nilaparvata_A11), but SMYamide has so far not been found in this species. We hypothesize that the duplication of the SIFamide is not maintained because of a need to produce more peptide, but rather to produce a different one.

In *Drosophila* SIFamide is produced by four very large interneurons that appear important for behavior. Sexual behavior in males is notably perturbed in flies that lack these neurons or which production of the peptide has been abolished by selective expression of SIFamide specific RNAi (Terhzaz et al., 2007). In *Bombyx mori* (Roller et al., 2008), the SIFamide-related peptide IMFamide is expressed not only in neurons homologous to the *Drosophila* SIFamide neurons, but also in a more ventrally located bilateral pair of neurons (Roller et al., 2008). Recent work on *Schistocerca* shows similar SIFamide immunoreactive neurons in this species (Gellerer et al., 2014). Given the strong similarity between SIFamide and SMYamide, it is likely that SIFamide antisera cross-react with SMYamide and it will be interesting to see which, if any, of the SIFamide immunoreactive neurons produce SMYamide in the locust.

### *De novo* evolution of neuropeptides

As indicated in the introduction, most neuropeptides and their receptors are evolutionarily very old and many have homologs in both deuterostomes and protostomes. The sequence of a neuropeptide GPCR is generally sufficient to predict its likely ligand due to the coevolution between receptors and ligands. This easily leads to the generalization that neuropeptides and receptors *always* coevolve. Indeed, there are very few examples of instances where one can make the argument that a neuropeptide evolved *de novo*.

The locust genome contains three genes that code unusual biologically active peptides, ovary maturating peptide, accessory gland myotropin II and SGSSP, the salivation peptide. None of these peptides have been found in any other insect orders and, with the exception of ovary maturating parsin, which is also made by another migratory locust, *Schistocerca gregaria*, not even in other insect species. This suggests a recent origin of these peptides. The accessory gland myotropin II was isolated from the male accessory gland. When males copulate with a female they not only transfer sperm, but also material made by the accessory glands, containing many substances that change female physiology. In particular they may induce ovulation and make the female reluctant to mate again. It is known that the accessory gland proteins evolve very rapidly (e.g., Wolfner, 2002; Panhuis et al., 2006), and it is therefore less surprising that this particular peptide has not been found in other species. Nothing is known about its mode of action and as it is derived from a protein that lacks the typical neuropeptide convertase cleavage sites it is unlikely to be a classical neuropeptide, although it might act through a neuropeptide GPCR.

Ovary maturating parsin on the other hand is a neuropeptide. It was isolated from the corpora cardiaca and brain of the migratory locust and it has impressive effects on reproductive physiology of *Locusta* (Girardie and Girardie, 1996; Girardie et al., 1998). In *Locusta* it is produced from the same gene that produces the CRF-like diuretic hormone and the same is true for *Schistocerca* (Van Wielendaele et al., 2012). No clear homologs of this peptide have been identified in other insect species and immunoreactivity with antiserum against this hormone is only detected in locusts (Richard et al., 1994). However, it cannot be excluded that it is the function rather than the structure of this peptide that has been conserved and it may yet be determined that parts of the CRF-like diuretic hormone precursor in other insect species have similar effects. Nevertheless, experiments will be needed to address the apparent contradiction between the results of injections of this peptide and eliminating its production by RNAi silencing.

The most interesting *Locusta* peptide with regard to neuropeptide evolution is the salivation peptide. It looks like a real neuropeptide, as its precursor has the typical convertase cleavage sites and it stimulates the production of cyclic AMP, which usually occurs after interaction with a GPCR. On the other hand, it looks like this peptide has effectively evolved *de novo*, as similar peptides have not been found elsewhere and at the very least the amplification of the number of paracopies of the neuropeptide must be of a very recent origin as nucleotide mutations in the sequences coding them have yet to appear. Although it remains to be proven that this peptide acts on a GPCR, this seems the most likely explanation for an increase in intracellular cyclic AMP. It is hard to imagine that it would not act on a preexisting GPCR. Identification of the mechanism of action of this peptide seems thus of much interest.

There are other examples of what appear to be *de novo* evolution of ligands for existing neuropeptide receptors. On the one hand there are the examples of the tachykinins in salivary glands of the mosquito *Aedes aegypti* (Champagne and Ribeiro, 1994) and on the other hand there are the male accessory gland peptides in various insects that act on neuropeptide receptors in the female. The famous *Drosophila* sex peptide (Kubli, 2003) and the so called head peptide from the mosquito *Aedes aegypti* (Brown et al., 1994; Naccarati et al., 2012) both act through neuropeptide receptors, those for allatostatin B and short NPF, respectively (Kim et al., 2010; Poels et al., 2010; Yamanaka et al., 2010; Liesch et al., 2013). While the origin for both of them is obscure, in the case of the mosquito the DNA sequences (Stracker et al., 2002) suggest not only that this peptide has a recent origin, as it is lacking in other mosquitoes, but also that its origin is independent from the sNPF neuropeptide gene. Such “accidental” evolution of new ligand receptor combinations may be more common than generally realized. We will only find it when it has happened in a clade that has become a success during evolution. A possible example of such a successful *de novo* association between a ligand and a receptor is provided by prostaglandin D and the DP2 receptor (Hirai et al., 2001).

## Conclusion

If there is a message from these genomes, it is probably that we will need more physiology. Genome analysis mostly identifies neuropeptide genes that we know already. Of course, there are interesting details to discover, such as the alternative splicing of the myosuppressin gene in *Locusta* or the CNMa gene in *Zootermopsis*, or the calcitonin gene and the putative identification of its receptors. The most interesting findings of the *Locusta* genome concern the salivation peptide, vasopressin, neuroparsins, and the tryptopyrokinins, but these findings pose more questions than can be solved by bioinformatics alone. Real physiology will be needed to answer these questions. The importance of physiology is nicely illustrated by the salivation peptide. If this peptide had not been previously shown to stimulate salivation we would not have a clue as to its function and, consequently, it would hardly be perceived as interesting. The physiological significance of the neuroparsins, tryptopyrokinins, and the vasopressin-like peptide similarly awaits physiological experiments.

### Conflict of interest statement

The author declares that the research was conducted in the absence of any commercial or financial relationships that could be construed as a potential conflict of interest.
