# Supplementary material for: The contribution of the genomes of a termite and a locust to our understanding of insect neuropeptides and neurohormones
Source: Front Physiol. 2014 Nov 19;5:454. doi: 10.3389/fphys.2014.00454 (PMC4237046; doi:10.3389/fphys.2014.00454)
Supplement: Supplementary file 2 [file Presentation1.PDF]

Supplementary Material

The contributions of the genomes of a termite and a locust to our understanding of insect neuropeptides

Jan A. Veenstra[1]*

[1]INCIA UMR 5287 CNRS, Université de Bordeaux, Pessac,

*** Correspondence:** J.A.Veenstra, INCIA UMR 5287 CNRS, Université de Bordeaux, allée Geoffroy St Hillaire, CS 50023, 33 615 Pessac Cedex, France.
jan.veenstra@u-bordeaux.fr

Supplementary Figure 1. *Zootermopsis* neuropeptide and neurohormones precursors page **2**
Supplementary Figure 2. *Locusta* neuropeptide and neurohormone precursors page **6**
Supplementary Figure 3. Phylogenetic tree of GPCRAs page **11**
Supplementary Figure 4. Phylogenetic tree of GPCRBs page **19**
Supplementary Figure 5. Calcitonin precursors page **21**
Supplementary Figure 6. Phylogenetic tree of calcitonin A and B peptides page **22**
Supplementary Figure 7. Phylogenetic tree of eclosion hormone page **23**
Supplementary Figure 8. Elevenin gene strucures page **24**
Supplementary Figure 9. ITGN sequences page **25**
Supplementary Figure 10. Alignmnet of putative baratin precursors page **26**
Supplementary Figure 11. Phylogenetic tree of NPF page **27**
Supplementary Figure 12. Alignment of NPF1 precursors page **28**

Supplementary Figures 1 and 2. Amino acid residues in yellow indicate the signal peptide or, in the case of the *Zootermopsis* allatostatin CC precursor, the signal anchor. Red is used to indicate likely convertase cleavage sites and those amino acid residues that are predicted to be removed by carboxypeptidase, either after convertase cleavage or because they are located at the C-terminal of the precursor. Glycine residues in mauve are predicted to transformed into C-terminal amides in the mature peptides. Interrogation marks between parentheses in the *Locusta* sequenece indicate that the sequence could not be extended beyond what is shown. Light blue is used to delineate likely biologically active peptides, grey for possibly biologically active peptides.

Supplementary Figures 6, 7 and 10. Phylogenetic trees of short peptide sequences as produced here in supplementary figures 6, 7 and 10 should be intrepretated with caution. The objective of making these trees is to check whether or not there is evidence for an early origin of different copies of the same or a very similar hormone and not a reliable phylogenetic tree.

Supplementary Figure 1. Predicted *Zootermopsis* neuropeptide and neurohormone precursors

Zootermopsis Adipokinetic hormone
MSCIAKTIFVMVALIFVFCEAQVNFTPNWGKRSGLQDAPCKASTEAAMYIYKLIQNEAQKLLDCEKFGSN

Zootermopsis AKH/Corazonin-related peptide
MTSRRLCGRALLLVAVLNCLHFRTWGQVTFSRDWNAGKRSPADLQCSAIIKSADEFCRVLIEEFRQLAACETKSLLRFLK
DYDDSQADIFMESQNGRQTPTNDLHQRNF

Zootermopsis Allatostatin A
MLGLQSSLGSLKMTLFSVLLLHLTVLVLGTASAPSETHETAEESSPVSAGGMGLVPQLEDSSSAENAELDFVKRLYDFGL
GKRAYSYVSEYKRLPVYNFGLGKRSKMYSFGLGKRSGTEGRLYSFGLGKRDYDDYAEENEDEDQTNGDEEFEDSDLDLME
KRERLYSFGLGKRARPYSFGLGKRSPSSGIQRLYGFGLGKRGGSLYSFGLGKRADGRLYSFGLGKRPVNSGRQSGSRFNF
GLGKRSDIDYNEFDDELGEEAKGFPQGHRYYLGLGKREVAPSELDAIRNEEREKINYRDESRKNETAEGHHSGERVKRSL
HYAFGLGKRAYDLESSTIDTDEDDEARNDFARLIRRPFNFGLGKRIPLYDFGIGKRSER

Zootermopsis Allatostatin B (mip)
MEYFAIPGVILWLLLLAVSPSSQGDPISDPARVPPGSGPSGGTLSRAQDVPTQVQGPEEDKRGWRDLQGGWGKRGWQDLQ
GGWGKRGWQDLQGGWGKRGWQDLQGGWGKRGWQDLQGGWGKRGWQDLQGGWGKRGWQDLQGGWGKRGWQDLQGGWGKRGW
QDLQGGWGKRGWQDLQGGWGKRGWQDLQGGWGKRGWQDLQGGWGKRGWDKFHGSWGKRDSDLDFDVNSLDNDVADEELVD
EESGEDLKRAWSSLKGGWGKRAADWANFSSWGKRDPGWNNLKGLWGKRADTNWNRLSAAWGKRSIGGETGIKEDPARVMS
SSEE

Zootermopsis Allatostatin C
MSTATATKLMFAMLLAMMTLSCAVGKTLDQPGEKDRLISELDLVDDDGSIETALINYLFAKQVVNRLRSQMDVSDLQRKR
SYWKQCAFNAVSCFGK

Zootermopsis Allatostatin CC
MGQRTLRLHQRRLHANTPHTPCALLHSPPISNASLPFRNTSYLWLFALVFVSCSGTFLASAEGNPVTSPVEQRVFKRSPS
EGEIAVADYPDYQAGVRYDEYPVVVPKRTALLLDKIMVALQKAVEDGKGDRNFASNVAENKMDLQRRGQQKGRVYWRCYF
NAVTCFKRK

Zootermopsis Allatotropin
MRASLSVNCMIAATVLVVLVLCDCVSSGPSYQNARNKPRTIRGFKNVALSTARGFGKRDGALSYLADNANTASEPTLESL
PVEWFVEELRTNPELARIIVHKFVDADQDGELSAEELLRPMY

Zootermopsis Bursicon alpha
MACQQPSIQQIAVSAVLLLSLGYVVLVDAKDECQVTPVIHVLQYPGCVPKPIPSFACTGRCSSYLQVSGSKIWQMERSCM
CCQESGEREANVSLFCPKAKAGERKFRKVSTKAPLECMCRPCSTVEESAVIPQEIAGYADEGPLSNHFRKSL

Zootermopsis Bursicon beta
MVSEVAWWFRSLLLFILFFVAVPSTVQQGEDVACETLPSEIHIIKEEFDDLGRLQRTCSSEVSVNKCEGACTSQVQPSVI
TPTGFLKECYCCRESFLRERIISLTHCYDPDGIRLTQEGQASLDVKIREPADCKCFKCGDFSR

Zootermopsis Calcitonin A transcript
MEWKKEMTLVLYLLVVMATAAWASSKEIAQELMDSHIKSLQENRRTVRLLKSLLEDLDVNMETVQKRTSCLINAGLSHSC
DNRDFIGAVEENKYWRSLDSPGKRRRRGIFNVQ

Calcitonin B transcript
MEWKKEMTLVLYLLVVMATAAWASSKEIAQELMDSDKAAERAKRCVNTGDDSCANGYIPGAGADDDYFSGGNTPGKRATL
DTLNPQELRTAWQKCLNTGDDSCANGYIPGAGADDDYFSGGNTPGKRAALETLTPQQLRRAWLKCLNTGDDSCANGYIPG
AGADDDYFSGGNTPGKRSVSSFDVKRHCLLFPMSPVCRNL

Zootermopsis Calcitonin-like Diuretic hormone
MNSCVVLLTSALLVGAVLMISVVNAVESAPLSSHRNNFISDQDSEPDSEYVLEMLARLGQSIIRANDLENSKRGLDLGLS
RGFSGSQAAKHLMGLAAANYAGGPGRRRRSVDESS

Zootermopsis CCHamide 1
MFSPSLATRTSAVGVAARFAIVLLVFGFAECATGSCLSYGHSCWGAHGKRSGNVAADEAPVDVSEAVAAIAAPEDTRWFL
SKLVRQAEPVSGSKVWKRLRGVRVPSAADRRRNEVQWRNGASEEEDEAEATGGNDAVARSRTIHLEGSEETPAGILVPAG
GEYPAGENQDAEVLLMADDQAIRRTPQKLRVYKLMNHQGGKLD

Zootermopsis CCHamide 2
MVFHRHTSILIVLMAIVILASEIHLSSAKRGCSAFGHSCFGGHGKRTDGNAVLIPGPDSDQQPLLVFRPEEEDADDAMVQ
QGALPSAWSATAGISPRQPPPLPAKYNLTPFLRQWFQALRHSTGDVEAK

Zootermopsis CNMa A-transcript
MVSKMSSRAMILWTIVMVMAFSCRAEAAPEAFIAPADFRELQQIALAENGLQDAKKREEIATLFQLLEQYHNEWQQQQKP
TDEETETQDIGAPQVPEDYSNLPNRMPVETKFFRSQDGVVKDETKRGNYMSLCHFKICNMGRKRNFRWNPWIRR

Zootermopsis CNMa B-transcript
MVSKMSSRAMILWTIVMVMAFSCRAEAAPEAFIAPADFRELQQIALAENGLQDAKKREEIATLFQLLEQYHNEWQQQQKP
TDEETETQDIGAPQVPEDYSNLPNRMPVETKFFRSQDGVVKDETKRGNYPPPLCYFKICNMGRKRNPH

Zootermopsis Corazonin
MHLNSTASSSRCRSRMTGLLLIFCCLTGSILAQTFQYSRGWTNGRKRSGPSPQMLIPSSASGERLFQNTDESSAISNPCS
QLQRIRFLLGARNPQQFYFPCETWTDIFETPSEEVSERFRRKAHQDFAEGNNIEGN

Zootermopsis CRF-like Diuretic hormone
MLAAVLTLLLSALVSCGTASIEPPLLEALAAPSADHETTSYLLPRLSAKFRPHGDWDSAPDPRFYVLTELQRESSQAARR
MKRTGAVPSLSIVNPLDVLRQRLLLEIARRRMRQSQDQIQANREMLQTIGKRDADQSQQRSSDDDDEDDEMDSEFMVSST
DEKSGSNRPRDTPDWSPSSGPRWDDEFASQHH

Zootermopsis Crustacean cardioactive peptide
MQMCHVVIGCSVAVLLMIIGLPLASCDSVIIQKRQIDPADVDRILDPKRKRPFCNAFTGCGKKRSDESMGTLVELNSEPA
VEDLSRKILSETKLWEAIQEARAELLRRRQEQLQEGQYATAVERPIPLSITGYRKKRSVIPEGTGNSLLTTSEPQDQSTK
TWSR

Zootermopsis Ecdysis-triggering hormone
MKKSAKQNWNLIATNCFSFGLPLIMVAAILVVLTACPSIISADETGTNFFLKSSKSVPRIGRRSEYDFLKASKEIPRIGR
RREMSPLTPGRESAMWPWFRTADTIPGPSRRSNYYLHEEGKPLSWTSVEKTMEEAPELWKPDLWRKNSETFPLRDDFDVE
QVVRRSPGRFGSTKEIDEGRNQVEV

Zootermopsis Eclosion hormone 1
MHSLVCLFMFWFSTVISLLAAMEQRKISEAVVLAMSVAFLAATVVVPSGATSYSIGVCIRNCAQCKKMFGPYFEGQLCAD
ACVKFKGKIIPDCEDLASIAPFLNKFE

Zootermopsis Eclosion hormone 2
MTGHGAVFYLAVLIIVSTDDCWAGSNVGVCITNCGQCKQMYGHYFEGQVCVESCLSTNGNLLPDCNNPNTLRGLLKRLY

Zootermopsis Elevenin
MSRGCARLLPLLLSTGMLLYLIAMTEAVPGPLDCRRFVYAPKCRGVAAKRGFLQANRPGYLLDTDRKVDGLEEVLGLYAT
PQPIAVPQQSDSRGQVQGFATRGHSGQAWSAAGDQQGLKTDTFYDWYLSNRKRSRDTDVTYDY

Zootermopsis FMRFamide
MMRVMLALVCVAVAASYPTDTPISEQQNNELATPDDAGALGDDCEFEDVSPPTTKRRQEDEDKPAPVHRHCSSRIYVRLG
RRSTDGSASGLNLPEEEDTALPRLGRGGRPNYNFVRFGRGGKNDNFVRFGRGGKQDNFIRFGRDRSDNFVRLGRTRADNF
IRFGRGKQENFIRFGRDKQGNFIRFGRGMKNSDDNDETFRLEDTDVNPENEFSASSNLRVGRSGKSEGDFIRFGRARPSN
FIRLGRGDDDLRQPKEGGTGRSEIARYGRQTYDDNFVRLGRSPGNNDAMRRGKLTDRNFIRLGRSGPQNDYAREWENSEM
SGRSDNPPTNNNFIRLGKRAKGGDERENFVRPGRDAEQTPGTENKTVKHTRNRRSATFSGGDENPEDSSDCPVVTSNSAS
DDDKIASAFEYSQIPFQYYSPLASGIPNYILGPLGNEDAAKGRKGGGLQKRHYIRLG

Zootermopsis Glycoprotein hormone alpha 2 (GPA2)
MFPVSWRLQCCYLLFIFVTILSVVSRTRARDAWERPGCHKVGHTRKISIPDCVEFHITTNACRGYCESWAVPSAIDTLRV
NPHQAITSVGQCCNIMDTEDVEVQVMCLDGTRDLVFKSAKSCSCYHCKKD

Zootermopsis Glycoprotein hormone beta 5 (GPB5)
MSLVFSRLPFASALCFALVATLWLGAETSSLQESTLASTLECHRRMYAYKVTKTDSAGRVCWDVINVMSCWGRCDSNEIS
DWRFPYKRSFHPVCLHDTRAVSSATLQNCEEGVEPGTEVYEYLEALTCRCMVCKSSEASCEGLRYRGQRSGPFLVGGR

Zootermopsis Insulin-like peptide 1
MWRLYLRLVAIAALCLCTLAQAQSDLFQLGDKRNTNKYCGRNLANMLRYVCNGNYYPMFKKASQDVEDVNDSGIWIQPLP
IEEPQLQYPFHSRSNAATLVPGSLRRHTRGVYDECCRKSCTIQEMVSYCGSR

Zootermopsis Insulin-like peptide 2
MWRACFRIVVVVALCLCSLAQSQSDIFPFPDKRPETKRYCGSNLVDILQLVCNGKYYSNINNSNNYSPHVGRKKSMPEAD
EDFWQQLQPVEEQMKFPFRSRSSVSTFAHRIFKRHTVGVAYECCINKGCTVYELRSYCAP

Zootermopsis Insulin-like peptide 3
MWRLCLRAMLISMICVCALTDNPSTTPKFVEKREAHRYCGRNLANILKFLCQGHYNGIDYDRKRRDVDFYGEPKLYGDHD
YDFWSQAEDVDDFADLHYPFRPRSVSSVLLNKLFRRRPKRNNGGIVEECCIYKGCTLSELTEYCSPVQ

Zootermopsis Insulin-like peptide 4
MRTILLRLTVIGMICSWALPDSQLGVRKRETAYRYCGPNLANILRLICNGSYHTDEDWKRNGGSIQKKQEHDKELDFWTP
PEAAMEQFPFRTWWMANQFSNRVFRRQRRDAGIVEECCVFKGCTISELSEYCAES

Zootermopsis Insulin-like peptide 5
MWRLYLRLVAIAALCLCTLAQAQSDLFQLGDKRNTNKYCGRNLANMLRYVCNGNYYPMFKKASQDVEDVNDSGIWIQPLP
IEEPQLQYPFHSRSNAATLVPGSLRRHTRGVYDECCRKSCTIQEMVSYCGSR

Zootermopsis Ion transport peptide A transcript
MQHPHLTRILACSLLVSMIITSLLTSRTSGLAVGHSLHKRSFFEIQCKGVYDKSIFARLDRICEDCYNLFREPQLHSLCR
KNCFTTDYFKGCLDVLLLQDEMEKYQTWIKQLHGAEPGV

Zootermopsis Ion transport peptide B transcript
MQHPHLTRILACSLLVSMIITSLLTSRTSGLAVGHSLHKRSFFEIQCKGVYDKSIFARLDRICEDCYNLFREPQLHSLCR
SKCFSSDYFKGCLEALLLKEEEQKFNQMVEYVGK

Zootermopsis Leucokinin
MSLAGRKFLVLLTVTIAARGQTPRAFAVETHNLLGSLPALVGQDGLGEGGQADAGLDLDLDRRETERQALADLLLRLPEP
SVQRKRRRRHEDEQRSAGSAPGVSRDAGPYAEDSRLEDSSAGRGVMMPSRLFGRRGKEENESPAFGERGFKRATVGAAGR
RAFGTWGGKRAAEFHSWGGKTSSEEQSALAVVSSKRRRPAFNHWGGKRDSASNALADKRDIFGLSVSDEPSLRTWGSNSA
PPFSEPSPQKDAVLRSLCNEHPAFSILSSNYEPKPALIALGCKRAFASWGGKREARGPPPSAWGSKGSSDGDARDTRSFL
SWGGKRNFIAGGMKEGAEELGKRRFSSWGGKRGTMRTGEIGPGDEGEQSSARISSPEGYRQLLEGFGRKMGLPHEYNEIA
EDPEEIEAGENTQRSEPHEEFQQQLGGQSAITKRSEAKARVGKSLFRPWGGKRDHVLPPPPFSPPALGGFRLLSDLFKEE
GHARSKTPGSREWGPSPAGEVKDRI

Zootermopsis Myosuppressin
MKHVCVVLICFLAALLAFSPLRVSAVPPPQCSPSVRKVCAALSTFYELSNAMEAYLDDKVARDSSNPLVDAGVKRQDVDH
VFLRFGRRR

Zootermopsis Natalisin
MTSTLLMVSLVLTWSCSDVVRSSSELRNMQSDAKNINAVETDTGRDIERRVTRSDVRATLGNKPEPGFWPARGRRSYFED
VPPLFWSDAPLRPNSQTPRMHWRSAAPHKPRGRLQLHTPLFAEEPNYVLLDRRDEPNVSPDGTNDDDPFWVARGRRRYEA
RTASGEGSLWAAKVRPTSISGLRELMYADEPFWAERIRKSDHDSLESAEDPFWPTRGKRNIFRLPEGTPRRRVLKSLSGN
ELCWAARGRRSADEDSRDKRGLLESLSAGEPFWAARGKRPAGDEDEDSQDRGEPLETISVDVPTWTAFGKKSALDDARQS
RDKCCQKESMSVERPLWASQKRGNDDDDDELEDKRGRRGSLLDSLSAEVPFWAARGKKDSPRMTPPSPEDLLSQIRTQES
PSKSDPWWPVRGKRIADTDSNPNPEDERFRHATENRRQNNTFNRNLAS

Zootermopsis Neuroparsin
MKSCAVCILASAFIFLLLQSCKAGSLICKPCMGNDCNLEPESCEHGIERDYCGWKVCAKGPGDYCGGPSDVRGKCGEGMH
CACGKCNGCSLTTLDCYFGLDQLQCL

Zootermopsis Neuropeptide F 1a
MQSSLAWFLVVGCALVLMPHVAPTAWAKPTDPEQLAAMADTLKYLQELDRYYSQVARPRFGKRADLRPVNEQDLAPDDSS
DRLWRRIASRR

Zootermopsis Neuropeptide F 1b
MQSSLAWFLVVGCALVLMPHVAPTAWAKPTDPEQLAAMADTLKYLQELDRYYSQVARPSPRSESGRQHELSRVENALKML
QLQELDRFYSPRTRPRFGKRADLRPVNDQDLAPDDSSDRLWRRIASRR

Zootermopsis Neuropeptide F 2
MQNPVNLLLVAGLCVVISMAMPCWSDPLPASAEIASRPTRPKVFTSPDQLRDYLQELGNYYAIEGRPRFGKRLDTPTYRP
SNNILGLPVTAGLSSAASRGNYFRYPTQSAVRNDLFQMLFPYDE

Zootermopsis Neuropeptide-like precursor 1
MWLSAPSRALLVGIAVLFIVFSPQARSEETPTGQHVVDKRHVGALARTGQLPFQGKRSYAALARNGDLPFNVREQWIKKT
HPMMTGSGGRYLEDLLLPPTTKRYVGSLAKSGGLPFSRVEGKRTDEGSEASEVDNLLQSVLETEDLWRLQLTALKQELLR
EQEEELENQLQEDDTDEEKRNVGSLARSGNMPFKNGKRSVEALARAGYLPVPKQPQESADYPHDSNEDSEELVGKRSIAV
LAKNGQLSAHGLKDLFHEGGNRGDDAYLEYLHQKREGDEAGEGLDELIQELYQEGQETGKRNIGSLARGYNFPYHGRKRT
LGSIVRSGGFRFGGATKKVGDDDKRSVTSLIRHRINPFQEGKRYIGSVMRNQGSHFGLSKKDDSELEDDAKRNIGAMVRN
WYLPEHLKYGKRPNDEDDEVEDDTAKRSVPTGLKDEKQVQATATAKQKRTKRQAFLVPATSSDEYPMPVMQNSDLFDYED
LAELLSGGAAPEKRFLGRIPQMGTKRPRTTPFPGRKSPHARNI

Zootermopsis Orcokinin A transcript
MAAAPACVHSSMKILPLVLVAVAAVLSVPGKATSAQGDPLRGSAFRDYRGDNVENEDNVPASQLHSILAGREVDGLAPFP
RKTRSGLDSLSGVTFGWNKRLDSLRGITFGNQKRNFDEIDRSGFNSFVKKNFDEIDRSGFDGFVKKNFDEIDRVGFGSFV
KRNAPFLLARSYEKENH

Zootermopsis Orcokinin B transcript
MAAAPACVHSSMKILPLVLVAVAAVLSVPGKATSAQGDPLRGSAFRDYRGDNVENEDNVPASQLHSILLIQTEAKTLNEV
LIQSGVNIDSIGGGNVLRNIDSIGGGNVRITDSLGGGNIVRNIDSIGGGNVLRNIDSIGGGNVRITDSLGGGNIVRNIDS
IGGGNVLRNIDSIGGGNVRITDSLGGGNIVRNIDSIGGGNVVRYIDSIGGGNVVRYIDSIGGGNVVRYIDSIGGGNVVRY
IDSIGGGNVVRYIDSIGGGNVVRYIDSIGGGNVVRYIDSIGGGNLIRGLDSIGGGNVIRGLDSIGGGNVVRNIDSIGGGN
LVKGLDSIGGGNVVRSSDAGSG

Zootermopsis Periviscerokinin
MKQILFSCAMIHVLLLVSSVQCDEESDSVSTNTDAERGSSGLIPMPRVGRSDLAWTLQRQGDTVPSSLINRRSSSGLISM
PRVGRGFLGLAPGVRTDPYLKDKRGSSGLIPMPRVGRSDVFWPLTDAFNVDGNKVGGLEKNINGDAGKTSTGMWFGPRLG
KRREGNIDIPWAIVTVKEIPADVRDYTPYLTRESEGKEDYRVLLDEELPVRSGRIIHGHE

Zootermopsis Pigment dispersing factor
MKQLGAVILFFYLLTTEFTSAAIQLEDNRYLDKEFQTNAVNVRELATWIMQLLLHKGQQTICTHKRNSELINTLLGLPKI
LNEAGRK

Zootermopsis Proctolin
MCCRQAVLLALMLVVMYAATEARYLPTRSQDDRLDRLRELLRDLLESEVEKTNVNNNSYDRRMLYKRQVPMITTEQQQAP
LVSAQQ

Zootermopsis Prothoracicotropic hormone
MGDLLVEENMSLSSMTSRPRFQMMTCLALLFFSAVCNSNAINPFPKRWEGQDSPITGRHVPEIGDCTEGPCLEWGNFQDD
ERLLLLLNNIDEDNINGEEALVKRGPPSPLNAEFSLIPVPDTRARFRNSNPSQCSCRSDTTTQLDLGFGYFPRYWSHLIC
NDESCGSPHYRCVAMNYTVFALRTSITVDEAVRHTVNPNNRFEGVNVTVACVCQRHYPQG

Zootermopsis Pyrokinin
MRTDFSTQQHLIHTIVLLCLVVALASCDGFRLSSDPLEDGLLLGLEGLGDDPLAAKRGEPEVTGMWFGPRLGRREKRSVD
DFPEDVADIRVEEVMELLKDTPWALLPLRGGKRHIEGFVPRLGRDSNEDEDADMMEQRSPPFAPRLGRRLVPFRPRMGRD
RLPHDVYSPRLGRSVPHEKKQTPPHH

Zootermopsis Relaxin-like peptide A transcript
MLLPLTTVTALCVLLDFSESTNTEKELEEMFKARSDDDWLNVWHQEHHTRCQETLLRHLYWACEKDIYRLSRRNDNQDQG
IEFLQGKSDPRYPFLSVVEARVFLRDRRRQQRRRGSGASITDECCLNTAGCTWEEYAEYCPANKRLRKFV

Zootermopsis Relaxin-like peptide B transcript
MLLPLTTVTALCVLLDFSESTNTEKELEEMFKARSDDDWLNVWHQEHHTRCQETLLRHLYWACEKDIYRLSRRNDNQDQG
IEFLQAPNLNLVYLAGYPFLSVVEARVFLRDRRRQQRRRGSGASITDECCLNTAGCTWEEYAEYCPANKRLRKFV

Zootermopsis RYamide
MASASSVVILIMLVTCSLVTLALSAQFYTSGRYGKRDLAQRSMFWSGSRYGRSSGGGGGRRQGGNNPVEVAVRNDRFFIG
SRYGKRSEEPLTTTDETVGVLVPTEDTNSQVACMYTGVANLYRCYKRKGNSSEDASSEHE

Zootermopsis short Neuropeptide F
MHCFPTIRCCTIALCLVIVAAEFVTSAPSYSDYESVRDLYELLLQKEALENRMQQQGQHEIVRKANRSPSLRLRFGRRAD
PLLTGSPFSEHSSVESAITEN

Zootermopsis SIFamide
MQNRVVATCVLLLAVLLLAEFATAAFRKPPFNGSIFGKRGSPTDYDGASKALSAMCEIASEACSAWFPQMD

Zootermopsis SMYamide
MKLSCSMIFLLALLLALLVDCNTGPPHRRVPFNGSMYGKRTANSLSVDYDSNAKSLSSLCEVATEVCSAWFPQQTENN

Zootermopsis Sulfakinin
MVATLILTLGVYLVLQYQHHAAVDAAPSSSDVVAAGGSNLEGPGQRGRSRSFLQTPPRSPQYMRARLVPVEPAADILNDF
IIDDESMDFNKRQSDDYGHMRFGKREQFDDYGHMRFGRSLD

Zootermopsis Tachykinin
MLTPRVRCRACAVLVVTLSLVAVVLCAPEESPKRAPSGFLGVRGKKDSAFVSEEAYNDVMEKRAPAMGFQGVRGKKDDKR
GPSMGFHGMRGKKDADSRAEFLQELLQDKRAPSMGFMGMRGKKEALDFDYFDKRAPSLGFQGMRGRRDGEYLSANRLGLI
GVRVENGVNLEGDDYAEMSSDEDLEAGLQDAEEFSKRAPAANGFFGTRGKKVPANGFFGTRGKKGPSAGFFAMRGKKAPS
AGFMEYQGPPVDLDTLLNYLGTAYQHGRDKRNGGRLPGSKKAPIGFLGTRGKKDWPTQQVVASSEGPEFDPHTSQLSESD

Zootermopsis Trissin
MVGSPQITLLMTGMMLWWVCTWSVALSCDSCGRECQASCGTRNFRTCCFNYLRKRSGGGAAAEEEGDGPRLRLELRVVPD
PAARDWDLAEEPDTKPPAEPDTVPPPSGRMRLFYNA

Zootermopsis Tryptopyrokinin
MNTQLLCSQHILIWFLVSAAVNNLCSVNTAMEETRIPGSDYGTVDGQRPSCKNKKSPAADVINLSVEFNMDHKNEKPNGT
AESQPHIKLEDKIMNNFILENWSIPAEIIKNKTTTSLFTGQGDVETLMLQPTSNRMWYAPRLGRRDKRSRVNDEQVKQES
ARGGMWFGPRLGRRDKKSSINDEQVKQEFAYGGMWFGPRLGRRDKKSNVNDEQVKQEVAYGGMWFGPRLGRRDKKSNVHD
EQVKQEVAHGGMWFGPRLGRREKKSYENEWPKKPRPFWMFLETTNKPREYDFLPQFDQDTVSEIYATAVNNITSELENYS
SQLSDDIGIMKYSPSSRVRFSSQLGHKQKKATNVIAYVPQEVQMWTSEDKNNKPPEYDFTPRLGRELYEKGLTLASKSLR
LPQIGHNTGIKYVYFQQD

Zootermopsis Vasopressin-like peptide
MKMQLGTATLLAVFISLCTACLITNCPKGGKRAGTHSQELHTIRQCARCGPAKLGHCYGPAICCGPQIGCLVATPDTARC
LSEAASPVPCTAPTGAQCGEGKFAGRCTANGVCCTHESCHIDITCQLTTSDAPELIDVSADQTNPLYSLYSSYQQENPGL
GLSE

Supplementary Figure 2. Predicted *Locusta* neuropeptide and neurohormone precursors

Locusta Adipokinetic hormone 1
MVQRCALVVLLVVVVAAALCSAQLNFTPNWGTGKRDAADFADPYSFLYRLIQAEARKMSGCSN

Locusta Adipokinetic hormone 2
MTQGCTLTLTLVLVVAMLAALATAQLNFSAGWGRRYADPNADPMAFLYRLIQIEARKLAGCSD

Locusta Adipokinetic hormone 3
MQVRAVLVLAVVALVAVATSRAQLNFTPWWGKRALGAPAAGDCVSASPQALLSILNAAQAEVQKLIDCSRFTSEANS

Locusta Adipokinetic hormone 4
MRGLAVLAVLLAVGAALCSAQLTFTPSWGKRALPDAAFPSAEPFLYLYKLIQAEAQKMAGCSKFPN

Locusta AKH/Corazonin-related peptide
MIARLLLTLTVTAWCCYLVASQVTFSRDWSPGKRSPEPACAKHAATICQLLLNELRQLAACEVKSLLRYHAEEVNVPVPQ
EIYIDGNGGR

Locusta Allatotropin
[???]PRTIRGFKNVALSTARGFGKRDGNQLEAALAGRDAALPDSFPVEWFAAEMQNNPELARMIVSKFVDANQDGELTA
EELLRPTY

Locusta Allatostatin A
MNSRPSSSEAARLPLPALVLLLLFATAVMPQEVPGDALTGGPPSAQVSATGEAAAGSPP[???]VAAAAPLDDDGEYDLY
KRLYDFGVGKRAYTYVSEYKRLPVYNFGLGKRATGANALYSFGLGKRGPRTYSFGLGKRGDDEPGDYSEQE[???]DLVD
KRGRLYSFGLGKRVRPYSFGLGKRAGPATSRLYSFGLGKREGRRRFSFGLGKRAPAEHRFSFGLGKRDP

Locusta Allatostatin B
[???]SAWGKRAWSNLNGAWGKRGGDDEATWPELPDQTITEDEDGDHDLMQMQMPLPLAMQLQGDEGEPGDEQKRAWSSL
HGAWGKRAADWRAFHGSWGKREPGWTNLKGLWGKRAGPSNWNRLPAVWGKRSEDE

Locusta Allatostatin C
MAMSTAVKAVLLLVVALAATCWARAEPLGQQPSDKARLLNELDLVDDDGSIETALINYLFAKQVVNRLRAQMDVSDLQRK
RSYWKQCAFNAVSCFGK

Locusta Allatostatin CC
MLLLLPALLAAAAAPAVAPSAEDALSVDSRAIRPPIPQPPDYQDYQAAVRYDEYPVVVPKRTALLLDRLMVDLKHLMDK
DRGEPQNPIDSGSSIGRMALQRRGQKTGQYWRCYFNAVTCFRRK

Locusta Bursicon alpha
[???]ARGPADECQLTPVIHVLQYPGCVPKPIPSFACTGRCSSYLQVSGSKIWQMERSCMCCQESGEREASVSLFCPKAK
AGERKFRKVSTKAPLECMCRPCTGVEESAVIPQEIAGYPDNGPLTAHFRKSQ

Locusta Bursicon beta
[???]EFDELGRLQRTCTSEVGVNKCEGACNSQVQPSVTTPTGFLKECYCCRESFLRERTVTLSHCYDPDGARLTAEGLA
TMDIRLREPADCKCFKCGDFSR

Locusta Calcitonin
MAGSRDILLRSAAILLAFAVALSCAGEDDENTLLERFDIFDVLVKAAKDRTIAKRDCDINAMDDSCVNGQWVGSGTDGDY
FGSDGSPGKRSHIVLPARHHIRLQVRADCSVNAMDDSCVNGQWVGSGTDGDYFGSDGSPGKRSFRRPAKHH

Locusta Crustacean cardioactive peptide
MSRALLLGGPALVLLACLQLAAADDVIMEKRDMDSPFLDRLFESKMKRPFCNAFTGCGKKRSDESVSTLLEMNSEPAVAD
LSRQILSEAKLWEAIQEARAELMARRRQHEMQTNRLGDFSRPLAVAQYRKKRAAAPPTPAQGIKPWRR

Locusta CCHamide 1
[???]SALSAPGRGAAADGACLSYGHSCWGAHGKRSGV[???]

Locusta CCHamide 2
[???]GCMAFGHSCFGGHGKRADLEPGAEGLEAAEAAAAALLGGQAETGAEAEPGAEGPRFRLSP[???]FLRQWVKGRC
WPVKIYIYIYTGYYANHI[???]

Locusta CNMa
MRARSTLALVLVCAALWLCPAVTGRAMLLPLPRDAARLAPAAPADADAEAEYDAVAGAAAANWPDDEPPPPGLSQEEQAR
LLRMLLRMMHERDRADHDAAVDAALAAAGPLLEPGEGADDADADAEDALPYGGPHAPDDKRNLQGMMPTLCHFKICHMGR
KRNQQKAVARAAH

Locusta Corazonin
MLRPWVSMALLAVACWWLSALWVSGQTFQYSHGWTNGRKRAGSGALVPAARLPLPATADLEGQQTQQPCRVRCLRLLLQA
AALPQVTH

Locusta CRF-OMP
MSPVRVLVAALLAVSWGG[???]ASWPHQQRRQALDEFAAAAAAAADAQFQ[???]QDEEEDGGRRVKRMG[???]TPRA
VRPQRLLLEIARRRLRDAEEQIKANKDFLQQIGKRSPHAAAADADEAAADAPPYGLRVAADSASDIAKDWASSDSRWNNQ
FTIHQ

Locusta DH31
MQLTTVVAALLAVVVLLATPARPAWANQLSNNYVSDYEMEQTAPLLSILELISKLRQTSSIAEDPAAKRGLDLGINRGFS
GAQAAKHLMGLAAAQYAAGPGRRRRAA[???]

Locusta Elevenin
MTKTHVLSLLMIVLCALCLAIGSAKGQKRVDCRMYPFAPICRGIMTKKRDLDSTGVASSAIQKSADYPDARQWSAPEQVA
MFPWLV[???]

Locusta Eclosion hormone 1
MALCRRTLLLVALLVVAVAVCVVPHVAASAVGVCIRNCAQCKKMFGPYFEGQLCGDACLKFKGKMVPDCEDAASIAPFLS
KLE

Locusta Eclosion hormone 2
MSGRLVTALLVVLVLAATLAPPAAGNAVSVCIRNCAQCKKMYGPYFEGQLCADACLKFGGKMMPDCEDAASIAPFLNKLE

Locusta Ecdysis-triggering hormone
MLLCKETLASLAVVLVVAAAAAAPEEGGGLLLKPHVARRSDFFLKTAKSVPRIGRRSDLFLKSAKSVPRIGRRTNLAPIE
AQDGGEWLWPGGADALPMPARRQAYYVRKDGQPVMWSDVARDVEENPDLWPWSDFDSGSVREVDGSR

Locusta FMRFamide
[???]RTNFLRLGRAGNAPSSFLRLGRARGGASGFLRLGRGSERNFLRFGRARQEGEEAPVSEEAEQLAREGRAGMADPL
TRHDRNFIRFGRSGPAPR[???]

Locusta Glycoprotein hormone alpha 2 (GPA2)
[???]MDGGRDAWEKPGCHRVGHTRKISIPDCIEFPITTNACRGFCESWSVPSALNTLRVNPHQAITSIGQCCNIMETED
VEVRVMCLDGPRDLVFKSAKSCQCYHCKKD

Locusta Glycoprotein hormone beta 5 (GPB5)
MAACRLVYVCCALVAAAAAAAAVADAAMDPASTLECHRRLYTYKVTKTDADGRACWDVINVMSCWGRCDSNEISDWRFPY
KRSFHPVCLHDARERRSVRLRNCEEGAAPGTERYDFLEAVSCRCAVCRSSEASCEGLRYRGQRSGPFKALGRR

Locusta Insulin-like peptide
MWKLCLRLLAVLAVCLSTATQAQSDLFLLSPKRSGAPQPVARYCGEKLSNALKLVCRGNYNTMFKKASQDVSDSESEDNY
WSGQSADEAAEAAAAALPPYPILARPSAGGLLTGAVFRRRTRGVFDECCRKSCSISELQTYCGRR

Locusta Ion transport peptide A-transcript
MHHQKQHQKQQGEASCQHFQWRLSGVVFCVLLVASLLSSAASSPLDAHHLAKRSFFDIQCKGVYDKSIFARLDRICEDCY
NLFREPQLHSLCSRKDCFTSDYFKGCIDVLLLQDDMEKIQSWIKQIHGAEPGV

Locusta Ion transport peptide B-transcript
MHHQKQHQKQQGEASCQHFQWRLSGVVFCVLLVASLLSSAASSPLDAHHLAKRSFFDIQCKGVYDKSIFARLDRICEDCY
NLFREPQLHSLCRSDCFKSPYFKGCLQALLLIDEEEKFNQMVEILGKK

Locusta Leucokinin
[???]GEVLRRRGCEHWRCSRWRWRRWRRRAWRGPRTAAKRDDDLADAKRAFSSWGGKRFADEAPAAEAKADEKRSFSSW
GESLANRRLDLYNYRPVQLRNGNGNGPPFFPWGG

Locusta Myosuppressin A transcript
MRSCLLLAVALAVACCGAWADAVPVARPIVCATDDVSPQIRKVCQAYEAFSELATSAKDYLDHFAAVRDPELFVEDKRPD
VDHVFLRFGRRRR

Locusta Myosuppressin B transcript
MRSCLLLAVALAVACCGAWADAVPVARPIVCATDDVSPQIRKVCQAYEAFSELATSAKDYLDHFAGEPRHQSLRPLPAGI
KREDVGHVFLRFGRRR

Locusta Natalisin
[???]APFWGARGRRREISSSSASAEEPFWAARGRREMELFWPARGKRHQQVQYPRRGLSKAFSGNSKNSEYASAGGQYF
VPARGKRD[???]

Locusta Neuroparsin transcript 1
MKATAALVAATLLLAVTLFHRAEANPISRSCEGANCVVDLTRCEYGDVTDFFGRKVCAKGPGEKCSEFEFCGVGLSCFNR
TCTGCSLHTLECFFYTDLTTEQE

Locusta Neuroparsin transcript 2
MKATAALVAATLLLAVTLFHRAEANPISRSCEGANCVVDLTRCEYGDVTDFFGRKVCAKGPGEKCGELELCGLGLSCFNR
TCTGCSLISLTCYSLR

Locusta Neuroparsin transcript 3
MKATAALVAATLLLAVTLFHRAEANPISRSCEGANCVVDLTRCEYGDVTDFFGRKVCAKGPGERCDDVEKCGAGMRCAQK
LCNGCSLVTLQCFTLVSLPISEDE

Locusta Neuroparsin transcript 4
MKATAALVAATLLLAVTLFHRAEANPISRSCEGANCVVDLTRCEYGDVTDFFGRKVCAKGPSEECSDFVKCGPGLRCQCG
RCTGCSLVKLTCYTDISTPTTCP

Locusta Neuroparsin transcript 5
MKATAALVAATLLLAVTLFHRAEANPISRSCEGANCVVDLTRCEYGDVTDFFGRKVCAKGPGDKCGGPYELHGKCGVGMD
CRCGLCSGCSLHNLQCFFFEGGLPSSC

Locusta Neuropeptide F 1a
MSQSRPLALLVVAALVAAAVLVAAAEAQQADGNKLEGLADALKYLQELDRYYSQVARPRFGKRAELRPDVVDDVIPEEMS
ADKFWRRFARRR

Locusta Neuropeptide F 1b
MSQSRPLALLVVAALVAAAVLVAAAEAQQADGNKLEGLADALKYLQELDRYYSQVARPSPRSGGAAALPVRSPLDSLSIA
EHLRGVEKMVRMLQLQEYDRMYTPRNRPRFGKRAELRPDVVDDVIPEEMSADKFWRRFARRR

Locusta Neuropeptide F 2
MCAVRALVLAAVALAAVAALSAPDVGQARPERPPMFTSPEELRNYLTQLSDFYASLGRPR[???]

Locusta Neuropeptide-like precursor 1
[???]ADKRSVASLARAGALLPGKRNIAAMAKNGLLGPSGPVLLDGEGEKRSVGALARSGLLPQPGRRAQDAGDDDLSLD
SLMQQLYSEEEKRHIGTLARDYSLPSYGKRNLGSLARSGGLSNVRYVTSKKDDSQPPADKRSLASLMRSRGPSPVEKRYL
ASLVRSHGLPYPLTKKEDDGPGEIKRNVGALARNWMLPSGKRNADAHDAQGVGK[???]

Locusta Orcokinin A transcript
MHAATLLVAALAAAAAAFAGALPAPQMVSSGFQQYRDEPNDVEEGLVRHLDNIGGGHLLRNLDGLGGGHLLRQTKSGLDS
LSGATFGEQKRLDSLSGITFGNQKRNFDEIDRSGFNSFIKKNFDEIDRSGFDRFVKKNFDEIDRSGFSGFVKRNAQMLGR
HYDKDD

Locusta Orcokinin-B transcript
MHAATLLVAALAAAAAAFAGALPAPQMVSSGFQQYRDEPNDVEEGLVRHLDNIGADYSFNDLQTLKAVFEHSRKESTRHH
NSDSSVVNQIGNTHTDRNSDSIGKGNLDRNIDQIGGGNLPRNIDSIGGGNLVRNIDSIGGGNLVRSIDSIGGGNLVRNID
TIGGGNLVRNIDSIGGGNLVRNIDSIGGGNLVRNIDSIGGGNLVRNIDSIGGGNLVRNIDSIGGGNLVRSIDSIGGGNLV
RNIDQIGGEHLVRNIDSIGGGNLVRNIDSIGGGNLVRNIDSIGGGNLVRNIDSIGGGNLVRNIDSIGGGNLVRNIDSIGG
GNLVRNIDSIGGGNLVRNIDSIGGGNLVRNIDSIGGGNLVRNIDSIGGGNLVRNIDSIGGGNLVRNIDSIGGGNLVRNID
SIGGGNLVRNIDSIGGGNLVRNIDSIGGGNLVRNIDSIGGGHLVRNIDSIGGGNLVRNIDSIGGGNLVKRSE

Locusta Proctolin
MLSQRKTVLLLVVALLAAGVAQGRYLPTRRSSASLEDRLDRLRDLINDLVESERPAPRALPPRRLDWMLAGEPELDYPQ

Locusta PDF
MAVSGKLITVFVLSIYVLGLALTIHATQYDEEKYQENEVRYGRELATLLAQLAHKNEPAICAHKRNSEIINSLLGLPKLL
NDAGRK

Locusta Periviscerokinin
MAAPSTSRAAAAALVAAAALALLAAAAAQDGDKGISKLKKTSSLFPHPRIGRSDYMNLGAGEVGEKRAAGLFQFPRVGRA
LVHGPLPFPLGVFSPLQLAPHQPADSAADLDDGNVSEQQQQQ[???]

Locusta Pyrokinin
[???]FKLSAVTALWASVLAAAGSFPAGAASAWRRQSVPTFTPRLGRDSAGDELAEEE[???]
[???]RDPPVDGPLVWLPLQVSPRLARRRQQPFVSRLGRDSGDEWPQQPFVPRLGRRLHQNGMPFSPRLGRDAAEQQPAD
E[???]

Locusta RYamide
MISRRIVLAFMLLAVVQAISLFDNVYEEPVSGGRQLPEGVLTIAQPESFALGSRYGKRNGNSNEETMSITNRDEDSSETE
DRQSMENYVVFLNNCLCSCKKPSGGLN

Locusta sNPF
MASTSAVCKLALVLLLVAALASCAPSYPDYDNVRDLYELLLQREAEGARLAAAAEDHQVVRKSNRSPSLRLRFGRRSDPL
FGAPSAAGSGQDSLAVAARSPSLRLRFGRRSDPLLPNQLGAPESAVEN

Locusta SGSSP
MALKTLAAFLLVVCLAQLTCASPAPAPNRRSTDESTADSSSATVTTSANNATSDADTISSNDTSSDDIFGEIGEKVEGFF
GHLFGKRDIEIPSDVFTKLYQEWAKGRPSRSVTVRDTGKNFTVGDLFQEWLQGNVNKRSVTVREVGNLFQEWLQGNVNKR
SVTVREVGDLFQEWLQGNVNKRSVTVREVGDLFQEWLQGNVNKRSVTVREVGDLFQEWLQGNVNKRSVTVREVGDLFQEW
LQGNVNKRSVTVREVGDLFQEWLQGNVNKRSVTVREVGDLFQEWLQGNVNKRSVTVREVGDLFQEWLQGNVNKRSVTVRE
VGDLFQEWLQGNVNKRSVTVREVGDLFEEWLQGNMNKRSVTVRDTDNSSTMGDLIKEWLKGNLNERSVTVRDTGKNFTVG
ELFQEWLQGNVNKRSVTVRDTDNSSTMNERSVTV

Locusta SIFamide
MQTAACSRFLVVVVLALVMFTAAASAAAATFRRPPFNGSIFGKRNSIESAGSSTAVAAVCEIAAEACAAWLNNDK

Locusta SMYamide
MNRCSLVAMLVWAILLQTCLTEGIAFQKLPFNGAMYGKRTTSVDFDSSNRAISSLCETASEVCASWYGQPDTN

Locusta Sulfakinin
[???]LADDLLEASKRQLASDDYGHMRFGKRQPPAAPAAPASVPVAPRFDDYGHFRFGKRRPQ

Locusta Tachykinin
MCRVGALLLLAALMSADPAAAQRQEAGEPRAAAPFLGMRTAAAAADADGGAADGLLEKRAPSLGFHGVRGKKDDLQELED
KRAPSLGFHGVRGKKDDANGLDDDDFDKRAPMRGFQSVRGKKDEAEVEDAELGDGDYLQLAGLPYREDYDADGDADSDGE
ELQLDDPWLRDQKRALKGFFGTRGKKAPQAGFYGVRGKKGPSGFYGVRGKKAPLSGFYGVRGKKAPSAGFHGVRGKKEDG
APAPDLDSLLYYLNEAGEAARQKRGNTKKAPVGFYGTRGKKSWAP

Locusta Trissin
[???]TQCWFPGALVVALCSATCDFCGRECSKACGTNYFRTCCLNYLRKRSGPPGLHLELMLLPPDGSAAR[???]

Locusta Tryptopyrokinin 1
MPRGAQLFLLLALVTAARVLDARAESSRAGSDAQSSHQDGRGASHDRNEESNELNDENRSDGDAQDATFRRGREMQEFVS
HAGGPIGNGEEDQWLALADGSYIPAEAVRELVQRGPTLSENTGLWFGPRYGRRTSCGENVPLKWLSQVEKRAAKQPALWF
GPRVGRSLDEEPKGEEWRDDDKGLKDGSSQRQDRSAQPPGLWFGPRVGRRSDAQVDDMLWFGPRHERSVDTDKQDLYDDE
VAMRDQRGAKHPGLWFGPRFGR

Locusta Tryptopyrokinin 2
MESSMDVLPVALLTLAALLFSVETSEVLHEVNDLNGSNRDSGEQTPSAVLSRSKRNNSGAHVDKWFRPDTGAGEDYWNAL
FREDAEDHLSKPDASTVDRLENVVPTAETPRLSVAQHESRHSEGSHDDHNDSEPEVWIGQHSGRSIPEPGTWFGPRIGRS
HTEPGLWFGPRYGRSYPEPGMWFGPRVGRGHPEPGMWFGPRVGRSQAEPGVWFGARIGRNQPEPGTWFGARIGRSHPEPG
MWFGPRVGRSHPEPGTWFGPRIGRSHSEPELRM

Locusta Tryptopyrokinin 3
[???]RNGRRQPDTALWFGPRVGRRMQHIQPEASEFFRPDVRRSGPEHSLWSDTQLRQGDPEPPLHVRISNPKQNLWFRT
DTRRRQSENILQFGPQVGRRNAESALWFGPRVGRSYLESSLSDERYVQHRNLEPGLWSNNLGQIQTTPSMDVANRFPGRD
LWSGTDLDRTKHETALWFGPRIGRSNPETNLWFGPRVGRSHPETSQSFGQYARHDNVETGLWPDDFVQSRPTTLEDVTKR
NPERNWWSVNGVGLTKHETTLWFGPRIGRSNPETNLWFGPRVVRSHPETSQSFGPYSRRNNVESGLWPDNLGQSRPTTWE
DVTKRYPGHNWWSVNGLGRTKHETALWFGPRIGRSNPDTHLWFGPRVGRSQSLASEQLR

Locusta Tryptopyrokinin 4
MLRRTHSSLAVAVTVVAISAFAVSAAKPREMHSKNVVVQRCIQTGFSDGGNIRSVPETSLWFGPRIGRSNLETALLFGRC
VGCSHPGTSQCCEPLARRSGSEDSLGYSSPEPPHDNRKINPESGLWFVDTLARRSGSEDSLGYSSPEPPHDNRKINPESG
LWFVDRNGRRQPDTALWFGPRVGRRMQHIQPEASEFFRPDVRRSGPEHSLWSDTQLRQGDPEPPLHVRISNPKQNLWFRT
DTRRRQSENILQFGPQVGRRNAESALWFGPRVGRSYLESNLSDERYIQHRNLEPGLWSNNLGQIQTTPSMDVTNRFPGRD
LWSGTDLDRTKPRYGLDHG

Locusta Vasopressin-like peptide
MKPQPAVVLALAVSLAAACLITNCPRGGKRASLQH[???]

Supplementary Figure 3. GPCRAs

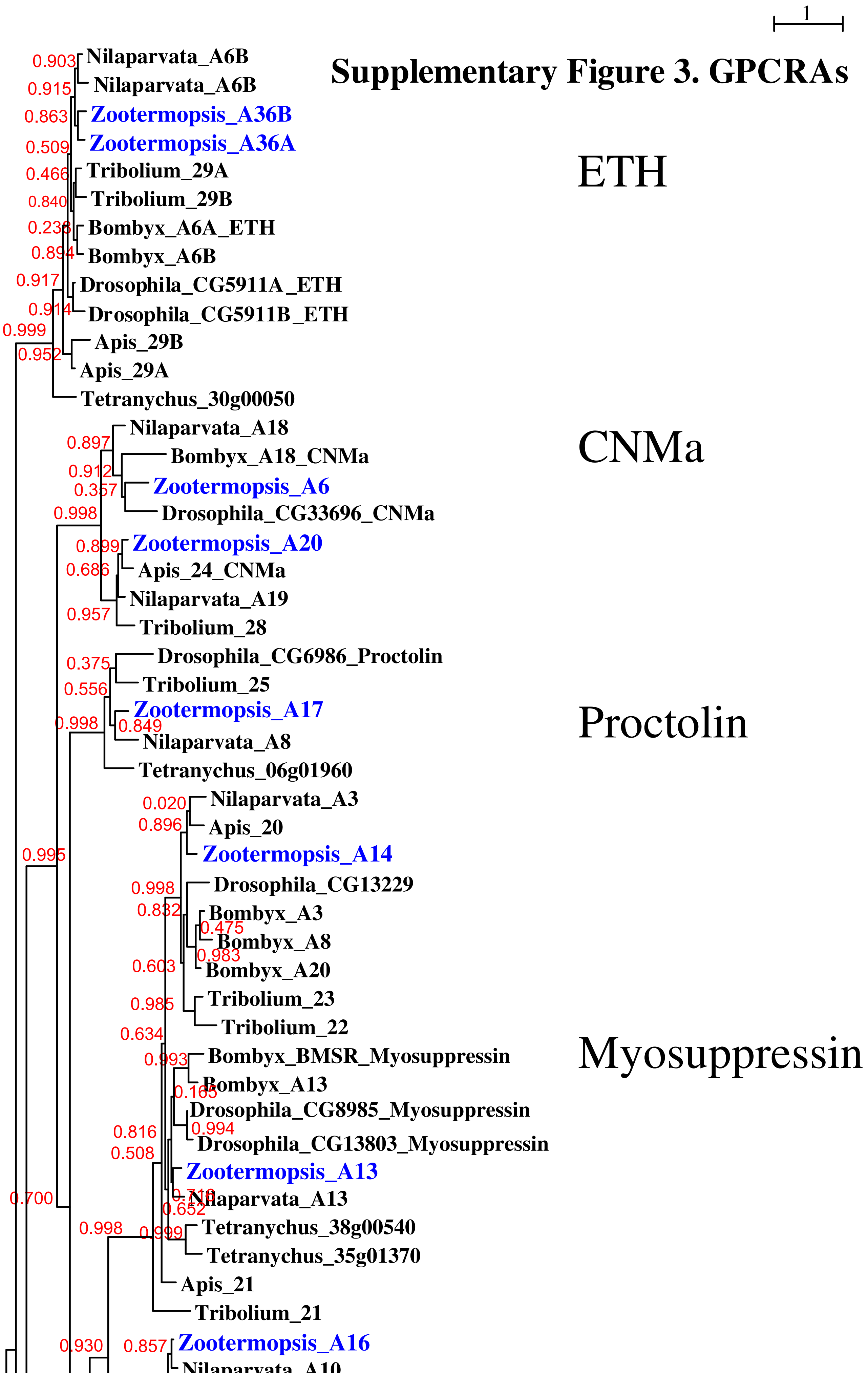

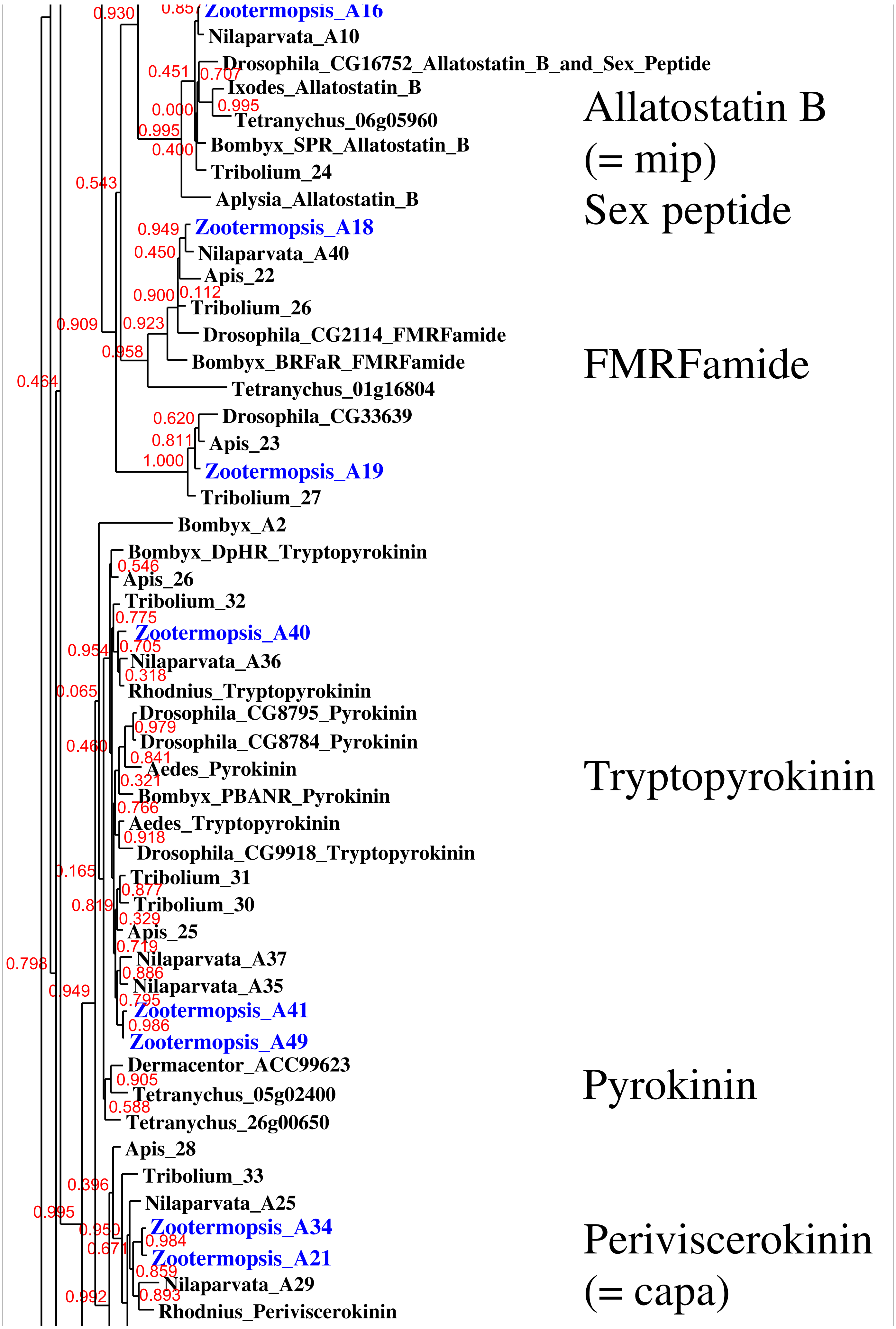
Zootermopsis_A16
Nilaparvata_A10
Drosophila_CG16752_Allatostatin_B_and_Sex_Peptide
Ixodes_Allatostatin_B
Tetranychus_06g05960
Bombyx_SPR_Allatostatin_B
Tribolium_24
Aplysia_Allatostatin_B
Allatostatin B
(= mip)
Sex peptide
Zootermopsis_A18
Nilaparvata_A40
Apis_22
Tribolium_26
Drosophila_CG2114_FMRFamide
Bombyx_BRFaR_FMRFamide
Tetranychus_01g16804
FMRFamide
Drosophila_CG33639
Apis_23
Zootermopsis_A19
Tribolium_27
Bombyx_A2
Bombyx_DpHR_Tryptopyrokinin
Apis_26
Tribolium_32
Zootermopsis_A40
Nilaparvata_A36
Rhodnius_Tryptopyrokinin
Drosophila_CG8795_Pyrokinin
Drosophila_CG8784_Pyrokinin
Aedes_Pyrokinin
Bombyx_PBANR_Pyrokinin
Aedes_Tryptopyrokinin
Drosophila_CG9918_Tryptopyrokinin
Tryptopyrokinin
Tribolium_31
Tribolium_30
Apis_25
Nilaparvata_A37
Nilaparvata_A35
Zootermopsis_A41
Zootermopsis_A49
Dermacentor_ACC99623
Tetranychus_05g02400
Tetranychus_26g00650
Pyrokinin
Apis_28
Tribolium_33
Nilaparvata_A25
Zootermopsis_A34
Zootermopsis_A21
Nilaparvata_A29
Rhodnius_Periviscerokinin
Periviscerokinin
(= capa)

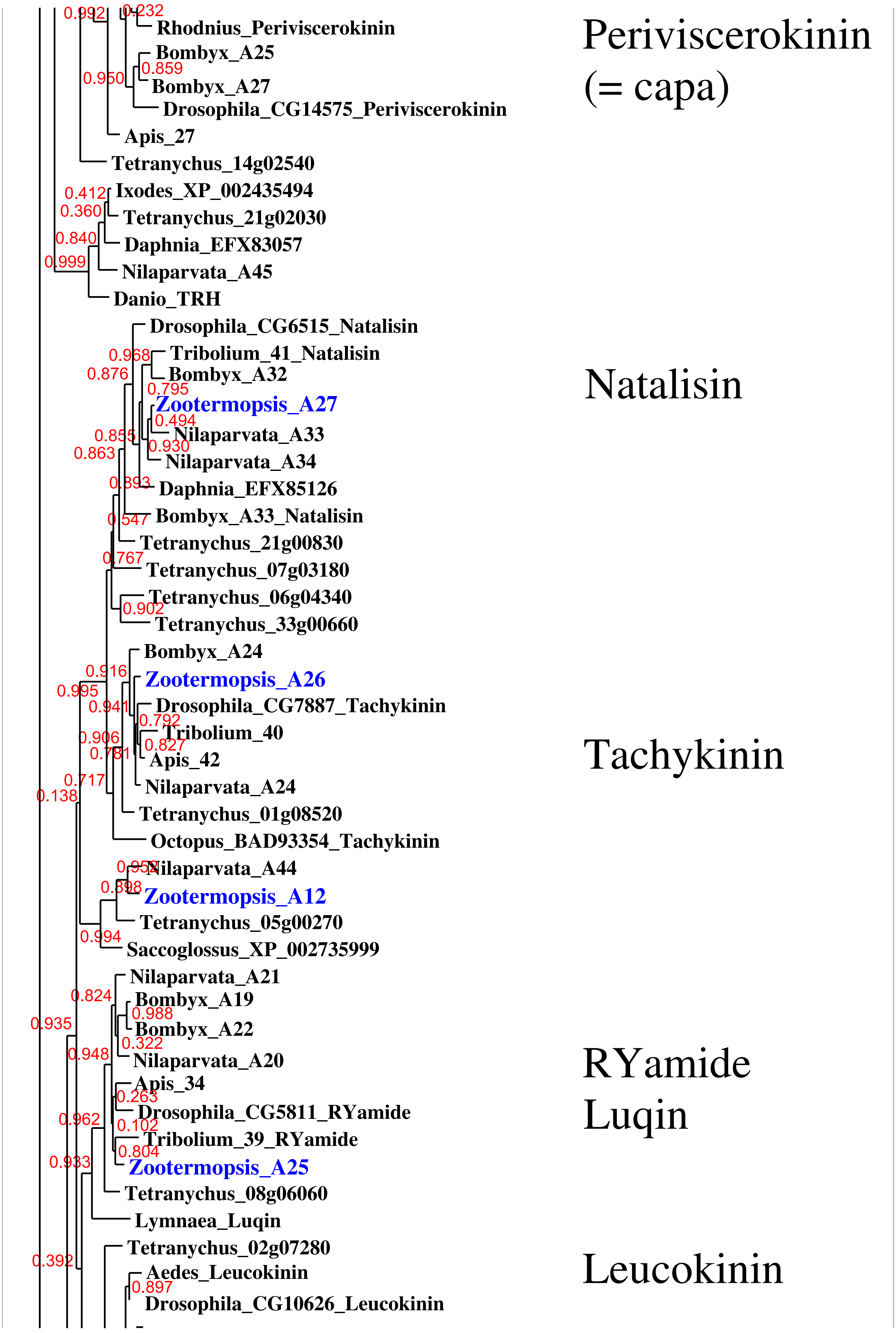
0.992
0.232
Rhodnius_Periviscerokinin
Bombyx_A25
0.859
0.950
Bombyx_A27
Drosophila_CG14575_Periviscerokinin
Apis_27
Tetranychus_14g02540
0.412
Ixodes_XP_002435494
0.360
Tetranychus_21g02030
0.840
Daphnia_EFX83057
0.999
Nilaparvata_A45
Danio_TRH
Drosophila_CG6515_Natalisin
0.968
Tribolium_41_Natalisin
0.876
Bombyx_A32
0.795
Zootermopsis_A27
0.494
0.855
Nilaparvata_A33
0.863
0.930
Nilaparvata_A34
0.893
Daphnia_EFX85126
Bombyx_A33_Natalisin
0.547
Tetranychus_21g00830
0.767
Tetranychus_07g03180
Tetranychus_06g04340
0.902
Tetranychus_33g00660
Bombyx_A24
0.916
Zootermopsis_A26
0.995
0.941
Drosophila_CG7887_Tachykinin
0.792
Tribolium_40
0.906
0.781
0.827
Apis_42
0.717
0.138
Nilaparvata_A24
Tetranychus_01g08520
Octopus_BAD93354_Tachykinin
0.952
Nilaparvata_A44
0.898
Zootermopsis_A12
Tetranychus_05g00270
0.994
Saccoglossus_XP_002735999
Nilaparvata_A21
0.824
Bombyx_A19
0.988
0.935
Bombyx_A22
0.322
0.948
Nilaparvata_A20
Apis_34
0.263
Drosophila_CG5811_RYamide
0.962
0.102
Tribolium_39_RYamide
0.804
0.933
Zootermopsis_A25
Tetranychus_08g06060
Lymnaea_Luqin
Tetranychus_02g07280
0.392
Aedes_Leucokinin
0.897
Drosophila_CG10626_Leucokinin
Periviscerokinin
(= capa)
Natalisin
Tachykinin
RYamide
Luqin
Leucokinin

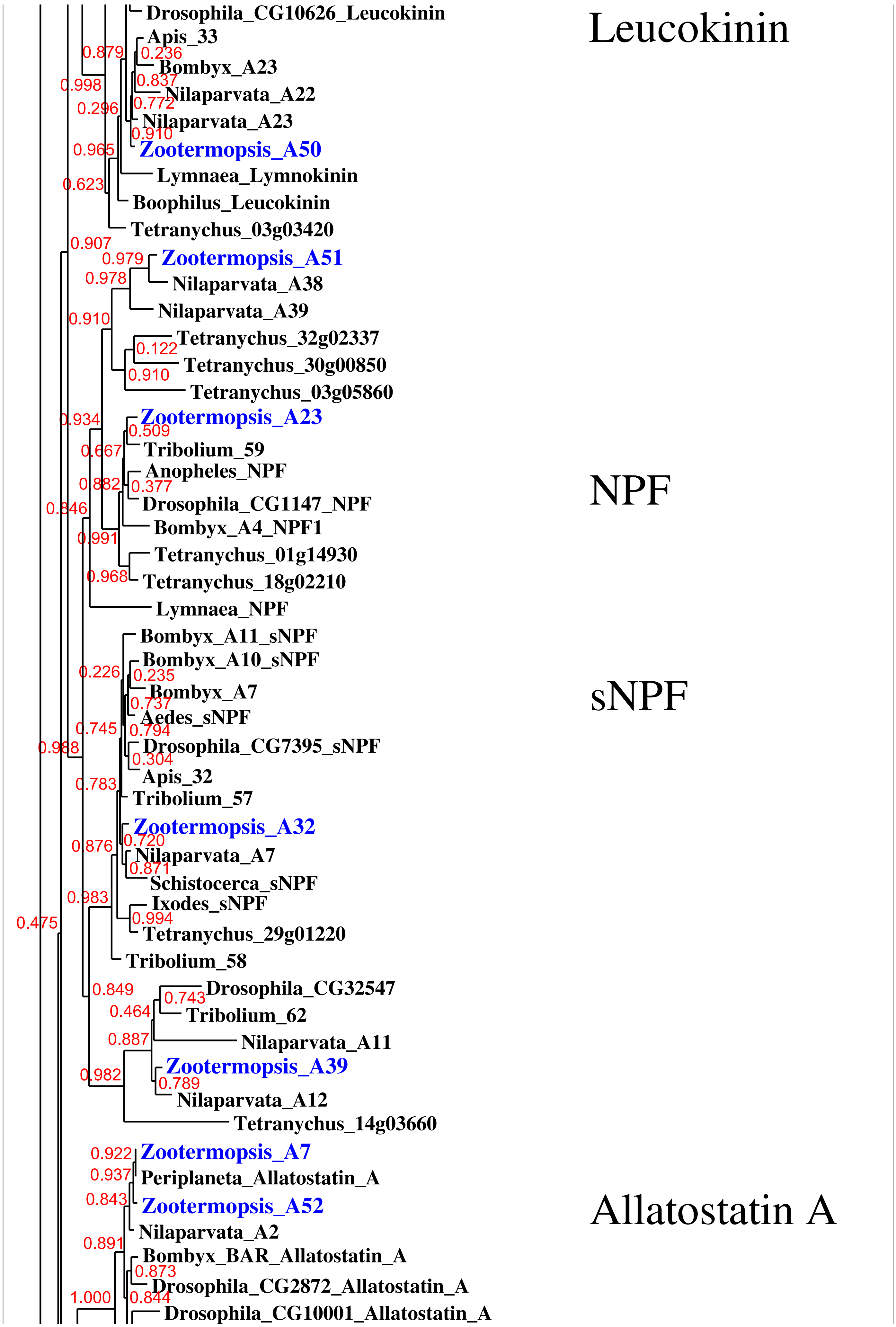
Leucokinin
Drosophila_CG10626_Leucokinin
Apis_33
Bombyx_A23
Nilaparvata_A22
Nilaparvata_A23
Zootermopsis_A50
Lymnaea_Lymnokinin
Boophilus_Leucokinin
Tetranychus_03g03420
Zootermopsis_A51
Nilaparvata_A38
Nilaparvata_A39
Tetranychus_32g02337
Tetranychus_30g00850
Tetranychus_03g05860
NPF
Zootermopsis_A23
Tribolium_59
Anopheles_NPF
Drosophila_CG1147_NPF
Bombyx_A4_NPF1
Tetranychus_01g14930
Tetranychus_18g02210
Lymnaea_NPF
sNPF
Bombyx_A11_sNPF
Bombyx_A10_sNPF
Bombyx_A7
Aedes_sNPF
Drosophila_CG7395_sNPF
Apis_32
Tribolium_57
Zootermopsis_A32
Nilaparvata_A7
Schistocerca_sNPF
Ixodes_sNPF
Tetranychus_29g01220
Tribolium_58
Drosophila_CG32547
Tribolium_62
Nilaparvata_A11
Zootermopsis_A39
Nilaparvata_A12
Tetranychus_14g03660
Allatostatin A
Zootermopsis_A7
Periplaneta_Allatostatin_A
Zootermopsis_A52
Nilaparvata_A2
Bombyx_BAR_Allatostatin_A
Drosophila_CG2872_Allatostatin_A
Drosophila_CG10001_Allatostatin_A

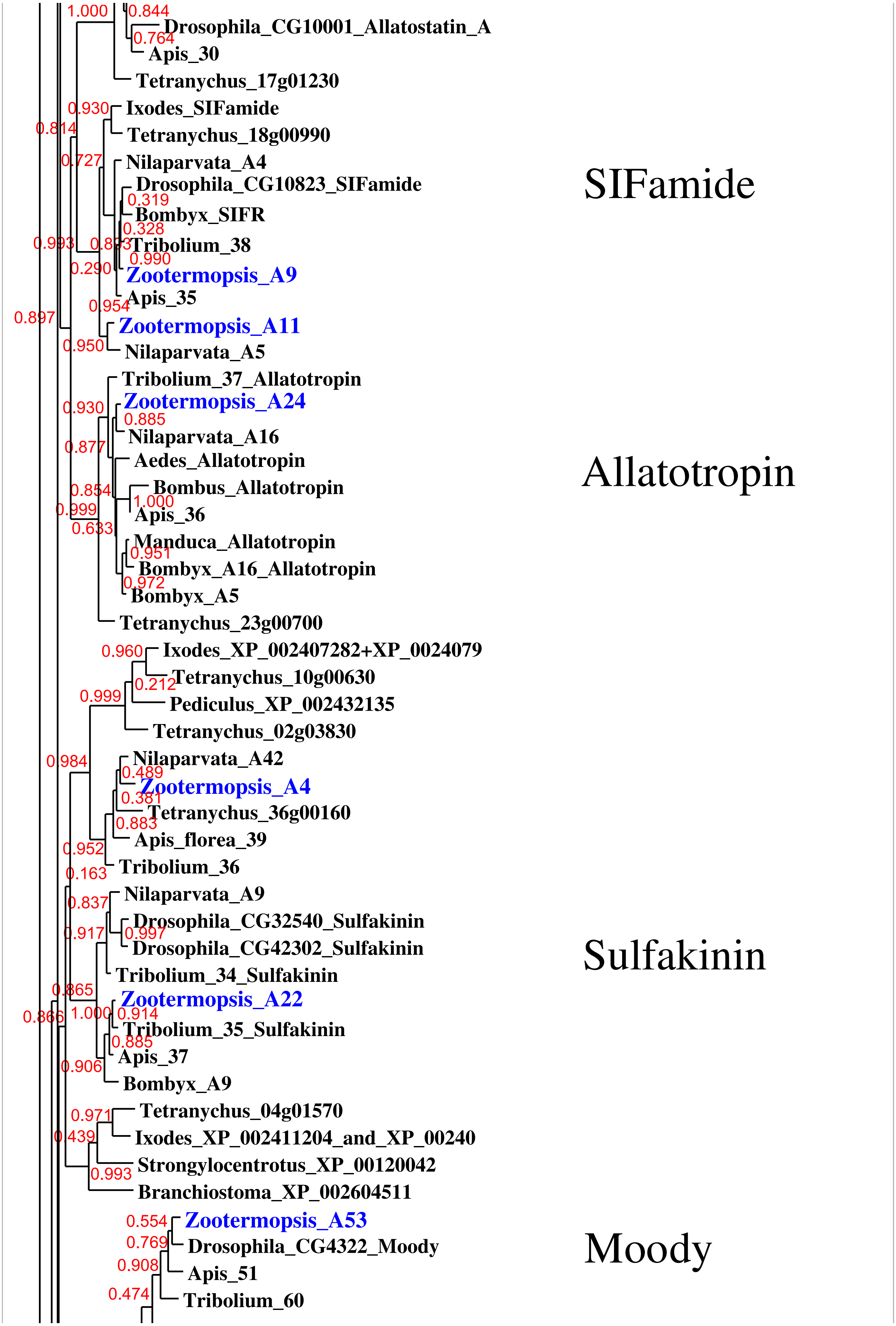
Drosophila_CG10001_Allatostatin_A
Apis_30
Tetranychus_17g01230
Ixodes_SIFamide
Tetranychus_18g00990
Nilaparvata_A4
Drosophila_CG10823_SIFamide
Bombyx_SIFR
Tribolium_38
Zootermopsis_A9
Apis_35
Zootermopsis_A11
Nilaparvata_A5
Tribolium_37_Allatotropin
Zootermopsis_A24
Nilaparvata_A16
Aedes_Allatotropin
Bombus_Allatotropin
Apis_36
Manduca_Allatotropin
Bombyx_A16_Allatotropin
Bombyx_A5
Tetranychus_23g00700
Ixodes_XP_002407282+XP_0024079
Tetranychus_10g00630
Pediculus_XP_002432135
Tetranychus_02g03830
Nilaparvata_A42
Zootermopsis_A4
Tetranychus_36g00160
Apis_florea_39
Tribolium_36
Nilaparvata_A9
Drosophila_CG32540_Sulfakinin
Drosophila_CG42302_Sulfakinin
Tribolium_34_Sulfakinin
Zootermopsis_A22
Tribolium_35_Sulfakinin
Apis_37
Bombyx_A9
Tetranychus_04g01570
Ixodes_XP_002411204_and_XP_00240
Strongylocentrotus_XP_00120042
Branchiostoma_XP_002604511
Zootermopsis_A53
Drosophila_CG4322_Moody
Apis_51
Tribolium_60
SIFamide
Allatotropin
Sulfakinin
Moody

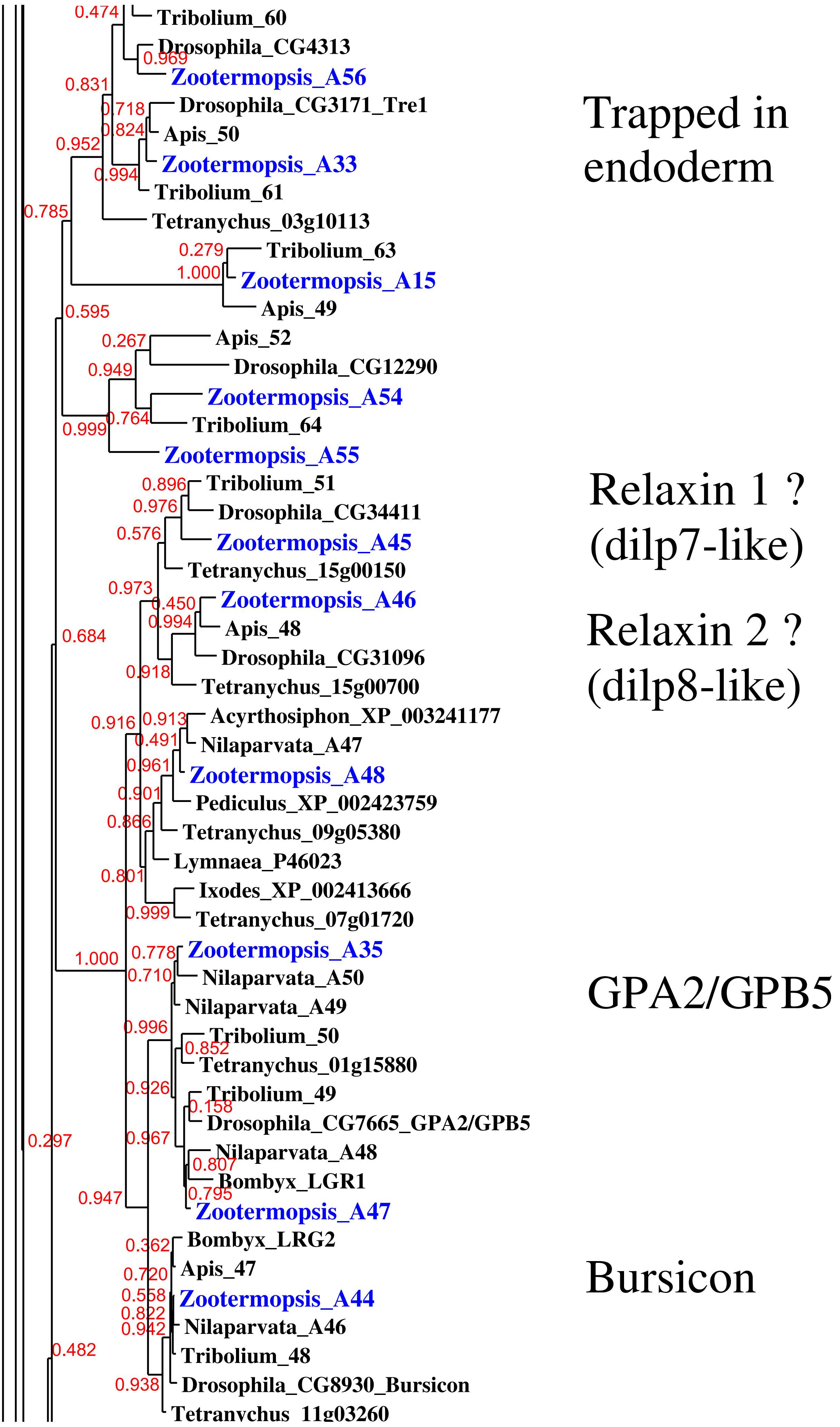
Tribolium_60
Drosophila_CG4313
Zootermopsis_A56
Drosophila_CG3171_Tre1
Apis_50
Zootermopsis_A33
Tribolium_61
Tetranychus_03g10113
Tribolium_63
Zootermopsis_A15
Apis_49
Apis_52
Drosophila_CG12290
Zootermopsis_A54
Tribolium_64
Zootermopsis_A55
Tribolium_51
Drosophila_CG34411
Zootermopsis_A45
Tetranychus_15g00150
Zootermopsis_A46
Apis_48
Drosophila_CG31096
Tetranychus_15g00700
Acyrthosiphon_XP_003241177
Nilaparvata_A47
Zootermopsis_A48
Pediculus_XP_002423759
Tetranychus_09g05380
Lymnaea_P46023
Ixodes_XP_002413666
Tetranychus_07g01720
Zootermopsis_A35
Nilaparvata_A50
Nilaparvata_A49
Tribolium_50
Tetranychus_01g15880
Tribolium_49
Drosophila_CG7665_GPA2/GPB5
Nilaparvata_A48
Bombyx_LGR1
Zootermopsis_A47
Bombyx_LRG2
Apis_47
Zootermopsis_A44
Nilaparvata_A46
Tribolium_48
Drosophila_CG8930_Bursicon
Tetranychus_11g03260
Trapped in endoderm
Relaxin 1 ? (dilp7-like)
Relaxin 2 ? (dilp8-like)
GPA2/GPB5
Bursicon

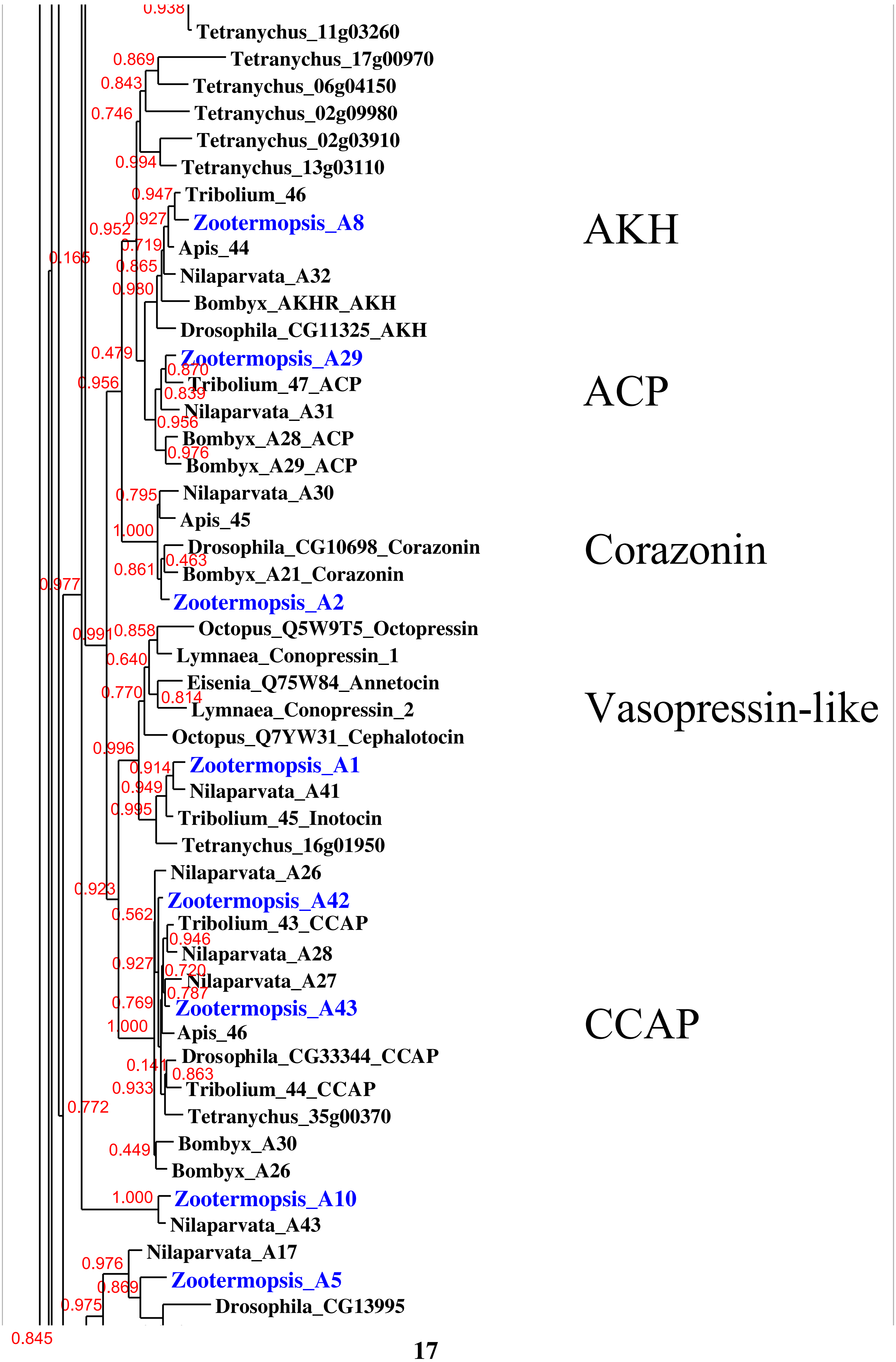

Tetranychus_11g03260
Tetranychus_17g00970
Tetranychus_06g04150
Tetranychus_02g09980
Tetranychus_02g03910
Tetranychus_13g03110
Tribolium_46
Zootermopsis_A8
Apis_44
Nilaparvata_A32
Bombyx_AKHR_AKH
Drosophila_CG11325_AKH
AKH
Zootermopsis_A29
Tribolium_47_ACP
Nilaparvata_A31
Bombyx_A28_ACP
Bombyx_A29_ACP
ACP
Nilaparvata_A30
Apis_45
Drosophila_CG10698_Corazonin
Bombyx_A21_Corazonin
Zootermopsis_A2
Corazonin
Octopus_Q5W9T5_Octopressin
Lymnaea_Conopressin_1
Eisenia_Q75W84_Annetocin
Lymnaea_Conopressin_2
Octopus_Q7YW31_Cephalotocin
Vasopressin-like
Zootermopsis_A1
Nilaparvata_A41
Tribolium_45_Inotocin
Tetranychus_16g01950
Nilaparvata_A26
Zootermopsis_A42
Tribolium_43_CCAP
Nilaparvata_A28
Nilaparvata_A27
Zootermopsis_A43
Apis_46
Drosophila_CG33344_CCAP
Tribolium_44_CCAP
Tetranychus_35g00370
Bombyx_A30
Bombyx_A26
CCAP
Zootermopsis_A10
Nilaparvata_A43
Nilaparvata_A17
Zootermopsis_A5
Drosophila_CG13995

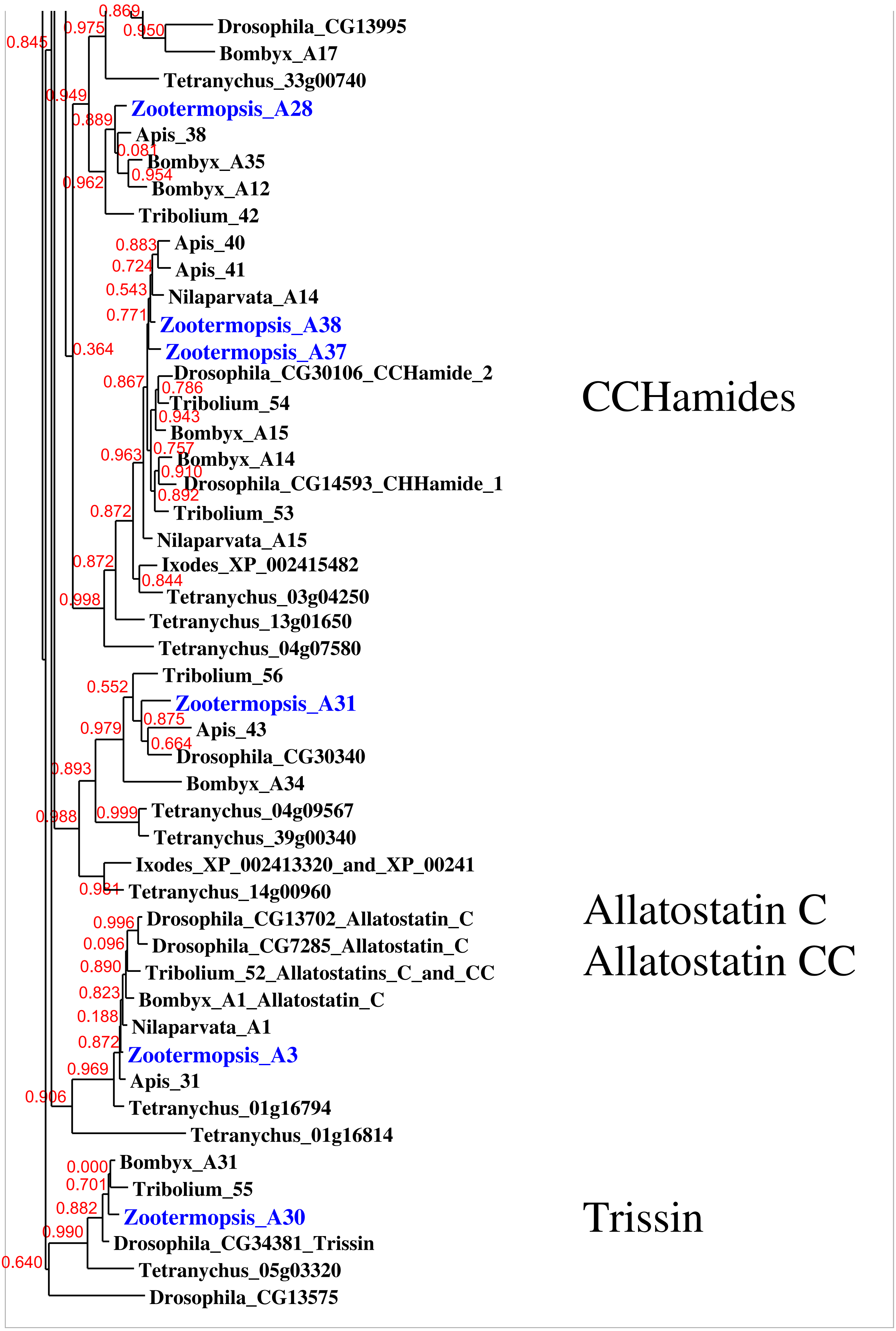

Drosophila_CG13995
Bombyx_A17
Tetranychus_33g00740
Zootermopsis_A28
Apis_38
Bombyx_A35
Bombyx_A12
Tribolium_42
Apis_40
Apis_41
Nilaparvata_A14
Zootermopsis_A38
Zootermopsis_A37
Drosophila_CG30106_CCHamide_2
Tribolium_54
Bombyx_A15
Bombyx_A14
Drosophila_CG14593_CHHamide_1
Tribolium_53
Nilaparvata_A15
Ixodes_XP_002415482
Tetranychus_03g04250
Tetranychus_13g01650
Tetranychus_04g07580
Tribolium_56
Zootermopsis_A31
Apis_43
Drosophila_CG30340
Bombyx_A34
Tetranychus_04g09567
Tetranychus_39g00340
Ixodes_XP_002413320_and_XP_00241
Tetranychus_14g00960
Drosophila_CG13702_Allatostatin_C
Drosophila_CG7285_Allatostatin_C
Tribolium_52_Allatostatins_C_and_CC
Bombyx_A1_Allatostatin_C
Nilaparvata_A1
Zootermopsis_A3
Apis_31
Tetranychus_01g16794
Tetranychus_01g16814
Bombyx_A31
Tribolium_55
Zootermopsis_A30
Drosophila_CG34381_Trissin
Tetranychus_05g03320
Drosophila_CG13575
CCHamides
Allatostatin C
Allatostatin CC
Trissin

Supplementary Figure 4. GPCRBs

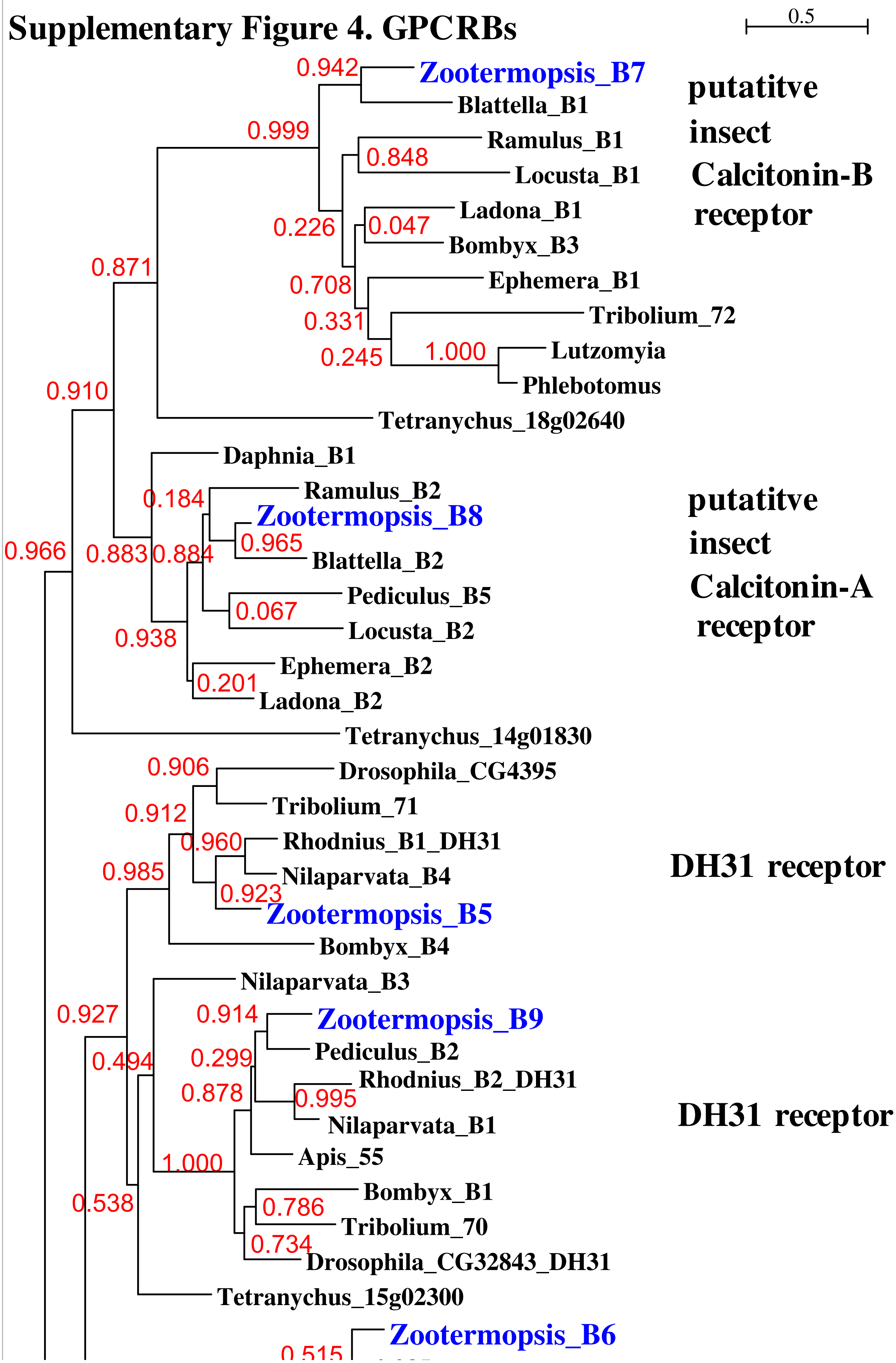

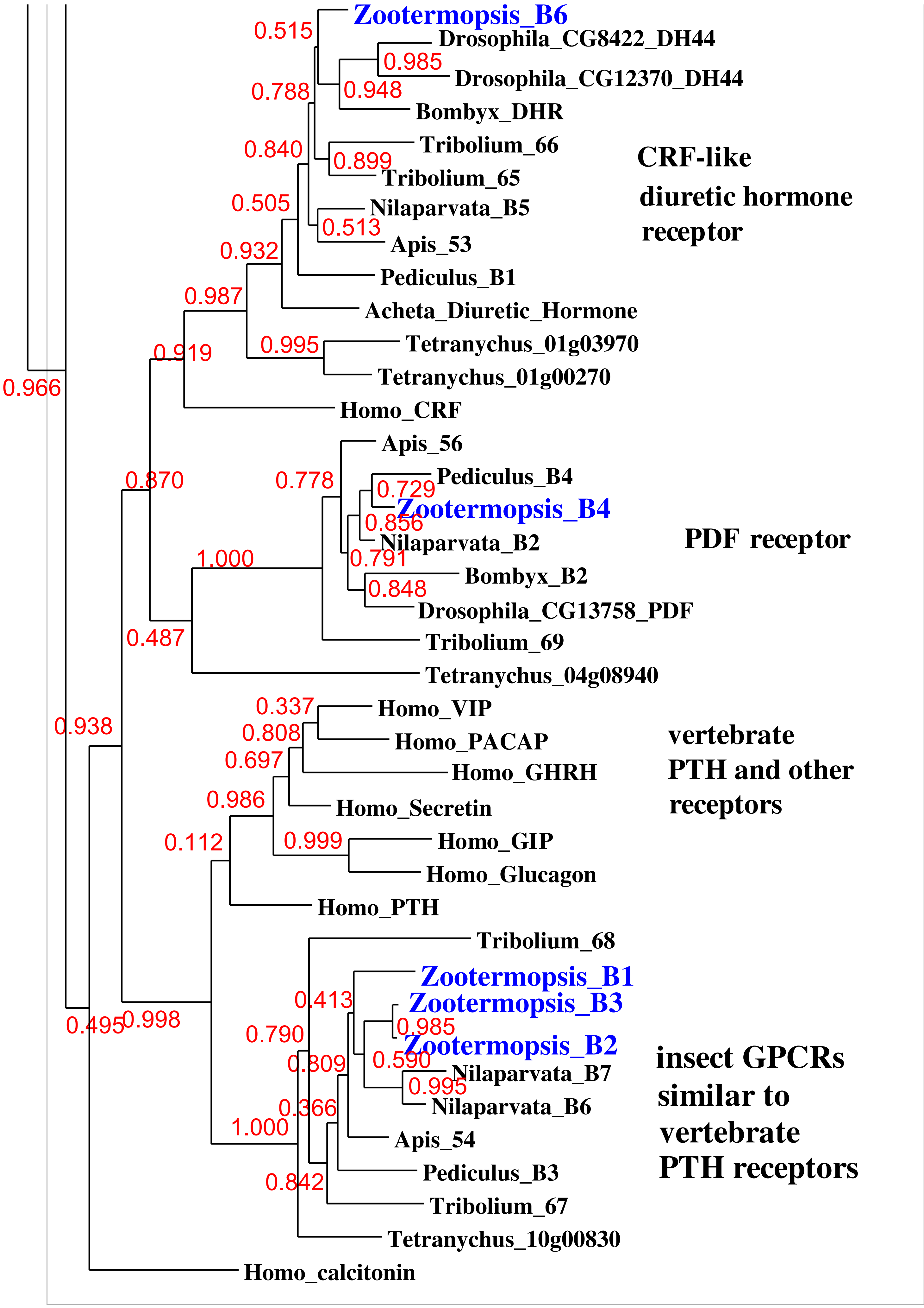
Zootermopsis_B6
Drosophila_CG8422_DH44
Drosophila_CG12370_DH44
Bombyx_DHR
Tribolium_66
Tribolium_65
Nilaparvata_B5
Apis_53
Pediculus_B1
Acheta_Diuretic_Hormone
Tetranychus_01g03970
Tetranychus_01g00270
Homo_CRF
Apis_56
Pediculus_B4
Zootermopsis_B4
Nilaparvata_B2
Bombyx_B2
Drosophila_CG13758_PDF
Tribolium_69
Tetranychus_04g08940
Homo_VIP
Homo_PACAP
Homo_GHRH
Homo_Secretin
Homo_GIP
Homo_Glucagon
Homo_PTH
Tribolium_68
Zootermopsis_B1
Zootermopsis_B3
Zootermopsis_B2
Nilaparvata_B7
Nilaparvata_B6
Apis_54
Pediculus_B3
Tribolium_67
Tetranychus_10g00830
Homo_calcitonin
0.515
0.985
0.948
0.788
0.899
0.840
0.513
0.505
0.932
0.987
0.995
0.919
0.966
0.870
0.778
0.729
0.856
0.791
1.000
0.848
0.487
0.337
0.808
0.697
0.938
0.986
0.112
0.999
0.413
0.985
0.790
0.590
0.809
0.995
0.366
1.000
0.842
0.495
0.998
CRF-like diuretic hormone receptor
PDF receptor
vertebrate PTH and other receptors
insect GPCRs similar to vertebrate PTH receptors

Diptera *Phlebotomus*
MKCERVFILGIFLFIFLAFECQCFPQPLSQNEISWSDFRDKLHRLENYYNFLKEMDQTSSRIQRSALCANIWDDGCINGQ
VIGAGSDSGFLEGGGTPGKRFIS

Lepidoptera *Bombyx mori*
MKYFIVIFCLVSLVCSKSFGHHPATYGDRRCVNMWDEGCINGQLPGSGWDDYYLNGNFNPGKRCLNMWDDGCANGQLPGA
GNDDYYLGGGFNPGKRCVNMWDEGCINDQINGAGNDDYYLNNGFNPGKRSVIELFKKLHKTKPKL

Coleoptera *Tribolium castaneum_1*
MKTAFLLLFVLMPAVLCYYVHPNLHYFPARVNYRSAPAKSWGALFHRLQLASKRCANTFDESCINDVINGAGSDEAFLNG
GDNPGKWGWVRRGVLNGGDNPGKRCVNTFDESCSNGDINGAGSDDDWLHGDDTPGRR

Tribolium castaneum_2
MRPVLVLVMIFSVSYARYLEPYNYGYPRPLVDFLNRLSLSDKIKRCGNTFDESCANLPIIGASSDESWLAHSSPGKRCAN
VWGESCINGGIIGGGSDQSWLQGDDNPGRR

Phthiraptera *Pediculus humanus*
KRTACLLDGGLSASCNHKDAVEAGEHHRFLASSFAPGRRKRVEQISPETPHYRK

Phasmoptera *Ramulus artemis* A
MTQQAAGSTSTMQLPLVLLMATFVVVVVATPAQLNNDLLAAHMRAIQQQRRTVQMLRDLLSELDAGMATVQKRTCYMNAG
LSHGCDYKDVLGAIDEEKYWRSVNSPGRRRKRAAEINKAMSK

Ramulus artemis B
MTQQAAGSTSTMQLPLVLLMATFVVVVVATPAQLNNDLLAAGGAARDKRCANMWDSSCTNSQTNGASRDDQYLTSGNNPG
KRCVSVWDDSCRNGEGNGASRDDEYLHGGNTPGKRCANIWDSSCTNSQANGASKDDEYFHAGNTPGKRYAIWDTSDLNGE
ANGSSRDDDNFHSSNTAGKRYANIWDTSVSNGETNGASRDDQYLVSGNNPGKRCANLWDVSCDNGQANGAGKDDEYFRPS
EIPGKRDADFEMWMVARFCAQHPSAAACRRLSSG

Archaeognatha *Lepismachilis y-signata*
MAGSRDILLRSAAILLAFAVALSCAGEDDENTLLERFDIFDVLVKAAKDRTIAKRDCDINAMDDSCVNGQWVGSGTDGDY
FGSDGSPGKRSHIVLPARHHIRLQVRADCSVNAMDDSCVNGQWVGSGTDGDYFGSDGSPGKRSFRRPAKHH

ARACHNIDA *Tetranychus urticae*
MKVCLIYISFGCFFVSSLPLNGHSSDPGQSLGDYTFTGLADDSQQDPSSPSLTPSSSSLSGSPSAGSVAGLISANGNNLD
SAKEWIQLRNLLSELGQQKVLTPSTYRTWKRSCTIDAGMTYNCDFRQLLSAVNARKYLESSLTPGRRKRPFSTIYNQPFN
Y

Ixodes scapularis
MAVQCKTVALLAFVLVACAFVAVNADMTEDIRQIVNRRKGLEVIRDIVDDLHRQLTTLHKRSCHIDAGLNRGCDYKDILD
AVEENKFWKSRDSPGRRRRSVDDTKTKSAPSSGRATTANMARVGQSTAN

NEMATODA *Caenorhabditis elegans*
MSCSSSSMLFLVLIATTVLIAESRVFYNRFDGGLSSDRFMEQKRDGAEASYDYDANQVIRNTMKRNRQCLLNAGLSQGCD
FSDLLHAQTQARKFMSFAGPGK

SCALIDOPHORA *Priapulus caudatus*
MCRTTLIVFSAFFAVLLVTSTLADDDVIDTARAMEQLALKRSRLLALQQLLKELDNSYETIQKRTCLMNGGMSHSCDYSE
MSRAGDKMRYLTSAISPGRRRRAAAGPSTRLSDLLQLLRSRS

ONYCHOPHORA *Peripatopsis sedgwicki*
MKQLPLTFVWLCLVILYLLQGATSEDLDHRAIRSLVKKRQDLAHLKSLLEEMDNELGMRQKRMCSINLGMNHNCDLSDLL
ISIGQQAHLEGADSPGRRNVRSIP

Supplementary Figure 3. Calcitonin precursors. A number of deduced calcitonin precursors. Predicted signal peptides are highlighted in yellow, the predicted mature calcitonins in light blue, convertase cleavage sites and C-terminal basic amino acid residues expected to be removed by carboxypeptides in red and glycine residues transformed into C-terminal amides in purple. Highlighting in grey is used to indicate a peptide that has some similarity to the consensus sequence but at the same time seems so different that it could be without biological activity. Note that in several species precursors for two or three peptides and that *Tribolium* genome has two calcitonin B genes.

Supplementary Figure 6.
Calcitonin tree

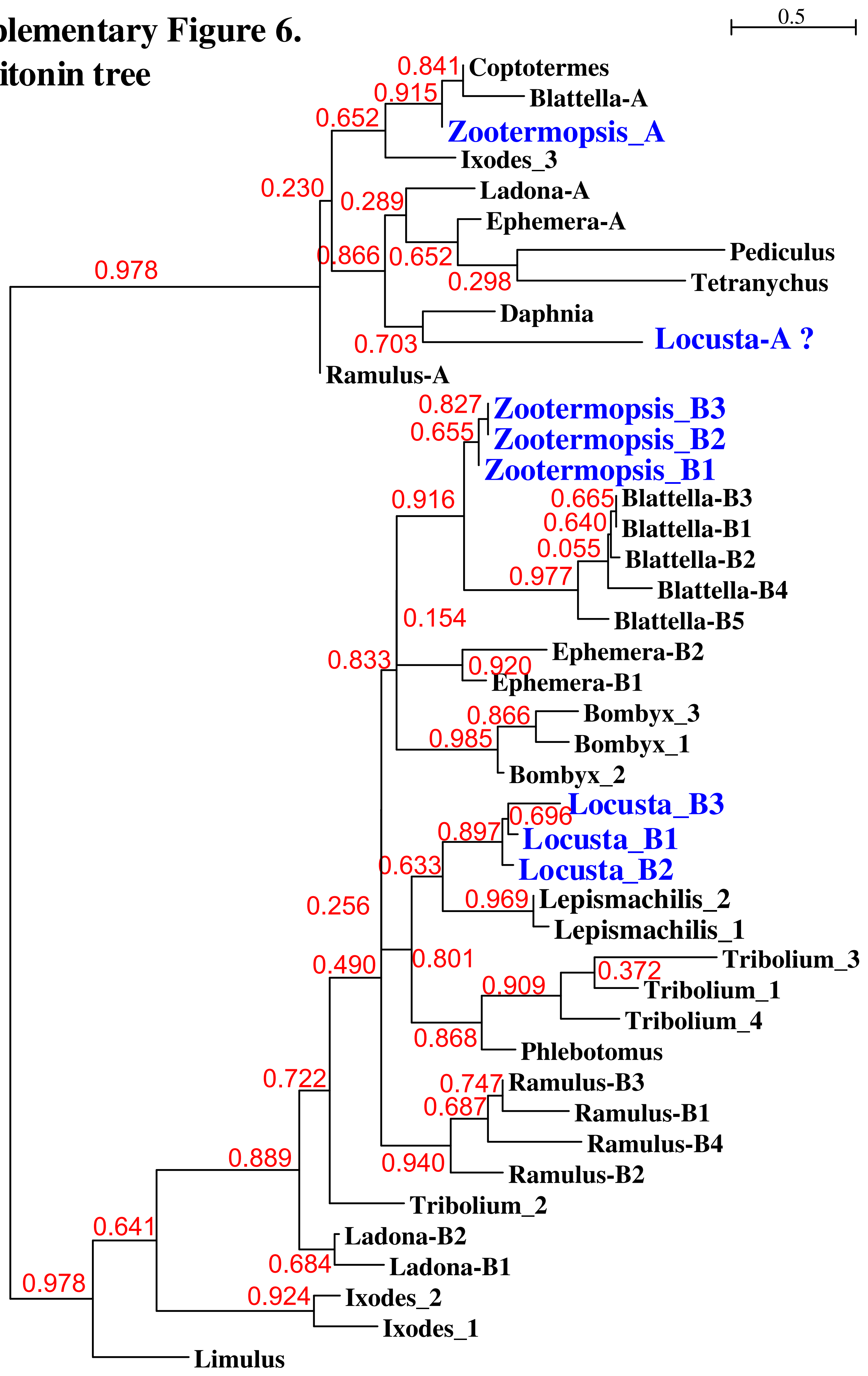

Supplementary Figure 7. Eclosion hormone tree

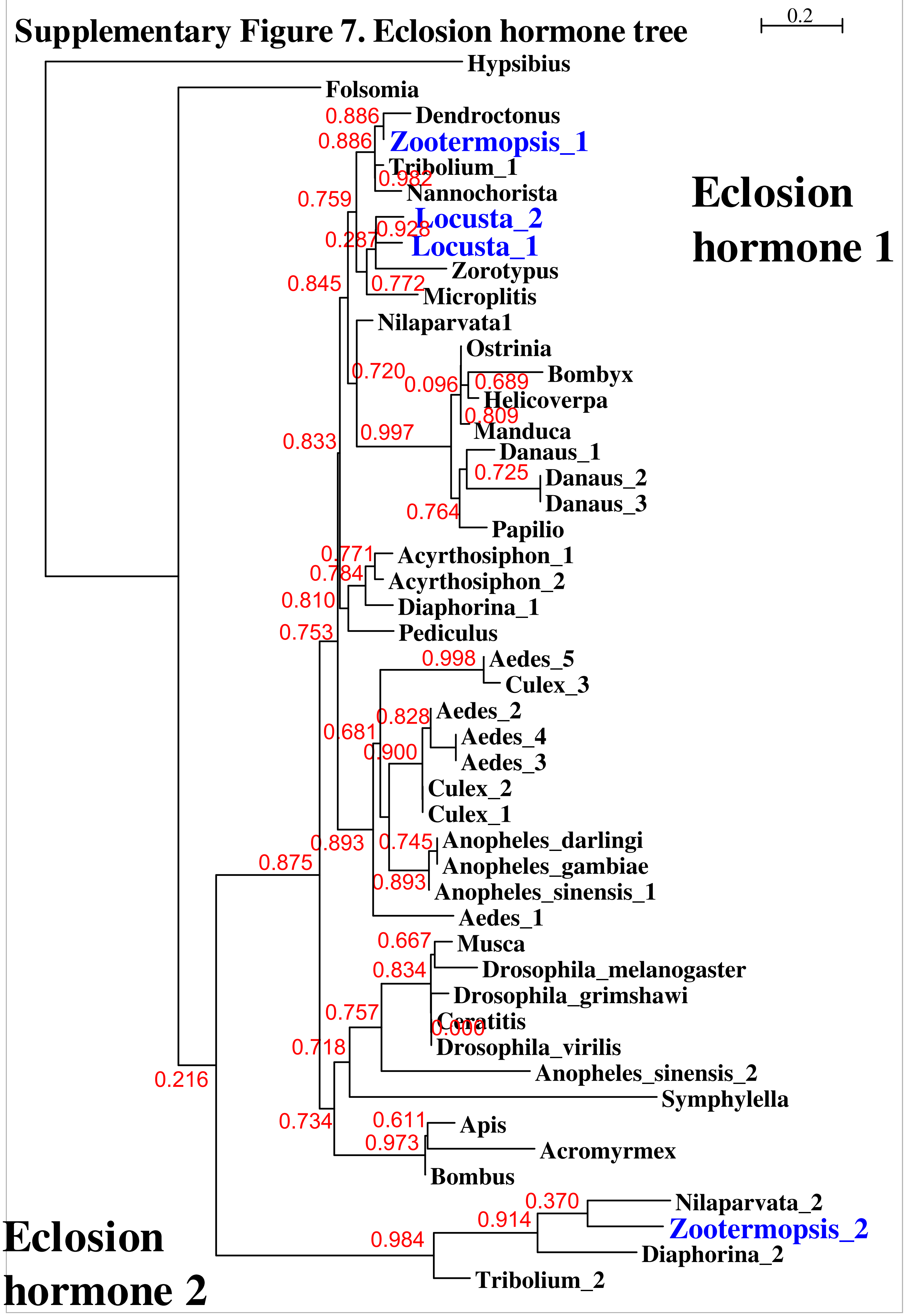

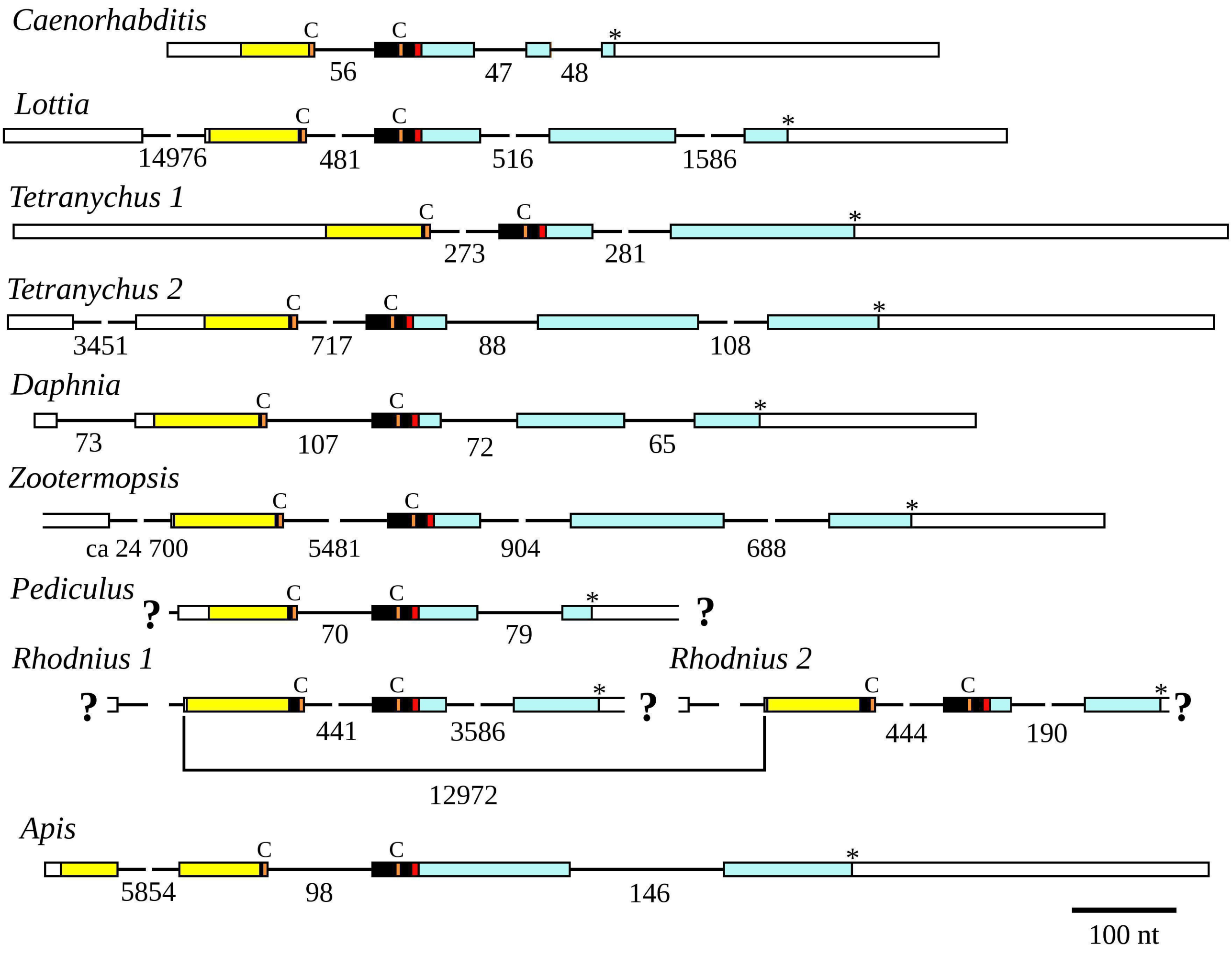

Supplementary Figure 8. Gene structure of elevenin genes in various species. Boxes indicate exons and numbers below the lines the lengths of the introns in nucleotides. White colored boxes correspond to untranslated 5' and 3' sequences, yellow to DNA sequences coding the signal peptide, black the elevenin peptide, orange its cystein residues (also indicated by C's on top of the boxes, red the convertase cleavage sites and light blue the remainder of the elevenin precursors. The asterisks show the location of the stop codons. Note that the structure of the elevenin gene is well conserverd, particularly with respect to the intron within the sequence coding elevenin, the intron donor is always two nucleotides after the first cysteine codon.

```
Locusta                    MWLVARLVQVGVALSLLSGGALGWG-GLFNRFSPEMLSN--------
Zootermopsis                    MRSFLSGTLTVLALLN-SVCAWG-GLFNRFSPEMLSN--------
Diaphorina                      MLCLFISGFMLLQYGVTPSSAWG-GLFNRFSPEMLTN--------
Apis                      MGQKMYTCVALTVVALVSTMHFGVEAWG-GLFNRFSPEMLSN--------
Drosophila      MSIFPRWAAMLLLLGLAHQLDPSLAASASSFASGNAWQRALFGRESRNLMHRRAPFSGAA

Locusta         -------LG-YGGHGYGAYRSSQPLLQRFHNPVEVFQELQ-EDEEPCYGKKCTSNEHCCP
Zootermopsis    -------LG-YGSH--GGYR-AQPFLQQLN-PVEVFQELQ-EDEEPCYGKKCTANEHCCP
Diaphorina      -------LG-YGGH---SFR-AQPFLQDPD--VLSLQEAENELESPCYGKKCTANEHCCP
Apis            -------LG-YGSH--GDHISKSGLYQRPLSTSYGYSYDSLEEVIPCYERKCTLNEHCCP
Drosophila      DLGLDEYLGPYGAVEQEQHQPHPPPQQMRQQTEVYGIVEPLIEDTPCADRPCLLNDDCCP

Locusta         GTVCVDVDGIVGSCLFAYGLKQGELCRRDSDCETGLLCADSAD--GRSCQPPLTN-----
Zootermopsis    GSVCVDVDGIVGSCLFAYGLKQGELCRRDNDCVTGLLCTEIIGE-GRTCQPPTSN-----
Diaphorina      GSVCVDVDGVIGSCLFAYGLRQGELCRRDNDCETGLICIDNGE--RRTCQPPLSN-----
Apis            GSICMNVDGDVGHCVFELGQKQGELCRNDNDCETGLMCAEVAGSETRSCQVPITS-----
Drosophila      SGVCVSTYGE-GKCVYVFG-RQRDLCQGHADCPQGSSCMLVPQEGAWRCEPSVESGGSTS

Locusta         ----------RKQYSEDCTMSSECDISKGLCCQLQRRHRQAPRKV-CSYFKDPLICIGPV
Zootermopsis    ----------KKLYSEDCTMSSECDIGKGLCCQLQRRHRQAARKV-CSYFKDPLICIGPV
Diaphorina      ----------RKQYSEDCSLSSECDMNKGLCCQFQRRHRQAPRKV-CSYFKDPLVCIGPV
Apis            ----------NKLYNEECNVSGECDISRGLCCQLQRRHRQTPRKV-CSYFKDPLVCIGPV
Drosophila      LLEGIFGAKERQPLGSECSSSSDCQVINGMCCQQQRLHHRAAIKLSCGYFRDAFDCVDMV

Locusta         AAD-QVKD-EIEHTAGEKRITGKVASFN---HIRRK
Zootermopsis    ATD-QVKDNNIEHTAGEKRLTGKAVSVNAFGHMHRRK
Diaphorina      ASDLQFKLNDAERTAGEKRLTGKTSMFN---HIRR
Apis            ATD-QIKS-IVQYTSGEKRITGQGNRIFKR-SLKAPFA
Drosophila      GAEHRRN
```

Supplementary Figure 9. Alignment of ITGQN precursors from *Locusta migratoria, Zootermopsis nevadensis, Diaphorina citri, Apis mellifera* and *Drosophila melanogaster.* Note that both the locust and the termite have well conserved ITGQN precursors that likely would yield ITGQN orthologs, that are underligned in the figure.

```
Zootermopsis  --MAGMTATCVLTAFLITVVAGLPSTLLVDAIKAAESEPSTSKASSKLDKDEELPTAVPL
Blattella_*   -------------------------------------------ASTKQEKEEELPTALPI
Locusta_*     ------------------------------------------------------------
Ramulus       -MGRLGAVLLLLGAALAAAGAVPASLLEDTKEAQTHTGAKVKRAQEVMLFGNQQN-----
Tribolium     -MELRWSIRWATLASCLALSFAIPASLVEEIKTNELRNNKVKRAHPQLNVGEHGREVPYY
Apis          MMCDWVWLLLTLCSLLMIVQSLPTNLAEDTKKTEQTMRPKSKRAQEMLMFGNQQNHQPEN
Bombyx        ----MLLFSLTAITAVLAVSAVP----------------TPSNNKDGSTISE-LPENWDQ

Zootermopsis  ATSSTSKTWEDGNKNGHQEQYSNDGKATGQSNIHNTGHKNTQHHQEQNHGDLANQQDYQS
Blattella_*   AISSTSKSW---------EPHGNAGKSSGRSNVQYPGGSRAQFHQELG----SEQHQDQG
Locusta_*     ------------------AAAAGGARASGAGAVD--------------------------
Ramulus       -RPSESAYFQGLKRGDEADDKAASSKFSQDGFQDDEGEEDSSSRQDEG------------
Tribolium     SKPTAIKRGANNLKNPSPEQQSLSDWEQEQSLYQNPDSLADIQSSLYN-------AENPF
Apis          -NPSSS--YSSTAEKRTLAASGLGGLKAALIEEEKPSRSNTLNNAFYD------------
Bombyx        TKDDNRSLFLNKSDKNDLEPYPLALSEEGNQD----------------------------

Zootermopsis  RGKTVSQYEKGYQYGVGKAAHDKHVENALLKSELYGDPGAMNQYRYYGGSNERKRN-QRL
Blattella_*   HGKTVSQYEKGYQYGVGKAALDKHVENALLKSELYGDP--LNQYRYYGGASERKRN-QHL
Locusta_*     -GKTVAQYEKGYLYGAGKAAPDQHVENALLKSELYGDPSAVNQYRYYGGANERRPDGAEG
Ramulus       -GKSPEQYEKGYRYGVGKESLGKHVENALLKSELFGEPSSVNQYRYYGGGERRM---QEA
Tribolium     DDKTIAEYEKGFHYGTNKEKLDEALENAVLKSELYGDPAPLNQYRYYGNDDQRRR-----
Apis          --------RKNYDYGAVNE-LGYEIPQ-VWDNSPYS--------RYYTNEDRRKRSEKSA
Bombyx        ------GYDQTVDQRFDSPQSNGELDNLIMRPELYGEPPAMEGLASAFDLQRRKRGSGTK

Zootermopsis  --------------TYTPPTKR----SYRPDIPFVLPTDDLTSS----------------
Blattella_*   --------------TYQPPSKR----SYRPELPFVMPPDDLTSS----------------
Locusta_*     --------------AFAPPSKRS--SSFRPMVPHAL---ELSGV----------------
Ramulus       --------------GFAPPSKRSGVSSFRTMVPRALP------S----------------
Tribolium     --------------------KRRDARKIRLD-----------------------------
Apis          VASGSSTTIKPSTTSFQSPTSTQ--QSVQTQVKRNVP----IYQ----------------
Bombyx        VGGAGAA-------TKVVTKSGSGKKNLKPEDQAALSPIDLMTQHEAQRRKRGSGTKVGG

Zootermopsis  ----------------------TARLKRDLGLDPEDVLTLLSLWKAEHRVTNDNNPSMDP
Blattella_*   ----------------------RSRLKRDLELDPEDVLTVLSLWEAEHRAKSENNPSIDP
Locusta_*     ----------------------GPRLKRDLGVDPEDVLALLQLWQAERHAAN----RAPS
Ramulus       ----------------------TPRLKRDLDLDPEDVLALLSLWEAEHHKQP-PFRQEQS
Tribolium     -----------------------SRMKREVDLTPDEIFTILTLYENERNGYR--------
Apis          ----------------------EPRFKRELDIDPEDVLTLLSLWENERRKRN--------
Bombyx        AAASAKTATKNSGGNKKNFRPISERRKRDSGLSAADVRALLNLWEAQERRKQ--------

Zootermopsis  SCLNYYGLDIPRSFGEDERNEEVEDDDGDASQTDGGWLEGPVA--NPSTTPHQYWLERHG
Blattella_*   SWFSYYGLDTPDPFQEEELENE---EDDDSSQIDGGWLEGPVA--HPSSSSHRYRLERRG
Locusta_*     KWSRYGNIEGEEYPQAVGNENEEMEEDDSN---NGEWLEGPVY--SSALGPH-YAVDRRA
Ramulus       GWSQFNNGEPSEDFETQEEENQADNEEDNPN--AGGWWEGPVYPSAPSSYPGHFSLDKRV
Tribolium     PW----GLE-PEPSGDNLEEEE-----------N--WLDAPVY-------PH--------
Apis          -WHKYMNEE----YENVDDEDNLLEEEDSRNIIP--WMDSSVY------PPRHYSLDSLS
Bombyx        EYANQFAAD--RYYGRVNPDEEQPEVDENGDL----WYNEPVV-----IGPHDRDYPHHS

Zootermopsis  ----GYHYPILPTA-QQYPLYP---AQKTDSSQWGG-----------F--TKDKRFMVSR
Blattella_*   ----GYYYPL------QYP-YP---TQKRDSSQWGG-----------F--AKDKRFMVTR
Locusta_*     LYIPEYPYQVVGPY-AGYQVQP---DKR--DSRWNG-----------F--SKEKRFMVSR
Ramulus       ----PVYFPANGGG--QFPLFP---AQKR-EGQWGG-----------F--AKEKRFMVSR
Tribolium     ---------ATGHN-DLAPSYL---MDEK-RGRWGG-----------FADSRKKRFMVAK
Apis          PSDIGIIRTHPSSYYEQYENQY---GQQYDTSQYGSPQYGLVYPQQTYYSAPEKRFMISR
Bombyx        YFSEQNRMALARGYPDLYQVGPNELAQRYEEARRKR-----------QYANKMKRFMVAK

Zootermopsis  KRQ--------VTSP-HDGVMTLAQLLDI-PY-RDPGVPMYRHVVL
Blattella_*   KRQ--------VSTP-RDEVQTLAQLLNH-PY-RDPGVPLYRRVVL
Locusta_*     KRD--------MSQPARGDIHYLAQLLGP-SH-RDPQNPLYHRVAA
Ramulus       KRQ--------QAVP-RDGIHSLAQLLNSTPR-REPGMPVLRRMVL
Tribolium     KRNDPTRELRYLNGPNKNDYYTLSQLLSN--Q-REPNVPLYHRLVL
Apis          KRS----------QA-YDPYSNAAQFQLS--S-QSRGYPYQHRLVY
Bombyx        KRSDNMMHQN-NYRP-RDDLYTLAELLRSAPRVQEQDIPVYRRLIL
```

Supplementary Figure 10. Sequence alignment of putative baratin precursors. The part of the sequence which in cockroaches appears similar to baratin (DNSQWGGFA) is under- and over-lined in red.

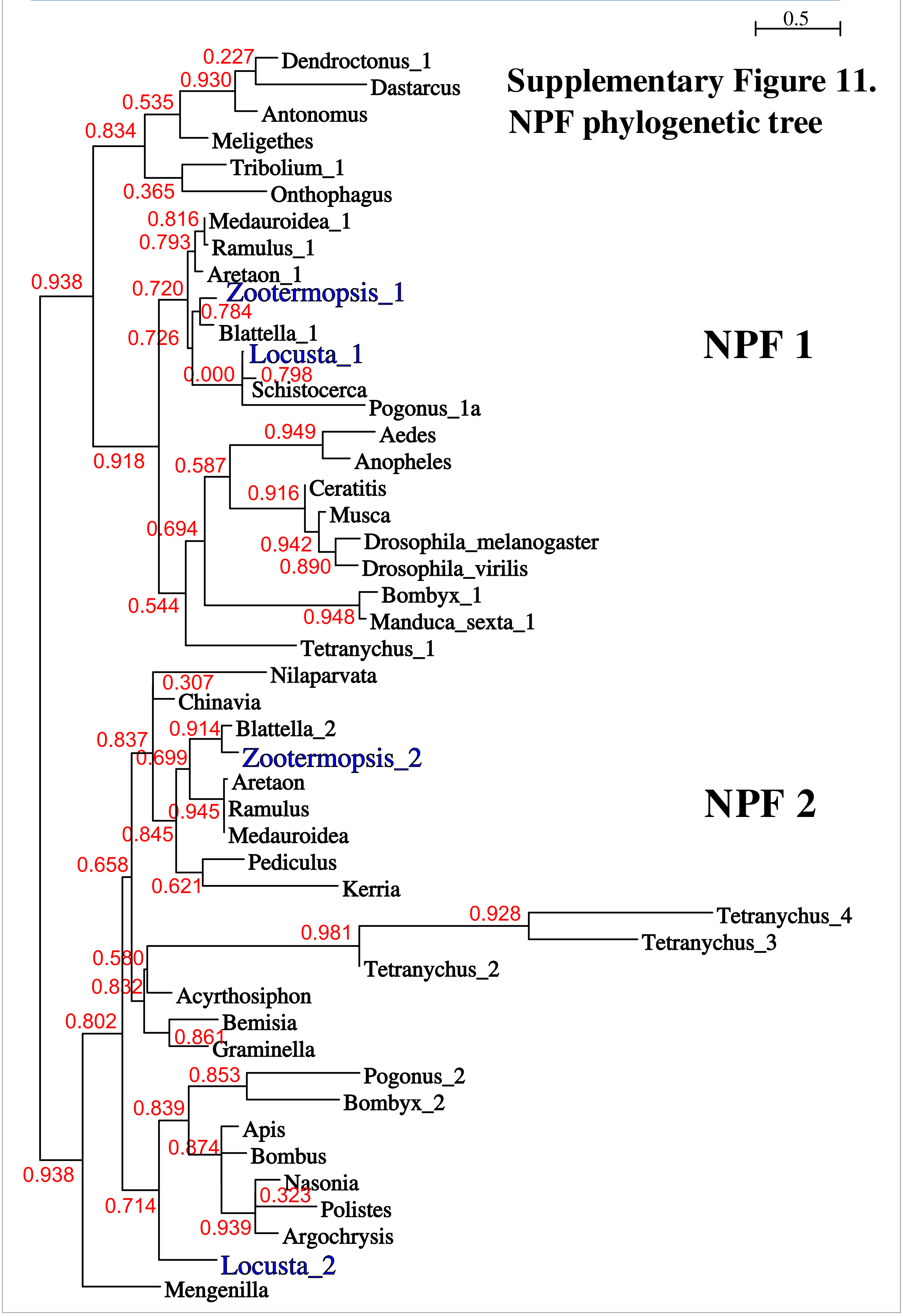
Supplementary Figure 11.
NPF phylogenetic tree
0.5
NPF 1
NPF 2
Dendroctonus_1
Dastarcus
Antonomus
Meligethes
Tribolium_1
Onthophagus
Medauroidea_1
Ramulus_1
Aretaon_1
Zootermopsis_1
Blattella_1
Locusta_1
Schistocerca
Pogonus_1a
Aedes
Anopheles
Ceratitis
Musca
Drosophila_melanogaster
Drosophila_virilis
Bombyx_1
Manduca_sexta_1
Tetranychus_1
Nilaparvata
Chinavia
Blattella_2
Zootermopsis_2
Aretaon
Ramulus
Medauroidea
Pediculus
Kerria
Tetranychus_4
Tetranychus_3
Tetranychus_2
Acyrthosiphon
Bemisia
Graminella
Pogonus_2
Bombyx_2
Apis
Bombus
Nasonia
Polistes
Argochrysis
Locusta_2
Mengenilla
0.227
0.930
0.535
0.834
0.365
0.816
0.793
0.938
0.720
0.784
0.726
0.000
0.798
0.949
0.918
0.587
0.916
0.694
0.942
0.890
0.544
0.948
0.307
0.914
0.837
0.699
0.945
0.845
0.658
0.621
0.928
0.981
0.580
0.832
0.802
0.861
0.853
0.839
0.874
0.323
0.938
0.714
0.939

```
Bombyx        ---------MLSKNLAVVAVAVLLALVCMAEAREEGPN-----NVAEALRI
Tribolium     ------MRWSTLWWFAAVLAAALLQ--------ENGGEAARTSRSNDMLER
Locusta       ------MSQSRPLALLVVAALVAAAVLVAAAEAQQA-DGNKLEGLADALKY
Zootermopsis  ------MQSSLAWFLVVGCALVLMPHVAPTAWAKPT-DPEQLAAMADTLKY
Blattella     ------MQSSLCWLLVVGCTVVLIPYLAPGVWGKSA-DPDQLAAMADTLRY
Ramulus       MSGKTGGRPALRWLLALGCVLALAGQLALA---RPSPDPDQLAAMADALKY

Bombyx        LQLLDNYYTQAARPRIDRRDVDAAGD----------RVDPELLDRAVRLLW
Tribolium     LIKLDEMYSAVARPS-GPTQVDSMGP---------------KVQRAINMLR
Locusta       LQELDRYYSQVARPS-PRSGGAAALPVRSPLDSLSIAEHLRGVEKMVRMLQ
Zootermopsis  LQELDRYYSQVARPS-PRSESGRQ-------------HELSRVENALKMLQ
Blattella     LQELDRYYSQVARPS-PRSGSGRA-------------HELTKVENALKMLQ
Ramulus       LQELDRYYAQVARPS-PRSQSGTTRP-----------HELSKMENAIKMLQ

Bombyx        LEKLDRIYSYHTRPRFGKRSDTYTNWAKDVEKPD---LPTWLTYARRR--
Tribolium     LQHLDRLYADRARPRFGKRGESLHTNI-------------FLSFDEKHTL
Locusta       LQEYDRMYTPRNRPRFGKRAELRPDVVDDVIPEEMSADKFWRRFARRR--
Zootermopsis  LQELDRFYSPRTRPRFGKRADLRPVNDQDLAPDD-SSDRLWRRIASRR--
Blattella     LQELDRFYSPRTRPRFGKRAELRPIPEQESAPVS-TVNKTLR--------
Ramulus       LQELDRLYAQRTRPRFGKRAELRPMSE-DSAVED-SNEKLWHHFPQRR--
```

Supplementary figure 12. Alignment of NPF1 precursors of a number of insect species. Shown are the predicted NPF1b precursors, the sequence underlined in red is absent from the NPF1a precursors.
